# Persicamidines—Unprecedented Sesquarterpenoids with Potent Antiviral Bioactivity against Coronaviruses

**DOI:** 10.1002/anie.202214595

**Published:** 2023-01-04

**Authors:** Lena Keller, Emilia Oueis, Amninder Kaur, Nasim Safaei, Susanne H. Kirsch, Antonia P. Gunesch, Sibylle Haid, Ulfert Rand, Luka Čičin‐Šain, Chengzhang Fu, Joachim Wink, Thomas Pietschmann, Rolf Müller

**Affiliations:** ^1^ Helmholtz Institute for Pharmaceutical Research Saarland (HIPS) Helmholtz Centre for Infection Research (HZI) Saarland University Campus 66123 Saarbrücken Germany; ^2^ Weincampus Neustadt Department of Applied Logistics and Polymer Sciences University of Applied Science Kaiserslautern Carl-Schurz-Straße 10–16 66953 Pirmasens Germany; ^3^ Department of Chemistry Khalifa University PO Box 127788 Abu Dhabi United Arab Emirates; ^4^ German Centre for Infection Research (DZIF), Partner Site Hannover-Braunschweig 38124 Braunschweig Germany; ^5^ Helmholtz Centre for Infection Research (HZI) Inhoffenstr. 7 38124 Braunschweig Germany; ^6^ TWINCORE Centre for Experimental and Clinical Infection Research GmbH Institute for Experimental Virology Feodor-Lynen-Str. 7 30625 Hannover Germany; ^7^ Department of Pharmacy Saarland University 66123 Saarbrücken Germany

**Keywords:** Actinobacteria, Antiviral Agents, Oleandrose, Sesquarterpenes, Structure Elucidation

## Abstract

A new family of highly unusual sesquarterpenoids (persicamidines A–E) exhibiting significant antiviral activity was isolated from a newly discovered actinobacterial strain, *Kibdelosporangium persicum* sp. nov., collected from a hot desert in Iran. Extensive NMR analysis unraveled a hexacyclic terpenoid molecule with a modified sugar moiety on one side and a highly unusual isourea moiety fused to the terpenoid structure. The structures of the five analogues differed only in the aminoalkyl side chain attached to the isourea moiety. Persicamidines A–E showed potent activity against hCoV‐229E and SARS‐CoV‐2 viruses in the nanomolar range together with very good selectivity indices, making persicamidines promising as starting points for drug development.

## Graphical abstract

A new family of highly unusual terpenoids (persicamidines A–E) was isolated. Extensive NMR analysis unraveled a hexacyclic terpenoid with a modified sugar and a highly unusual isourea moiety. Persicamidines showed potent antiviral activity. Each displayed single‐digit nanomolar activity against hCoV‐229E, comparable to that of remdesivir. Particularly Persicamidine C was a very potent inhibitor of SARS‐CoV‐2, with an IC_50_ value of 74 nM.

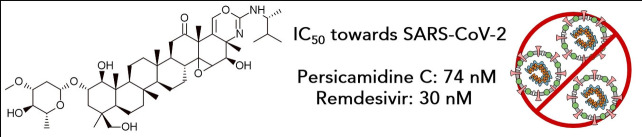

## Introduction

**Figure 1:**
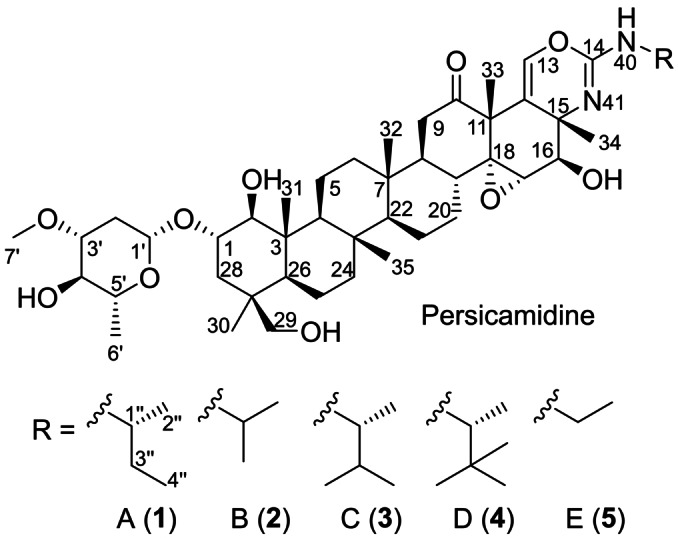
Structures of persicamidines A–E (**1**–**5**).

Natural products are among the major precursors of bioactive and medicinal compounds.^[1]^ Regardless of their nature, the survival needs of species and their innate chemical defense mechanisms against various predators have made them an enormous pool of diverse and structurally complex compounds occupying underexplored regions of the chemical space.^[2]^ Particularly, the secondary metabolites produced by bacteria have diverse functions, including environmental adaptation and survival through the modulation of microbial interactions, competing for nutritional sources, and fighting off predators.^[3]^ Despite the high rediscovery rate often reported, natural products have an enormous genomically encoded potential to produce novel chemical entities. Its exploitation has been facilitated by advances in analytical techniques, genomics technologies, bioengineering strategies, and widely available databases.^[4]^ The exploration of understudied or novel species improves the chances of making related discoveries as evidenced by recent genomic studies.^[5]^ Indeed, a novel strain *Kibdelosporangium persicum* sp. nov. (4NS15), isolated from a neglected hot desert habitat in Kerman, Iran,^[6]^ is the producer of a new family of sesquarterpenoids, a subfamily of terpenoids. Also known as isoprenoids, they constitute the biggest family of natural products and are isolated from various natural sources, but not many from bacteria.^[7]^ These compounds are built from different terpene precursors comprising of two to eight isoprene units n (C_5n_), determining their subfamily, mostly followed by enzymatic modifications and tailoring reactions, including regioselective and stereoselective cyclizations with class I terpene synthases and class II terpene cyclases, prenyl transfer, rearrangements, and derivatizations.^[8]^ The specificity and particularity of each biosynthetic machinery are the reasons for the observed enormous structural diversity of terpenoids. The sesquarterpenoids, persicamidines A‐E, detected from *K. persicum* sp. nov., have a highly unusual structure, a glycosylated multicyclic terpenoid fused to an isourea, which does not resemble any compound known to date (Figure 1). The new compound family contains five derivatives that differ in the composition of the side chain, all of which have been isolated and purified. Herein, we describe the structure elucidation of the new compounds together with their promising antiviral bioactivity against SARS‐CoV‐2 and hCoV‐229E.

A structure similarity search was conducted using Scifinder and Pubchem. The latter showed no results as the lowest similarity percentage could be set at 80 %. The Scifinder search revealed only steroid and terpene glycosides/saponins as compounds with the most similar structures (<72 % similarity index);^[9]^ however, neither of these compounds contained the hexacyclic core or the isourea moiety fused to the terpenoid structure, highlighting the structural novelty of persicamidines.

## Results and Discussion

In the course of a metabolic profiling of the novel strain *Kibdelosporangium persicum* sp. nov. (4NS15) using a combination of NMR and HR‐LCMS data for isolation based on new chemical features, an unknown compound family, named persicamidines A–E (**1**–**5**), was detected. Peaks for five persicamidine derivatives were observed in the crude extract, which were present in varying amounts. These represented a series with a mass difference equivalent to one methylene unit (14 Da). The molecules were isolated from a large‐scale shake flask cultivation of the strain using a combination of preparative and semi‐preparative reversed‐phase HPLC. Persicamidines A and B were the most abundant derivatives with yields of 16.5 and 13.3 mg, respectively, isolated from 10 L cultivation volume.

### Structure Elucidation

To determine the structures of persicamidines, NMR experiments were run in five different solvents (CD_3_OD, DMSO‐*d*_6_ with and without trifluoroacetic acid (TFA), CDCl_3_, and pyridine‐*d*_5_) and countless NMR experiments including *N*‐HSQC, *N*‐HMBC, HSQC‐TOCSY, 1,1‐ADEQUATE, 1,n‐ADEQUATE, LR‐HSQMBC as well as 1D‐ and 2D‐NOESY and ROESY experiments at different mixing times were acquired (only relevant data shown).

HRESIMS of persicamidine A displayed an ion peak at *m*/*z* 813.5264 [*M*+H]^+^ (calcd for C_46_H_73_N_2_O_10_, 813.5260, Δ=0.49 ppm), consistent with the molecular formula C_46_H_72_N_2_O_10_ containing 12 double‐bond equivalents (DBE). The ^1^H NMR spectrum of **1** in DMSO‐*d_6_* (Table S2) was very crowded due to the size of the molecule but showed a clear downfield shifted signal characteristic for an oxygenated methine at *δ*_H‐16_ 4.07 (1*H*, d, *J*=3.5 Hz). The ^13^C spectrum of **1** contained characteristic peaks at *δ* 213.7 and *δ* 149.4 for the keto carbonyl and isourea moiety, respectively. Additional signals at *δ* 125.8 and *δ* 131.2 indicated the presence of two *sp* ^*2*^‐hybridized carbons, while six signals between *δ* 71.3 and *δ* 82.4 were characteristic for oxymethine groups. Analysis of HSQC NMR data further confirmed the presence of these six oxymethines, as well as an oxymethylene group (*δ*_C‐29_ 63.1/*δ*_H‐29a_ 3.34/*δ*_H‐29b_ 3.21). Additionally, one methoxy group (*δ*_C‐7′_ 56.1/*δ*_H3_‐_7′_ 3.30) and one aminomethine proton (*δ*_C‐1′′_ 48.3/*δ*_H‐1′′_ 3.32) together with nine methyl groups were identified. The HSQC data also characterized one of the two *sp* ^*2*^‐hybridized carbons as a methine, indicating a double bond (*δ*_C‐13_ 131.2/*δ*_H‐13_ 5.75). Furthermore, the HSQC spectrum showed three unusual signals indicative of a terpenoid ring structure; the three signals showed an uncommon combination of an upfield proton shift with a downfield shifted carbon resonance (*δ*_C‐4_ 61.7/*δ*_H‐4_ 0.93; *δ*_C‐22_ 51.4/*δ*_H‐22_ 1.03; *δ*_C‐26_ 54.7/δ_H‐26_ 0.86). A detailed analysis of the HSQC, HMBC, COSY, and TOCSY NMR data revealed a highly functionalized hexacyclic terpenoid.

The mass spectra of all persicamidine analogues showed a characteristic loss of 144 mass units, indicating the presence of a sugar moiety. The HSQC spectrum of persicamidine A confirmed the assumption showing a signal characteristic for an anomeric proton (*δ*_C‐1′_ 100.5/*δ*_H‐1′_ 4.56). A sequential spin system starting from the anomeric proton attached to a methylene at *δ* 2.38 and 1.10 (H_2_‐2′) comprising three oxymethines H‐3′ to H‐5′ and a methyl group at *δ* 1.14 (H_3_‐6′) was identified using COSY and TOCSY correlations. As indicated by the downfield shifted carbon resonance of C‐3′, HMBC correlations between oxymethine CH‐3′ and the methoxy group (CH_3_‐7′) completed the structure of the sugar moiety, 4‐methoxy‐6‐methyloxane‐2,5‐diol. Analysis of the ROESY NMR data afforded the relative stereochemistry, and the sugar residue was finally identified as oleandrose. The HMBC spectrum showed a key correlation from the anomeric proton at *δ* 4.56 (H‐1′) to the oxymethine at *δ* 78.1 (C‐1) indicating an *O*‐linked glycosylation of the terpenoid skeleton with β‐configuration as deduced from ROESY NMR data.

**Figure 2:**
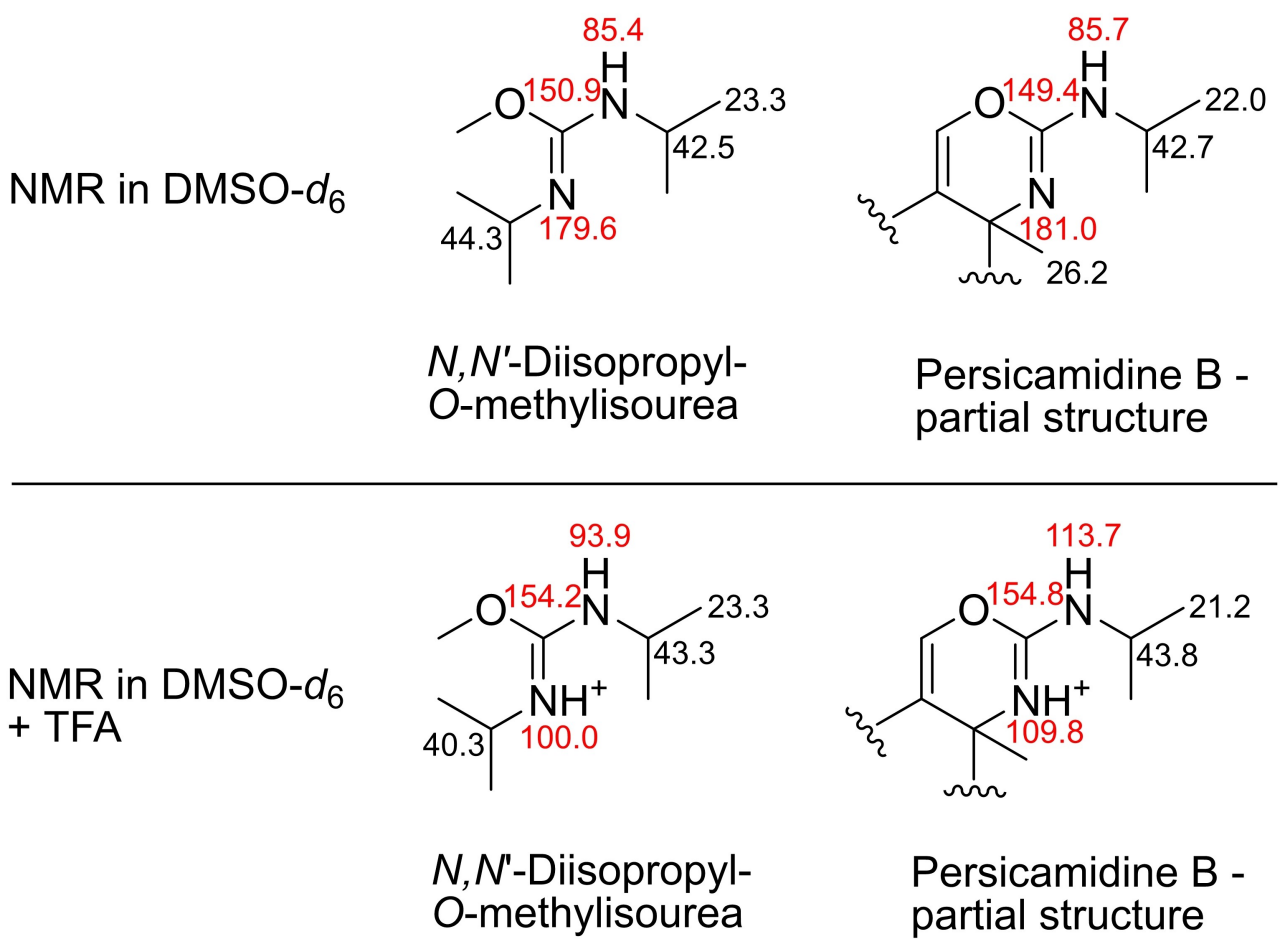
Comparison of ^15^N and ^13^C NMR chemical shifts of a synthetic compound, *N*,*N*′‐diisopropyl‐*O*‐methylisourea, and persicamidine B with and without the addition of TFA in DMSO‐*d*_6_. Highlighted in red are the significant chemical shifts that allowed the unambiguous assignment of the isourea moiety.

In addition, the hexacyclic terpenoid skeleton was attached to a structural moiety that bridged the connection to a short alkylamine side chain. Due to a high heteroatom content in combination with a low proton amount, the elucidation of this structural unit was highly complex and several structural possibilities were considered. Considering the molecular formula, the remaining atoms that were still unaccounted for included CON_2_H with 2 degrees of unsaturation. *N*‐HSQC and *N*‐HMBC data revealed the nature of the two nitrogen atoms as an amine (*δ*_N‐40_ 85.7) and a urea or amide (*δ*_N‐41_ 181.0). Based on the HMBC and *N*‐HMBC data, both nitrogen atoms were separated by a quaternary carbon at *δ* 149.4, to which the *sp* ^*2*^‐hybridized methine (*δ*_C‐13_ 131.2/*δ*_H‐13_ 5.75), aminomethine (*δ*_C‐1′′_ 48.3/*δ*_H‐1′′_ 3.32), and oxymethine (*δ*_C‐16_ 73.1/*δ*_H‐16_ 4.07) also showed correlations in the HMBC data. The amine nitrogen atom was attached to the alkyl side chain showing a very weak COSY correlation to the aminomethine. The signal of the amine proton was very weak, most likely due to delocalization. The second nitrogen atom showed *N*‐HMBC correlations from a methyl group (*δ*_C‐34_ 26.3/*δ*_H3‐34_ 0.99) and an oxymethine (*δ*_C‐16_ 73.1/*δ*_H‐16_ 4.07). The structural feature was therefore confirmed as an isourea moiety fused to the terpenoid structure. Due to the weak signal of the amine proton, the unusual arrangement of the moiety with a lack of characteristic proton and carbon chemical shifts and the missing literature values for the nitrogen chemical shifts, the NMR data of persicamidine B were compared to the isourea‐containing commercial standard, *N*,*N*′‐diisopropyl‐*O*‐methylisourea, with and without the addition of 0.1 % trifluoroacetic acid. The standard did not only show similar nitrogen and carbon chemical shifts compared to persicamidine B but also a very similar pattern of change in the chemical shifts after adding trifluoroacetic acid (TFA) into the NMR tube (Figure 2), allowing the unequivocal confirmation of the 2D structure of the persicamidines. Recovering the samples from the NMR measurements with TFA, we were also able to isolate the aglycon of persicamidine B that provided a slightly simplified NMR spectrum and confirmed the persicamidine structure (Table S2).

Highly similar NMR data sets for persicamidines B–E (**2**–**5**) were recorded and analyzed, and the difference pinpointed to the alkyl side chain attached to the isourea moiety (Tables S2, S3). The five derivatives represented a series with a mass difference equivalent to one methylene unit each (Figure S1). HRESIMS data of persicamidine B (**2**) displayed an ion peak at *m*/*z* 799.5103 [*M*+H]^+^ (calcd for C_45_H_71_N_2_O_10_, 799.5103, Δ=0.00 ppm), consistent with the molecular formula C_45_H_70_N_2_O_10_ containing 12 double‐bond equivalents (DBE) and the presence of an *iso*‐propyl group instead of *sec*‐butyl in **1**. Persicamidine C (**3**) showed an ion peak at *m*/*z* 827.5414 [*M*+H]^+^ (calcd for C_47_H_75_N_2_O_10_, 827.5416, Δ=0.24 ppm), corresponding to the molecular formula C_47_H_74_N_2_O_10_. Analysis of the NMR data revealed the presence of the 1,2‐dimethylpropyl side chain. An increase of 14 mass units was observed in the HRESIMS data of persicamidine D, showing an ion peak at *m*/*z* 841.5571 [*M*+H]^+^ (calcd for C_48_H_77_N_2_O_10_, 841.5573, Δ=0.24 ppm). The corresponding molecular formula (C_48_H_76_N_2_O_10_) as well as 1D and 2D NMR data analysis allowed the identification of the 3,3‐dimethylbutan‐2‐amino group. The NMR and MS data of persicamidine E [*m*/*z* 785.4943 [*M*+H]^+^; calcd for C_44_H_69_N_2_O_10_, 785.4947, Δ=0.51 ppm] were consistent with the attachment of the ethyl group to N‐40.

The relative stereochemistry of persicamidines was determined by ROESY/NOESY data analysis. As mentioned previously, the sugar moiety was determined to be oleandrose based on its relative stereochemistry. ROESY correlations were observed from the anomeric proton H‐1′ (*δ* 4.56) to the oxymethines H‐3′ (*δ* 3.04) and H‐5′ (*δ* 3.10) and to one of the methylene protons H‐2′_a_ (*δ* 2.38), from the methyl group H_3_‐6′ (*δ* 1.14) to the oxymethine H‐4′ (*δ* 2.79), and from H‐4′ to the other methylene proton H‐2′_b_ (*δ* 1.10), while no correlation was observed between H‐3′ and H‐4′. This also indicated the beta configuration of the sugar moiety.

**Figure 3:**
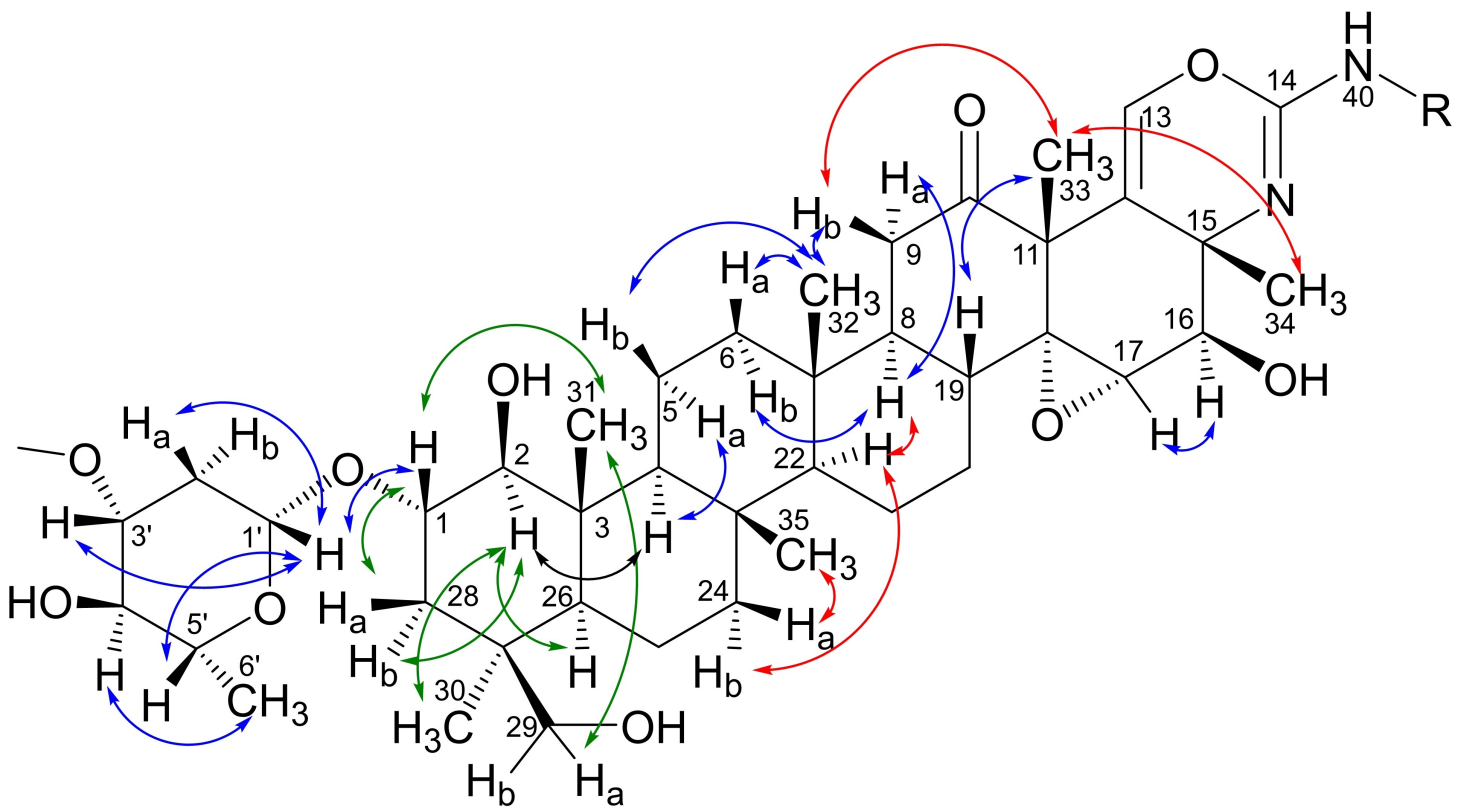
Key ROESY/NOESY correlations identified by analysis of NMR data of persicamidines A and B in CD_3_OD (↔), DMSO‐*d*_6_ (↔), and CDCl_3_ (↔).

Due to extensive overlap of the methyl and methine signals, ROESY/NOESY data of **1** and **2** acquired in three different solvents (CD_3_OD, DMSO‐*d*_6_, and CDCl_3_) were used to determine the relative configuration of the terpene‐derived core of persicamidines, the summation of which is shown in Figure 3. The ^1^H NMR signals for CH_3_‐31 and CH_3_‐30 were overlapping or in close proximity in CD_3_OD and DMSO‐*d*_6_. Therefore, ROESY data of **1** and **2** were collected in CDCl_3_, where these signals were sufficiently resolved, and clear correlations between H_3_‐31 (*δ* 0.96), H‐1 (*δ* 3.61), and H‐29a (*δ* 3.69), as well as between H‐28a (*δ* 2.28) and H‐1 allowed their placement on the same side of the ring system, while those between H‐2 (*δ* 3.20) and H‐26 (*δ* 1.02), H‐28b (*δ* 1.14), and H_3_‐30 (*δ* 0.99) positioned these on the opposite face of the ring. In DMSO‐*d*_6_, additional correlations were observed between H‐2 (*δ* 2.95) and H‐4 (*δ* 0.93), and further from H‐4 to H‐5a (*δ* 2.57), whereas sequential NOESY correlations were identified between H‐5b (*δ* 1.31) and H_3_‐32 (*δ* 0.76), and from H_3_‐32 to H‐6_a_ (*δ* 1.70) and H‐9_b_ (*δ* 2.19), placing the two sets of protons *anti* to each other. H‐9_a_ (*δ* 2.69) showed a NOESY correlation to H‐8 (*δ* 2.12, t, 9–10 Hz), indicating their *syn* orientation, while a large coupling constant of 9–10 Hz for the triplet observed for H‐8 established the *trans*‐diaxial orientation of H‐8 and H‐19 (*δ* 1.57). In CD_3_OD, well‐resolved ROESY correlations between H‐9_b_ (*δ* 2.40) and H_3_‐33 (*δ* 1.59), as well as between H_3_‐33 and H_3_‐34 (*δ* 1.31) placed these on the same side of the ring system. To determine the relative configuration of H‐22 (*δ* 1.13) and CH_3_‐35 (*δ* 0.93), the ROESY data in CD_3_OD were further analyzed. Correlations between H‐8 (*δ* 2.28) and H‐22 (*δ* 1.13), as well as between H‐22 and H‐24_b_ (*δ* 0.88) indicated their *syn* orientation, whereas that between H‐24_a_ (1.67) and H_3_‐35 placed these on the opposite face.

The absolute configuration of H‐16 was independently determined to be *R* via analysis using Mosher's method.^[10]^ A strong ROESY correlation between H‐16 (*δ* 4.07) and H‐17 (*δ* 3.21) was observed in DMSO‐*d*_6_, which initially suggested their *syn* orientation. However, analysis of the energy‐minimized 3D structures obtained using Chem3D and Spartan software showed that the distance between H‐16 and H‐17 was <2.9 Å, irrespective of the *R‐* or *S*‐configuration at position 17. Therefore, analysis of the ROESY correlations between these two protons was not reliable to establish the relative configuration of H‐17. Additionally, very weak/lack of ROESY correlations between H‐16/H‐17 and H_3_‐34, as well as between H‐17 and H_3_‐33 did not allow any conclusive assignment. Considering the ambiguity in the relative configuration assignment of position 17, several unsuccessful crystallization attempts were made using different solvent combinations, as well as derivatization methods. Despite serious efforts, this approach was not successful. However, an attempt to produce the aglycon of persicamidine A (via hydrolysis using 1 N HCl at 100 °C for 1.5 h) yielded a rearranged aglycon formed via Payne‐type rearrangement (Figure S5).[^11^, ^12^] Under acidic conditions, OH‐16 (nucleophile) attacks the more substituted carbon C‐18 to open the epoxide and form an oxetane ring, thereby generating a hydroxyl group at position 17, whose configuration should be retained and similar to that of the epoxide.^[12]^ This rearrangement is consistent with *anti*‐orientation of the epoxide with respect to OH‐16 in persicamidines. Attempts to confirm the absolute configuration of OH‐17 using Mosher's esterification method were unsuccessful as the reaction only yielded a diacylated product, where OH‐17 did not react. However, the observed NOESY correlations as well as ^3^ *J*_H_^−^_16/H‐17_ (5.6 Hz) value were consistent with the conformation adopted by this molecule with (*S*)‐configuration of C‐17 (Figure S5).

The absolute configuration of persicamidines was determined using Mosher's esterification method.^[10]^ Persicamidines A (**1**) and B (**2**) were individually reacted with both (*S*)‐ and (*R*)‐(‐)‐α‐methoxy‐α‐(trifluoromethyl)phenylacetyl chlorides (MTPA‐Cl) to afford the corresponding triacylated (*R*)‐ and (*S*)*‐*Mosher esters, respectively, where the hydroxyl groups at positions 4′, 16, and 29 were acylated. The differences in the chemical shifts (Δ*δ* ^SR^ (=*δ* ^S^−*δ* ^R^)) of the Mosher esters of **1** and **2** are shown in Figures S2 and S3. This allowed the assignment of *R* configuration for the stereocenters at positions 4′ and 16 of compounds **1** and **2**. The advanced Marfey method was used to independently determine the (*R*)‐configuration of the aminoalkyl side chain.^[13]^ It was elucidated by MS detected chromatographic analysis of the l‐ and d‐FDLA (1‐fluoro‐2,4‐dinitrophenyl‐5‐l/d‐leucinamide) derivatives of the acid hydrolysate of **1** together with the respective standards (*S*)‐ and (*R*)‐2‐aminobutane. Identical configurations for **1**–**5** were assumed at comparable chiral centers based on the similarities of their structures and NMR data.

This data in conjunction with the relative configuration assignment using ROESY/NOESY NMR data afforded the absolute configuration of this class of compounds.

### Biological Activity

**Figure 4:**
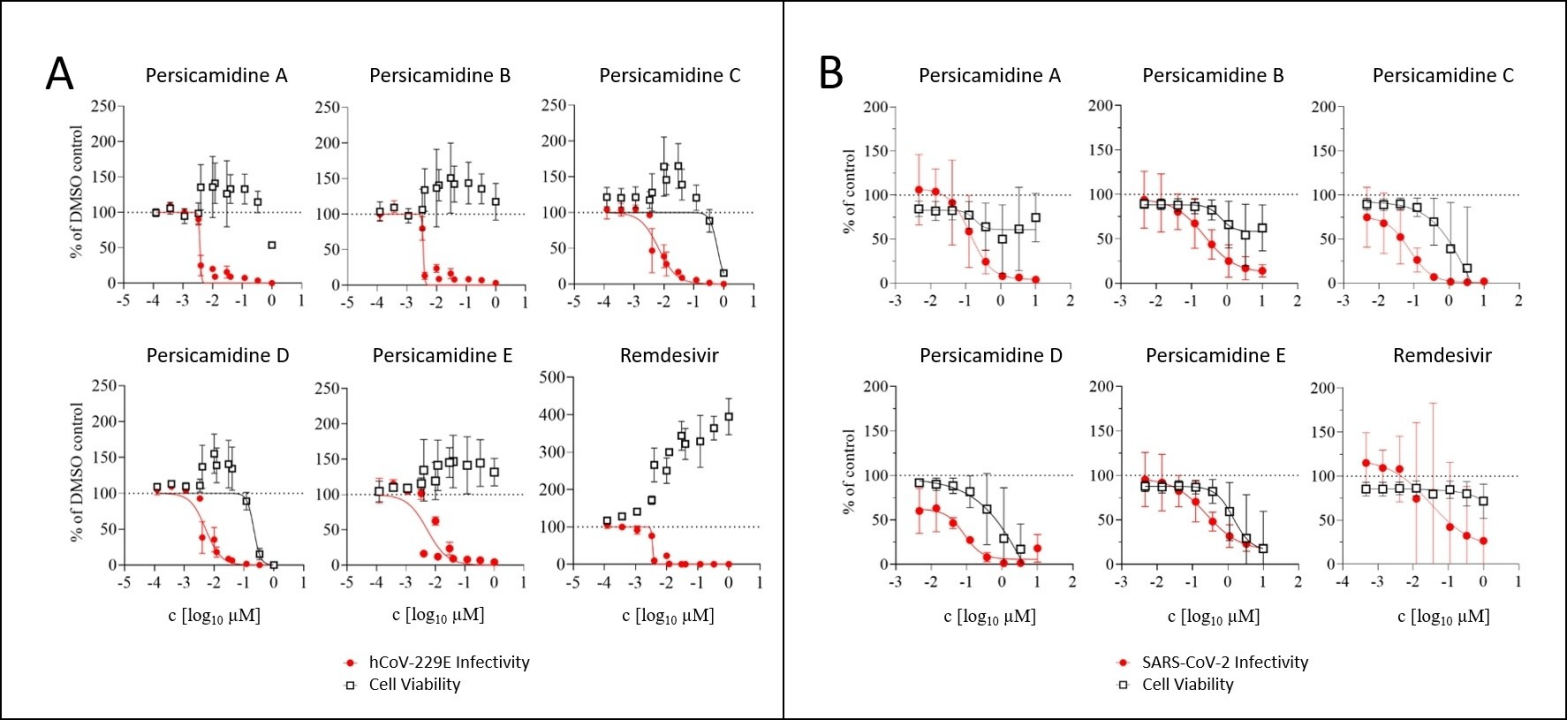
Antiviral activities of persicamidines A–E against A) hCoV‐229E and B) SARS‐CoV‐2 viruses. Displayed are the viral replications (red) with simultaneous determinations of the cell viabilities of Huh‐7.5_FLuc or A549_ACE2_TMPRSS2 cells, respectively (black). Remdesivir was used as a positive control. Mean values of two to five biological replicates (due to varied concentration ranges) containing duplicates and standard deviations are depicted for (A), mean values of three biological replicates and standard deviations are depicted for (B). Data was normalized against a DMSO control (hCoV‐229E) or puromycin (SARS‐CoV‐2) assay. Regression curves were calculated with GraphPad Prism 9.

Compounds **1**–**5** were found to be highly biologically active and each displayed a single digit nanomolar activity against hCoV‐229E, comparable to the positive control, remdesivir (Figure 4; Table 1). For the hCoV‐229E virus, compound **2**, which was the most active derivative, exhibited an IC_50_ of 3.6 nM (IC_50_ remdesivir: 3.5 nM). Furthermore, particularly compounds **3** and **4** were also very potent inhibitors of SARS‐CoV‐2 and exhibited IC_50_ values of 74 and 84 nM, respectively. These values are comparable to the IC_50_ value of the remdesivir control (Figure 4; Table 1).^[14]^ Compounds **1**–**5** had a very good selectivity index in the hCoV‐229E assay with CC_50_ concentrations surpassing the IC_50_ values by 40 to >280. Similarly, compounds **3** and **4** also displayed a favorable selectivity index in the SARS‐CoV‐2 infection assay with a difference between CC_50_ and IC_50_ of >50 and >80, respectively. Therefore, compounds **3** and **4** are broadly active against two human pathogenic coronaviruses, which represent two different genera (i.e. *Alpha*‐ and *Betacoronavirus*) of the subfamily *Coronavirinae*. They exhibited very good selectivity indices against these viruses in two different cellular systems. These features should permit selection of drug‐resistant viruses and in turn identification of the viral target protein. In addition, as persicamidines are novel compounds with an unprecedented skeleton, these warrant a more extensive biological evaluation including *in vivo* cytotoxicity and PK/PD studies. The possibility of generating new derivatives with further optimization of the already existing excellent antiviral efficacy in the nanomolar range and a simultaneous reduction in cytotoxicity, and thus a broadening of the application window, makes persicamidines exceptionally promising for the development of drug candidates. Initial steps towards biosynthesis and total synthesis have already been started, which will enable us to perform the mode‐of‐action and SAR studies in the near future.

**Table 1:** Antiproliferative and antiviral activities of Persicamidines A–E.

Test organism	Persicamidine A	Persicamidine B	Persicamidine C	Persicamidine D	Persicamidine E	Remdesivir (control)
	CC_50_ [μM]					
Huh‐7.5_FLuc cells	>1	>1	0.61	0.21	>1	>1
A549_ACE2_TMPRSS2 cells	>10	>10	3.9	6.96	1.44	>1
	IC_50_ [μM]					
hCoV‐229E	0.0038	0.0036	0.0066	0.0052	0.0053	0.0035
SARS‐CoV‐2	0.13	0.23	0.074	0.084	0.25	0.03

Half‐maximal inhibitory (IC_50_) and cytotoxic (CC_50_) concentrations were calculated with GraphPad Prism 9 using two to five biological replicates (depending on concentration range) for the hCoV‐229E assay and three biological replicates for the SARS‐CoV‐2 assay. Data are mean values.

## Conclusion

A new class of antiviral compounds, persicamidines A–E (**1**–**5**), was isolated from a novel actinobacterial strain, *Kibdelosporangium persicum* sp. nov., collected from a hot desert in Iran. The structures of compounds **1**–**5** were determined by extensive NMR and MS analysis, as well as chemical derivatization procedures. Persicamidines constitute a common hexacyclic terpenoid core flanked by an oleandrose unit and a highly unusual isourea moiety fused to the terpenoid. Closer scrutiny of these chemical structures reveals that seven isoprene units are required to build the rare C35 sesquarterpene backbone.^[15]^ Although a few sesquarterpenes have been discovered from *Bacillus* and *Mycobacteria* before,^[15]^ the 6/6/6/6/6/6‐fused hexacyclic ring system of persicamidines is unprecedented, which might indicate it to be formed by an unusual terpene cyclase. The terpene skeleton is further expanded to the heptacyclic fused ring system by coupling with a urea molecule to establish the unusual heterocycle 2‐amino‐1,3‐oxazine, the biochemistry of which deserves investigation in the future. Intriguingly, it is scarce for terpene biosynthesis that the fused ring system undergoes intensive modifications to form persicamidines, including multiple steps of oxidation, demethylation, methylation, and glycosylation, which require many tailoring and precursor biosynthesis enzymes. At least six oxidation modifications are needed to form the hydroxyl, epoxide, and ketone moieties. According to the previously known demethylation mechanisms of terpenes from bacteria, an oxidase and a dehydrogenase might be required for the demethylation at C‐19 of persicamidines.^[16]^ The biosynthesis of persicamidines thus represents one of the most complicated terpene biosynthetic pathways, which certainly warrants further in‐depth study. The astonishing activity against the SARS‐CoV‐2 virus will also motivate efforts towards total synthesis of this intriguing natural product that will eventually enable mode of action and SAR studies to determine the precise biochemistry behind the antiviral effect. The first description of persicamidines in this publication is thus expected to serve as the starting point for a number of chemical and biological follow up studies.

## Conflict of interest

The authors declare no conflict of interest.

1

## Supporting information

As a service to our authors and readers, this journal provides supporting information supplied by the authors. Such materials are peer reviewed and may be re‐organized for online delivery, but are not copy‐edited or typeset. Technical support issues arising from supporting information (other than missing files) should be addressed to the authors.

Supporting Information

## Data Availability

The data that support the findings of this study are available from the corresponding author upon reasonable request.

## References

[1] A. G. Atanasov , S. B. Zotchev , V. M. Dirsch , C. T. Supuran , Nat. Rev. Drug Discovery 2021, 20, 200. 33510482 10.1038/s41573-020-00114-z PMC7841765

[2] O. Mosunova , J. C. Navarro-Muñoz , J. Collemare in Encyclopedia of mycology (Eds.: O. Zaragoza , A. Casadevall ), Elsevier, Amsterdam, 2021, pp. 458–476.

[3] A. M. Sharrar , A. Crits-Christoph , R. Méheust , S. Diamond , E. P. Starr , J. F. Banfield , mBio 2020, 11, e00416-20. 32546614 10.1128/mBio.00416-20 PMC7298704

[4] M. Miethke , M. Pieroni , T. Weber , M. Brönstrup , P. Hammann , L. Halby , P. B. Arimondo , P. Glaser , B. Aigle , H. B. Bode , et al., Nat. Chem. Rev. 2021, 5, 726. 10.1038/s41570-021-00313-1 PMC8374425 34426795

[5a] R. Subramani , D. Sipkema , Mar. Drugs 2019, 17, 249; 31035452 10.3390/md17050249 PMC6562664

[5b] Y.-H. Chen , P.-W. Chiang , D. Y. Rogozin , A. G. Degermendzhy , H.-H. Chiu , S.-L. Tang , Commun. Biol. 2021, 4, 996. 34426638 10.1038/s42003-021-02510-6 PMC8382752

[6] N. Safaei , I. Nouioui , Y. Mast , N. Zaburannyi , M. Rohde , P. Schumann , R. Müller , J. Wink , Int. J. Syst. Evol. Microbiol. 2021, 71, 004625. 10.1099/ijsem.0.004625 33427607

[7] T. Dairi in Reference module in chemistry, molecular sciences and chemical engineering (Ed.: J. Reedijk ), Elsevier, Oxford, 2014.

[8] E. Oldfield , F.-Y. Lin , Angew. Chem. Int. Ed. 2012, 51, 1124; 10.1002/anie.201103110 PMC3769779 22105807

[9a] T. Fujioka , K. Yoshida , H. Shibao , T. Nagao , M. Yoshida , K. Matsunaga , J. Takata , Y. Karube , Y. Iwase , H. Okabe et al , Chem. Pharm. Bull. 2006, 54, 1694; 10.1248/cpb.54.1694 17139105

[9b] K. K. Purushothaman , S. Vasanth , J. D. Connolly , D. S. Rycroft , Rev. Latinoam. Quim. 1988, 19, 28;

[9c] B. Liang , L. Zhang , J. Tian , L. Xu , S. Yang , Carbohydr. Res. 2006, 341, 2444; 16870168 10.1016/j.carres.2006.06.020

[9d] E. Grosjean , J. Poinsot , A. Charrié-Duhaut , S. Tabuteau , P. Adam , J. Trendel , P. Schaeffer , P. Albrecht , J. Connan , D. Dessort , Chem. Commun. 2000, 923.

[10] T. R. Hoye , C. S. Jeffrey , F. Shao , Nat. Protoc. 2007, 2, 2451. 17947986 10.1038/nprot.2007.354

[11] J. A. Bull , R. A. Croft , O. A. Davis , R. Doran , K. F. Morgan , Chem. Rev. 2016, 116, 12150. 27631342 10.1021/acs.chemrev.6b00274

[12] V. B. Birman , S. J. Danishefsky , J. Am. Chem. Soc. 2002, 124, 2080. 11878938 10.1021/ja012495d

[13] K. Fujii , Y. Ikai , H. Oka , M. Suzuki , K. Harada , Anal. Chem. 1997, 69, 5146.

[14a] M. Widera , A. Wilhelm , T. Toptan , J. M. Raffel , E. Kowarz , F. Roesmann , F. Grözinger , A. L. Siemund , V. Luciano , M. Külp , et al., Front. Microbiol. 2021, 12, 701198; 34394046 10.3389/fmicb.2021.701198 PMC8362758

[14b] X. Xie, A. Muruato, K. G. Lokugamage, K. Narayanan, X. Zhang, J. Zou, J. Liu, C. Schindewolf, N. E. Bopp, P. V. Aguilar, et al., *Cell Host Microbe* **2020**, *27*, 841.e3. 10.1016/j.chom.2020.04.004 PMC7153529 32289263

[15] T. Sato , Biosci. Biotechnol. Biochem. 2013, 77, 1155. 23748782 10.1271/bbb.130180

[16a] J.-M. Lv , D. Hu , H. Gao , T. Kushiro , T. Awakawa , G.-D. Chen , C.-X. Wang , I. Abe , X.-S. Yao , Nat. Commun. 2017, 8, 1644; 29158519 10.1038/s41467-017-01813-9 PMC5696383

[16b] A. K. Lee , A. B. Banta , J. H. Wei , D. J. Kiemle , J. Feng , J.-L. Giner , P. V. Welander , Proc. Natl. Acad. Sci. USA 2018, 115, 5884. 29784781 10.1073/pnas.1802930115 PMC6003346

