# Supplementary material for: Persicamidines—Unprecedented Sesquarterpenoids with Potent Antiviral Bioactivity against Coronaviruses
Source: Angew Chem Int Ed Engl. 2023 Jan 4;62(6):e202214595. doi: 10.1002/anie.202214595 (PMC10107436; doi:10.1002/anie.202214595)
Supplement: Supplementary file 1 — Supporting Information [file ANIE-62-0-s001.pdf]

Angewandte Chemie
Eine Zeitschrift der Gesellschaft Deutscher Chemiker

Supporting Information

Persicamidines—Unprecedented Sesquarterpenoids with Potent Antiviral Bioactivity against Coronaviruses

*L. Keller, E. Oueis, A. Kaur, N. Safaei, S. H. Kirsch, A. P. Gunesch, S. Haid, U. Rand, L. Čičin-Šain, C. Fu, J. Wink, T. Pietschmann, R. Müller**

Table of Contents

Table of Contents 2
List of Figures 2
List of Tables 3
S1. General Information and Materials 4
S2. Experimental Procedures 5
Cultivation, isolation, and purification of strain 4NS15T 5
Metabolite identification 5
S3. Chemical Derivatization Methods 6
Analysis Using Advanced Marfey's Method 6
Mosher esterification of persicamidines A and B 7
Acidic hydrolysis of persicamidine A 8
S4. Biological evaluation of persicamidines A-E 9
hCoV-229E virus infection assay 9
SARS-CoV-2 infection assay 9
S5. NMR data 10
NMR tables 10
NMR figures 17
References 46
Author Contributions 46

List of Figures

Figure S1. LCMS spectrum of the methanol extract of *Kibdelosporangium persicum* sp. nov., and MS^2 spectra of persicamidines A-E (**1**-**5**) 6
Figure S2. Differences in chemical shifts ($\Delta\delta^{SR}$ ($=\delta^{S} - \delta^{R}$)) of Mosher esters of persicamidine A 7
Figure S3. Differences in chemical shifts ($\Delta\delta^{SR}$ ($=\delta^{S} - \delta^{R}$)) of Mosher esters of persicamidine B 8
Figure S4. Differences in chemical shifts ($\Delta\delta^{SR}$ ($=\delta^{S} - \delta^{R}$)) of the Mosher esters of the cleaved glycon derivative 8
Figure S5. Persicamidine A aglycon formed via Payne-type rearrangement 9
Figure S6. ^{1}H NMR spectrum of persicamidine A (**1**) in CD_3OD (700 MHz) 17
Figure S7. ^{13}C NMR spectrum of persicamidine A (**1**) in CD_3OD (175 MHz) 17
Figure S8. COSY NMR spectrum of persicamidine A (**1**) in CD_3OD (700 MHz) 18
Figure S9. TOCSY NMR spectrum of persicamidine A (**1**) in CD_3OD (700 MHz) 18
Figure S10. HSQC NMR spectrum of persicamidine A (**1**) in CD_3OD (700 MHz) 19
Figure S11. HMBC NMR spectrum of persicamidine A (**1**) in CD_3OD (700 MHz) 19
Figure S12. ^{1}H NMR spectrum of persicamidine A (**1**) in DMSO-d_6 (700 MHz) 20
Figure S13. COSY NMR spectrum of persicamidine A (**1**) in DMSO-d_6 (700 MHz) 20
Figure S14. HSQC NMR spectrum of persicamidine A (**1**) in DMSO-d_6 (700 MHz) 21
Figure S15. HMBC NMR spectrum of persicamidine A (**1**) in DMSO-d_6 (700 MHz) 21
Figure S16. ^{1}H-^{15}N HMBC spectrum of persicamidine A (**1**) in DMSO-d_6 (700 MHz) 22
Figure S17. NOESY NMR spectrum of persicamidine A (**1**) in DMSO-d_6 (700 MHz) 22
Figure S18. ^{1}H NMR spectrum of persicamidine A (**1**) in $CDCl_3$ (700 MHz) 23
Figure S19. ROESY NMR spectrum of persicamidine A (**1**) in $CDCl_3$ (700 MHz) 23
Figure S20. ^{1}H NMR spectrum of persicamidine B (**2**) in CD_3OD (700 MHz) 24
Figure S21. ^{13}C NMR spectrum of persicamidine B (**2**) in CD_3OD (175 MHz) 24
Figure S22. COSY NMR spectrum of persicamidine B (**2**) in CD_3OD (700 MHz) 25
Figure S23. TOCSY NMR spectrum of persicamidine B (**2**) in CD_3OD (700 MHz) 25
Figure S24. HSQC NMR spectrum of persicamidine B (**2**) in CD_3OD (700 MHz) 26
Figure S25. HMBC NMR spectrum of persicamidine B (**2**) in CD_3OD (700 MHz) 26
Figure S26. ROESY NMR spectrum of persicamidine B (**2**) in CD_3OD (700 MHz) 27
Figure S27. NOESY NMR spectrum of persicamidine B (**2**) in CD_3OD (700 MHz) 27
Figure S28. ^{1}H NMR spectrum of persicamidine B (**2**) in DMSO-d_6 (700 MHz) 28
Figure S29. COSY NMR spectrum of persicamidine B (**2**) in DMSO-d_6 (700 MHz) 28
Figure S30. HSQC NMR spectrum of persicamidine B (**2**) in DMSO-d_6 (700 MHz) 29
Figure S31. HMBC NMR spectrum of persicamidine B (**2**) in DMSO-d_6 (700 MHz) 29
Figure S32. NOESY NMR spectrum of persicamidine B (**2**) in DMSO-d_6 (700 MHz) 30
Figure S33. ^{1}H NMR spectrum of persicamidine B (**2**) in $CDCl_3$ (700 MHz) 30
Figure S34. ROESY NMR spectrum of persicamidine B (**2**) in $CDCl_3$ (700 MHz) 31

Figure S35. ^{1}H NMR spectrum of persicamidine C (**3**) in CD_3OD (700 MHz) 31
Figure S36. ^{13}C NMR spectrum of persicamidine C (**3**) in CD_3OD (175 MHz) 32
Figure S37. COSY NMR spectrum of persicamidine C (**3**) in CD_3OD (700 MHz) 32
Figure S38. TOCSY NMR spectrum of persicamidine C (**3**) in CD_3OD (700 MHz) 33
Figure S39. HSQC NMR spectrum of persicamidine C (**3**) in CD_3OD (700 MHz) 33
Figure S40. HMBC NMR spectrum of persicamidine C (**3**) in CD_3OD (700 MHz) 34
Figure S41. ^{1}H NMR spectrum of persicamidine D (**4**) in CD_3OD (700 MHz) 34
Figure S42. ^{13}C NMR spectrum of persicamidine D (**4**) in CD_3OD (175 MHz) 35
Figure S43. COSY NMR spectrum of persicamidine D (**4**) in CD_3OD (700 MHz) 35
Figure S44. TOCSY NMR spectrum of persicamidine D (**4**) in CD_3OD (700 MHz) 36
Figure S45. HSQC NMR spectrum of persicamidine D (**4**) in CD_3OD (700 MHz) 36
Figure S46. HMBC NMR spectrum of persicamidine D (**4**) in CD_3OD (700 MHz) 37
Figure S47. ^{1}H NMR spectrum of persicamidine E (**5**) in CD_3OD (700 MHz) 37
Figure S48. ^{13}C NMR spectrum of persicamidine E (**5**) in CD_3OD (175 MHz) 38
Figure S49. COSY NMR spectrum of persicamidine E (**5**) in CD_3OD (700 MHz) 38
Figure S50. TOCSY NMR spectrum of persicamidine E (**5**) in CD_3OD (700 MHz) 39
Figure S51. HSQC NMR spectrum of persicamidine E (**5**) in CD_3OD (700 MHz) 39
Figure S52. HMBC NMR spectrum of persicamidine E (**5**) in CD_3OD (700 MHz) 40
Figure S53. ^{1}H NMR spectrum of (*S*)-Mosher ester of persicamidine A in $CDCl_3$ (700 MHz) 40
Figure S54. ^{1}H NMR spectrum of (*R*)-Mosher ester of persicamidine A in $CDCl_3$ (700 MHz) 41
Figure S55. ^{1}H NMR spectrum of (*S*)-Mosher ester of persicamidine B in $CDCl_3$ (700 MHz) 41
Figure S56. ^{1}H NMR spectrum of (*R*)-Mosher ester of persicamidine B in $CDCl_3$ (700 MHz) 42
Figure S57. ^{1}H NMR spectrum of (*S*)-Mosher ester of the cleaved glycon derivative in $CDCl_3$ (700 MHz) 42
Figure S58. ^{1}H NMR spectrum of (*R*)-Mosher ester of the cleaved glycon derivative in $CDCl_3$ (700 MHz) 43
Figure S59. ^{1}H NMR spectrum of persicamidine A aglycon formed via Payne-type rearrangement in DMSO-d_6 (700 MHz) 43
Figure S60. COSY NMR spectrum of persicamidine A aglycon formed via Payne-type rearrangement in DMSO-d_6 (500 MHz) 44
Figure S61. HSQC NMR spectrum of persicamidine A aglycon formed via Payne-type rearrangement in DMSO-d_6 (700 MHz) 44
Figure S62. HMBC NMR spectrum of persicamidine A aglycon formed via Payne-type rearrangement in DMSO-d_6 (700 MHz) 45
Figure S63. NOESY NMR spectrum of persicamidine A aglycon formed via Payne-type rearrangement in DMSO-d_6 (500 MHz) 45

List of Tables

Table S1. Concentration of media constituents of the GYM Streptomycetes medium and medium 5294 used for strain 4NS15. 5
Table S2. ^{1}H (#H, mult., *J* in Hz), ^{13}C, ^{15}N , COSY (#H → #H), and HMBC (#H → #C) NMR data of persicamidines A (**1**), B (**2**) and the aglycon of persicamidine B (**2-Aglycon**) in DMSO-d_6 (700 MHz). 10
Table S3**.** ^{1}H NMR data of persicamidines A-E (**1**-**5**) in CD_3OD (700 MHz). 12
Table S4. ^{13}C NMR data of persicamidines A-E (**1**-**5**) in CD_3OD (175 MHz). 13
Table S5. ^{1}H (700 MHz) and ^{13}C (175 MHz) NMR data of persicamidines A (**1**) and B (**2**) in $CDCl_3$. 14
Table S6. ^{1}H (700 MHz) and ^{13}C (175 MHz) NMR data (DMSO-d_6) of persicamidine A aglycon formed via Payne-type rearrangement. 15
Table S7. Mass spectrometry data deposited in MassIVE repository 16

S1. General Information and Materials

Instrumentation. Aqueous mobile-phases for semi-preparative HPLC were prepared using water purified with a Milli-Q® Integral water purification system (purified to a resistivity of 18.2 MΩ.cm at 25°C). Acetonitrile (ACN) and formic acid (FA) used were HPLC grade. Mobile-phases for analytical HPLC were prepared using water distilled over a vertical double distillation apparatus, LC-MS grade organic solvents, and LC-MS grade formic acid.
NMR spectra were recorded on a Bruker Ascend 700 spectrometer (Bruker Corporation, Billerica, Massachusetts, USA) with a 5 mm TXI cryoprobe (^{1}H at 700 MHz, ^{13}C at 175 MHz) at ambient temperature using standard pulse programs. Chemical shifts are expressed in parts per million (ppm) from CD_3OD (δ_H = 3.31, δC = 49.0), DMSO (δ_H = 2.50, δ_C = 39.52). Multiplicities are described as s (singlet), d (doublet), q (quadruplet), dd (doublet of doublets), ddd (doublet of doublets of doublets), t (triplet), dd (doublet of triplets), m (multiplet), br (broad). Coupling constants *J* are reported in Hertz (Hz) to the nearest 0.1 Hz.

Analytical LC-MS-MS was performed using a Dionex Ultimate 3000 UPLC system (Thermo Fisher Scientific Inc., Waltham, Massachusetts, USA) using a Waters BEH C18 (100 x 2.1 mm, 1.7 µm), equipped with a DAD module, and coupled to a Bruker maXis 4G UHR-TOF mass spectrometer (Bruker Daltonics) with electrospray ionization (ESI) and MS^2 capabilities. High-resolution mass spectra (HRMS) and MS^2 fragmentation spectra were recorded using the same systems as for LC-MS. The following chromatographic systems were used --- System **A**: temperature of 45°C, flow rate of 0.6 mL/min with ACN/0.1% FA and H_2O/0.1% FA [5% ACN (0.5 min), linear gradient from 5 to 95% of ACN (18 min), 95% ACN (2 min) followed by re-equilibration to the starting conditions], and UV detection in the range from 200 to 600 nm. LCMS and MS^2 fragmentation data were visualised and analysed using Bruker DataAnalysis software.

Preparative RP-HPLC was performed using a Waters Autopurifier system (Waters Corporation, Milford, Massachusetts, USA) equipped with a DAD detector module and a single-quad MS spectrometer using a waters XBridge Prep C18 column (10µm, 19 x 150 mm) at 25 mL/min flow rate, and fractions were collected automatically by time-based collection and their purity was verified by analytical LC-MS. The conditions for chromatographic system **P** used were as follows: solvent A: H_2O + 0.1% FA, solvent B: ACN + 0.1% FA; gradient: 0-5 min: 5% B, 5-16.5 min: 5-30% B, 16.5-41.5 min 30-40% B.

Semi-preparative RP-HPLC was performed using a Dionex Ultimate system (Thermo Fisher) equipped with a DAD detector, and XSelect CSH 130 Prep C18 column (5um; 10 x 250 mm) at 40°C and 5 mL/min flow rate. Compounds were collected automatically by time-based collection and their purity was verified by analytical LC-MS. The conditions for chromatographic system **SP1** used were as follows: solvent A: H_2O + 0.1% FA, solvent B: ACN + 0.1% FA; gradient: 0-3min: 5% B, 5-19 min: 5-45 % B. UV detection at 190, 220, 254, and 280 nm.

RP-HPLC fractionation of the crude extracts was performed using an Agilent 1100 HPLC system equipped with a diode-array UV detector and fraction collector using the following HPLC conditions: Dionex:XBridge (Waters) C_{18} column (100×2.1 mm, 3.5 µm); solvent A [H_2O−acetonitrile (95/5), 5 mmol NH_4Ac, 0.04 mL/L CH_3COOH]; solvent B [H_2O−acetonitrile (5/95), 5 mmol NH_4Ac, 0.04 mL/L CH_3COOH]; gradient system, 10% B increasing to 100% B in 30 min and maintaining 100% for 10 min; flow rate 0.3 mL/min; 40 °C. Fractions (0.15ml) from the HPLC column were collected in a 96-well plate every 0.5 min. The fractions in the 96-well plate were dried for 45-60 min at 40 °C using heated nitrogen. Thereafter, each well was filled with 150 µl of the tested microorganism in suitable medium.

HR-ESI-MS (High-resolution electron spray ionization mass spectrometry) spectra were also recorded for all crude extracts. On a DIONEX UltiMate system coupled to a DAD and a maXis ESI TOF mass spectrometer (Bruker Daltonics; scan range m/z 100-2500; rate 2 Hz; capillary voltage 4500 V; dry temperature 200 °C) using the following HPLC conditions: C18 Acquity UPLC BEH (Waters) column (2.1 × 50 mm, 1.7 µm); solvent A: H2O + 0.1% formic acid; solvent B: ACN + 0.1% formic acid; gradient: 5% B for 0.5 min, 5% B to 100% B in 20 min, maintaining isocratic conditions at 100% B for 10 min; flow = 0.6 mL/min; UV−vis detection 200−600 nm. Data processing and analysis of the LCMS data were done using Data Analysis software included in the Compass-software from Bruker to identify the bioactive target masses.

Marfey's LCMS analysis was performed using an Acquity BEH C18 (2.1 x 100 mm, 1.7 µm) column with a flow rate of 0.55 ml/min with the following gradient Solvent A: H2O + 0.1% FA; solvent B: ACN + 0.1% FA; gradient: 5 to 10 % B in 1 min, 10% to 35% B in 14 min, 35% to 50 % B in 7 min, and 50% to 80% B in 3 min. Detection was achieved by DAD and UHPLC maXis4G qTOF HRMS in positive mode.

S2. Experimental Procedures

Cultivation, isolation, and purification of strain 4NS15T

The actinomycetes, *Kibdelosporangium persicum* sp. nov. $(4NS15)^T$, was isolated from a neglected arid habitat in Kerman desert, Iran.[1] The strain was first pre-cultivated from agar plate for revitalization in GYM medium at 35 °C and pH of 7.2 for 7 days with gentle shaking. Thereafter, 10% inoculation was done in medium 5294 and the cultures were incubated on a rotary shaker (160 RPM) at 30 °C for 15 d. A total volume of 10 L was centrifuged, and the pellet and supernatant were separated. Then, 5% XAD was added to the supernatant and shaking was continued for additional 24 h. The XAD resin from supernatant was collected by sieving, washed by flushing with distilled water, and then extracted three times with methanol. A 843-g pellet was extracted with acetone. Acetone was added to cell pellet, sonicated, filtered, and the solvent was evaporated using a rotary evaporator. The latter was analysed by LC-MS. The extract was further fractionated using preparative HPLC system **P**, and each fraction enriched with one derivative was then purified by semi-preparative HPLC. Table S1 shows the constituents of the different media used for the maintenance and metabolite production of the 4NS15 strain.

Table S1. Concentration of media constituents of the GYM Streptomycetes medium and medium 5294 used for strain 4NS15.

**GYM/Streptomycetes Medium (Maintenance and revitalization)**		**Medium 5294 (Metabolite production)**			
Glucose	4.0 g/L	Glucose	10.0 g/L	Glycerol	10.0 g/L
Yeast extract	4.0 g/L	Yeast extract	2.0 g/L	$CaCO_3$	3.0 g/L
Malt extract	10.0 g/L	Corn steep liquor	2.5 g/L	NaCl	1.0 g/L
$CaCO_3$	2.0 g/L	Starch (soluble)	10.0 g/L		
Agar	12.0 g/L	Pepton (Marcor S)	2.0 g/L		

* The pH of both media solutions were adjusted to pH=7.2 using KOH concentrated solution

Metabolite identification

RP-HPLC fractionation coupled to HRESIMS of the extracts showed five compounds with a 14 mass unit difference in the active area of strain 4NS15. MS^2 fragmentation patterns revealed the compounds belonged to same family.
Purification of these compounds was achieved in two steps. First, the crude extract was separated using the preparative LCMS Waters system (system **P**), resulting in five fractions enriched each with one of the targeted derivatives. Further purification of each fraction using the semi-preparative HPLC Thermo system (system **SP1**) afforded the pure compounds (Fr 1, m=1.2 mg, 785.5; Fr 2, m=13.3 mg, 799.5; Fr 3, m=16.5 mg, 813.5; Fr 4, m=3.4 mg, 827.5; Fr 5, m= 1.1 mg, 841.6) as light brown solids.

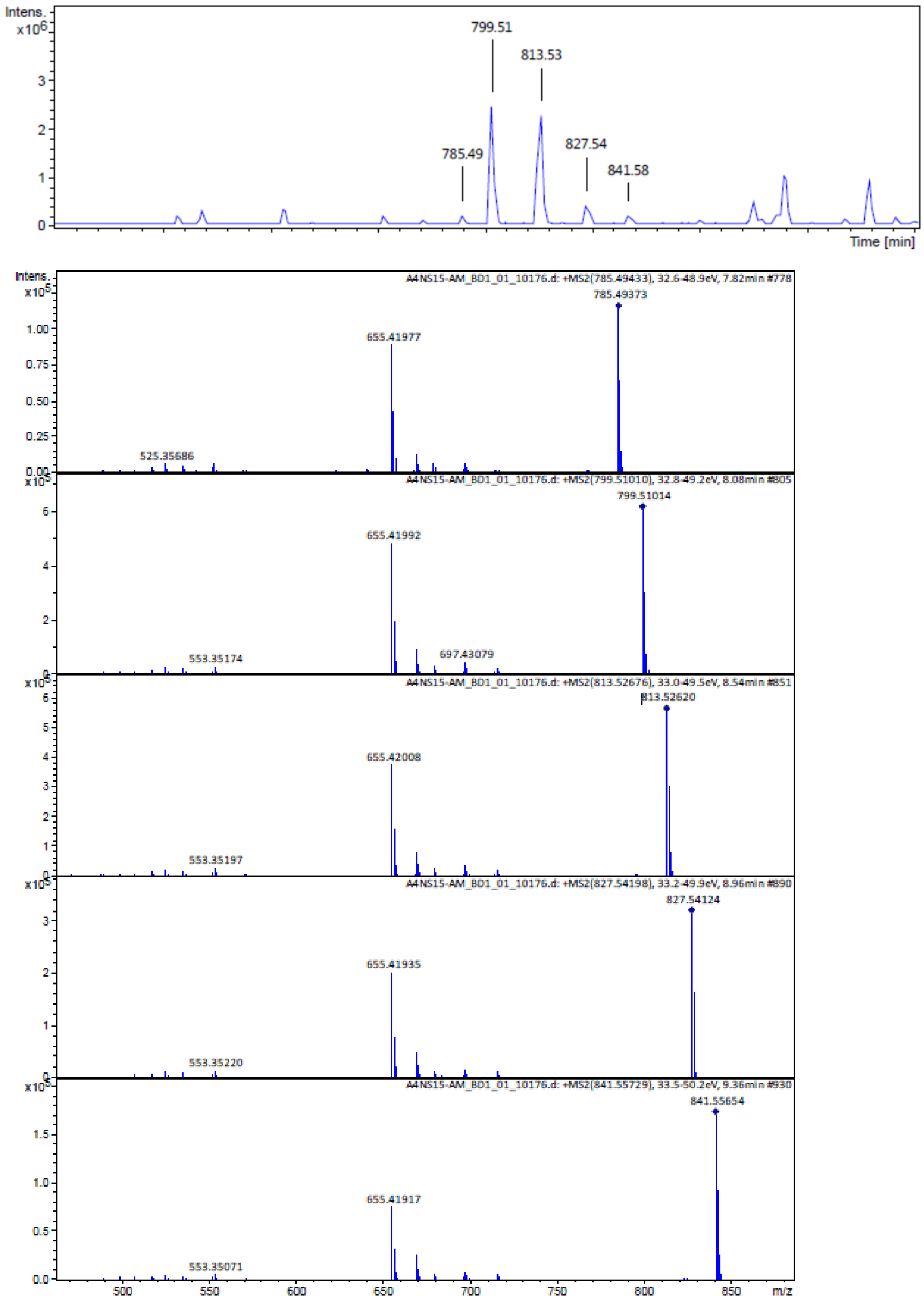

Figure S1. LCMS spectrum of the methanol extract of *Kibdelosporangium persicum* sp. nov., and MS^2 spectra of persicamidines A-E (**1**-**5**)

S3. Chemical Derivatization Methods

Analysis Using Advanced Marfey's Method

To determine the absolute configuration of the alkylamine chain, advanced Marfey's method was used.[2] Approximately 0.1 mg of persicamidine A was hydrolyzed using 6 N HCl (0.2 mL) and shaken at 90 °C for 16 h in Eppendorf tubes. Samples were dried under vacuum and dissolved in H_2O (100 µL). They were split into two 50 µL aliquots, and 1 N $NaHCO_3$ (20 µL) and 1% 1-fluoro-2,4-dinitrophenyl-5-leucine-amide (L-FDLA or D-FDLA solution in acetone, 100 µL) were added individually. The mixtures were heated to 40 °C for 40 min and cooled down to RT, and the reaction was quenched by adding 2 N HCl (10 µL). After adding 300 µL of ACN, the samples were centrifuged and subsequently analyzed by LCMS (S1). The standards (*S*)- and (*R*)-2-aminobutane were treated the

same way. The retention times were as followed: Persicamidine A with D-FDLA: 11.32 min; Persicamidine A with L-FDLA: 11.20 min; (*R*)-2-aminobutane with D-FDLA: 11.32 min; (*R*)-2-aminobutane with L-FDLA: 11.21 min; (*S*)-2-aminobutane with D-FDLA: 11.21 min; (*S*)-2-aminobutane with L-FDLA: 11.32 min.

Mosher esterification of persicamidines A and B

Persicamidines A and B (0.5 mg each) were separately dissolved in 100 µL $CDCl_3$ in 1.5-ml glass vials. To each vial were added 15 µL pyridine-*d*$_5$ and 15 µL (*R*)-(−)-α-methoxy-α-(trifluoromethyl)phenylacetyl chloride (*R*-MTPACl), and the reaction mixtures were stirred overnight to afford the corresponding triacylated (*S*)-Mosher esters.[3] Thereafter, 400 µL $CDCl_3$ was added to acquire 1D and 2D NMR data. Identical procedure was employed for esterification using *S*-MTPACl to generate the corresponding triacylated (*R*)-Mosher esters. The differences in the chemical shifts [$\Delta\delta^{SR}$ (=δ^S − δ^R)] of the Mosher esters of persicamidines A and B are shown in Figures S3 and S4.

Relevant ^{1}H NMR chemical shifts of (*S*)-Mosher ester of persicamidine A (700 MHz). 6.08 (s, H-13), 5.58 (br s, H-16), 4.75 (t, 9.3, H-4'), 4.54 (dd, 9.9, 1.6, H-1'), 4.33 (d, 11.2, H_{α}-29), 4.11 (d, 11.2, H_{β}-29), 3.33 (embedded, H-5'), 3.23 (ddd, 11.8, 9.3, 5.0, H-3'), 3.16 (d, 9.2, H-2), 3.08 (OC$\underline{H_3}$-7'), 2.71 (dd, 14.5, 11.0, H_{α}-9), 2.38 (d, 14.5, H_{β}-9), 2.32 (dd, 12.6, 5.0, H_{α}-2'), 2.22 (t, 10.8, H-8), 1.58 (m, H-19), 1.54 (m, H_{β}-2'), 1.25 (s, H_3-33), 1.23 (br s, H_3-34), 1.20 (d, 6.6, H_3-2''), 1.14 (d, 6.0, H_3-6'), 0.89 (t, 7.5, H_3-4''), 0.75 (s, H_3-32). *m/z* 1461.6468 [$C_{76}H_{94}F_9N_2O_{16}$ $(M+H)^+$].

Relevant ^{1}H NMR chemical shifts of (*R*)-Mosher ester of persicamidine A (700 MHz). 6.08 (s, H-13), 5.63 (br s, H-16), 4.79 (t, 9.5, H-4'), 4.48 (dd, 10.0, 1.7, H-1'), 4.46 (d, 11.1, H_{α}-29), 3.97 (d, 11.1, H_{β}-29), 3.33 (embedded, H-3'), 3.26 (OC$\underline{H_3}$-7'), 3.21 (dq, 9.5, 6.2, H-5'), 3.16 (d, 8.7, H-2), 2.74 (dd, 14.4, 10.7, H_{α}-9), 2.41 (d, 14.4, H_{β}-9), 2.38 (ddd, 12.6, 4.9, 1.7, H_{α}-2'), 2.24 (t, 9.8, H-8), 1.65 (m, H-19), 1.55 (m, H_{β}-2'), 1.37 (s, H_3-33), 1.20 (d, 6.6, H_3-2''), 1.11 (br s, H_3-34), 1.03 (d, 6.2, H_3-6'), 0.89 (t, 7.5, H_3-4''), 0.78 (s, H_3-32). *m/z* 1461.6513 [$C_{76}H_{94}F_9N_2O_{16}$ $(M+H)^+$].

Relevant ^{1}H NMR chemical shifts of (*S*)-Mosher ester of persicamidine B (700 MHz). 6.09 (s, H-13), 5.61 (br s, H-16), 4.76 (t, 9.4, H-4'), 4.55 (dd, 10.0, 1.8, H-1'), 4.35 (d, 10.9, H_{α}-29), 4.12 (d, 10.9, H_{β}-29), 3.33 (embedded, H-5'), 3.23 (ddd, 11.8, 9.4, 5.1, H-3'), 3.17 (d, 8.8, H-2), 3.08 (OC$\underline{H_3}$-7'), 2.71 (dd, 14.4, 11.1, H_{α}-9), 2.39 (d, 14.4, H_{β}-9), 2.33 (m, H_{α}-2'), 2.19 (t, 10.5, H-8), 1.57 (m, H_{β}-2'), 1.56 (m, H-19), 1.26 (s, H_3-33), 1.25 (d, 6.7, H_3-2''), 1.24 (br s, H_3-34), 1.23 (d, 6.7, H_3-3''), 1.14 (d, 6.3, H_3-6'), 0.76 (s, H_3-32).

Relevant ^{1}H NMR chemical shifts of (*R*)-Mosher ester of persicamidine B (700 MHz). 6.09 (s, H-13), 5.65 (br s, H-16), 4.79 (t, 9.4, H-4'), 4.48 (m, H-1'), 4.47 (d, 11.2, H_{α}-29), 3.97 (d, 11.2, H_{β}-29), 3.33 (embedded, H-3'), 3.25 (s, OC$\underline{H_3}$-7'), 3.21 (dq, 9.4, 6.1, H-5'), 3.17 (d, 8.9, H-2), 2.73 (dd, 14.2, 10.1, H_{α}-9), 2.41 (d, 14.2, H_{β}-9), 2.37 (dd, 12.8, 4.7, H_{α}-2'), 2.23 (t, 10.1, H-8), 1.66 (m, H-19), 1.53 (m, H_{β}-2'), 1.38 (s, H_3-33), 1.24 (d, 6.1, H_3-2''), 1.22 (d, 6.1 H_3-3''), 1.11 (br s, H_3-34), 1.04 (d, 6.2, H_3-6'), 0.79 (s, H_3-32). *m/z* 1447.6294 [$C_{75}H_{94}F_9N_2O_{16}$ $(M+H)^+$].

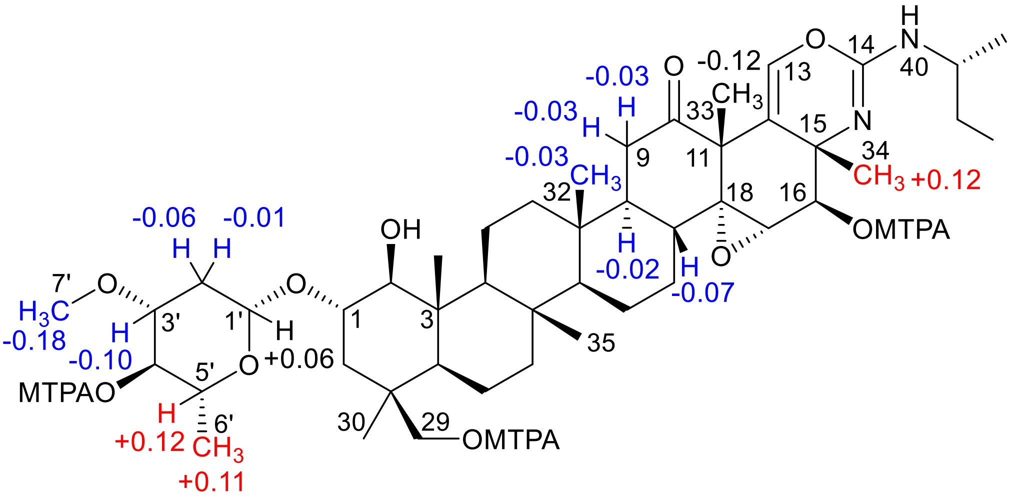

Figure S2. Differences in chemical shifts ($\Delta\delta^{SR}$ (=δ^S − δ^R)) of Mosher esters of persicamidine A

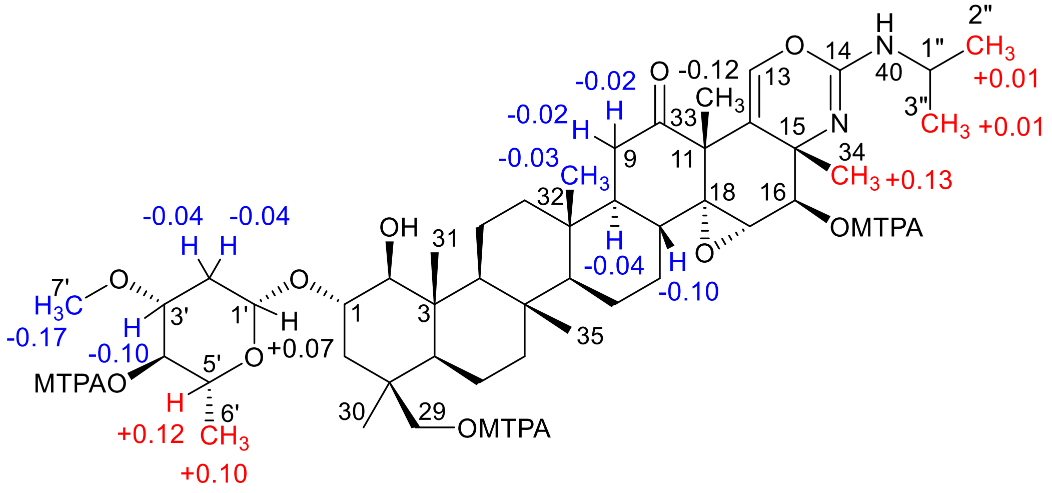

Figure S3. Differences in chemical shifts ($\Delta\delta^{SR}$ ($=\delta^S - \delta^R$)) of Mosher esters of persicamidine B

Persicamidine B was also subjected to Mosher derivatization using a slightly different procedure. After dissolving 0.75 mg of this compound in 100 µL $CDCl_3$ in a 1.5-ml glass vial, 30 µL pyridine-*d5* and 30 µL *R*-MTPACl were added. The reaction mixture was stirred for 48 h and 400 µL $CDCl_3$ was added to acquire the NMR data. Identical procedure was employed for esterification using *S*-MTPACl. Under these conditions, a mixture of compounds was obtained. Attempts to separate the acylated persciamidine B using RP-HPLC were unsuccessful, but a derivatized glycon part that cleaved from the molecule could be isolated. This molecule was characterized using 1D and 2D NMR data. The differences in the chemical shifts [$\Delta\delta^{SR}$ ($=\delta^S - \delta^R$)] of the Mosher esters of this molecule are shown in Figure S4. This data also independently supported the *R* assignment for the stereocenter at position 4', thereby eliminating any possible interference from the phenyl groups of C-29 MTPA moiety on the chemical shifts of the glycon part of the acylated persicamidines.

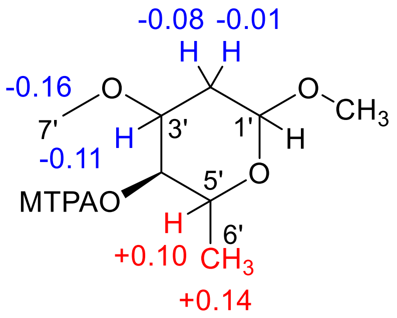

Figure S4. Differences in chemical shifts ($\Delta\delta^{SR}$ ($=\delta^S - \delta^R$)) of the Mosher esters of the cleaved glycon derivative

Relevant ^{1}H NMR chemical shifts of (*S*)-Mosher ester of the cleaved glycon derivative (700 MHz). 4.86 (t, 9.5, H-4'), 4.80 (br d, 3.3, H-1'), 3.84 (dq, 9.5, 6.2, H-5'), 3.60 (ddd, 11.3, 9.5, 5.1, H-3'), 3.33 (s, OCH_3-1'), 3.16 (s, $OC\underline{H_3}$-7'), 2.32 (dd, 12.7, 5.1, H_α-2'), 1.63 (embedded, H_β-2'), 1.25 (d, 6.2, H_3-6'). *m/z* 415.13499 [$C_{18}H_{23}F_3O_6Na$ $(M+Na)^+$], 393.15263 [$C_{18}H_{24}F_3O_6$ $(M+H)^+$].

Relevant ^{1}H NMR chemical shifts of (*R*)-Mosher ester of the cleaved glycon derivative (700 MHz). 4.89 (t, 9.4, H-4'), 4.81 (br d, 3.2, H-1'), 3.74 (dq, 9.4, 6.4, H-5'), 3.71 (ddd, 11.3, 9.4, 4.9, H-3'), 3.32 (s, $OC\underline{H_3}$-7'), 3.31 (s, OCH_3-1'), 2.38 (dd, 12.9, 4.9, H_α-2'), 1.64 (ddd, 12.9, 11.3, 3.2, H_β-2'), 1.11 (d, 6.4, H_3-6'). *m/z* 415.13368 [$C_{18}H_{23}F_3O_6Na$ $(M+Na)^+$], 393.15155 [$C_{18}H_{24}F_3O_6$ $(M+H)^+$].

Acidic hydrolysis of persicamidine A

Persicamidine A (1.7 mg) was subjected to acidic hydrolysis using 1 N HCl at 100 °C for 1.5 h. This resulted in the cleavage of sugar and formation of an aglycon that showed Payne-type rearrangement (*m/z* 669.44929 [$C_{39}H_{61}N_2O_7$ $(M+H)^+$]; Figure S5). The aglycon was purified using RP HPLC and a complete set of 2D NMR data were collected and analyzed (Table S6; Figures S59-S63).

Under acidic conditions, OH-16 (nucleophile) attacks the more substituted carbon C-18 (with inversion of configuration at this center) to open the epoxide and generate a hydroxyl group at position 17, whose configuration should be retained and similar to that of the epoxide.[4] This rearrangement is consistent with *anti*-orientation of the epoxide with respect to OH-16 in persicamidines. Such a rearrangement was previously employed by Birman and Danishefsky (2002) in the final step of merrilactone A total synthesis.[4] Attempts to confirm the absolute configuration of OH-17 using Mosher's esterification method were unsuccessful as the reaction only yielded a diacylated product, where OH-17 did not react. However, the observed NOESY correlations (H17↔H19, H17↔H-16, H-17↔H-8 (wk), H-16↔H_3-34) as well as $^3J_{H\text{-}16/H\text{-}17}$ value (5.6 Hz; H-16-C16-C17-H17 dihedral angle of ~30°; calculation of equilibrium geometry in polar solvent via DFT calculation (B3LYP, 6-31G*) using Spartan software) were consistent with the conformation adopted by this molecule with (*S*)-configuration of C-17. For 17-(*R*) configuration, a smaller coupling constant of 0-2 Hz was expected (H-16-C16-C17-H17 dihedral angle of ~108°).

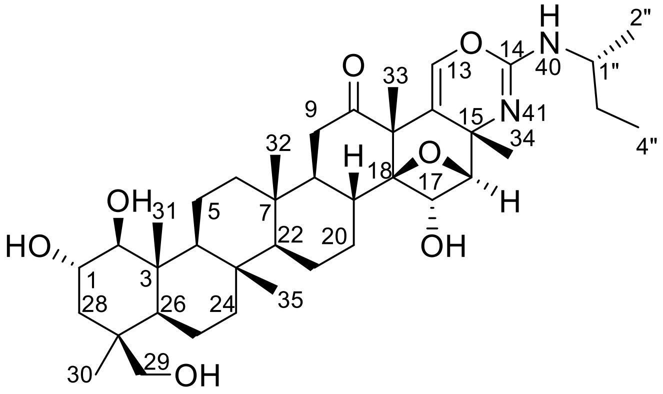

Figure S5. Persicamidine A aglycon formed via Payne-type rearrangement

S4. Biological evaluation of persicamidines A-E

hCoV-229E virus infection assay

Renilla luciferase (RLuc) expressing alphacoronavirus hCoV-229E[5] (stock kindly provided by Volker Thiel, Institute of Virology and Immunology, Bern and Mittelhaeusern, Switzerland and Department of Infectious Diseases and Pathobiology, Vetsuisse Faculty, University of Bern, Switzerland) was propagated in Huh-7.5 cells (kindly provided by Prof. Charles M. Rice, Laboratory of Virology and Infectious Diseases, The Rockefeller University, New York, NY USA) at 33 °C and harvested when cytopathic effects were visible. Firefly luciferase (FLuc) expressing Huh-7.5 cells were seeded in a density of 2 x 10^4 cells/well in a 96-well plate in DMEM supplemented with 10% FCS, 100 U/mL penicillin, 100 U/mL streptomycin, and 1% non-essential amino acids (DMEM complete). The next day, cells were inoculated with hCoV-229E virus (MOI 0.05) and treated simultaneously with given concentrations of compounds or DMSO (1% final DMSO concentration). Remdesivir was used as a positive control. After 48 hpi, cells were lysed with 50 µL PBS containing 0.5% Triton X-100 and frozen at -20 °C. RLuc and FLuc activities were determined as a measure of residual infectivity and cell viability, respectively. Mean values of two to five biological replicates (due to varied concentration ranges) containing duplicates and standard deviations are depicted. Regression curves were calculated with GraphPad Prism 9 and used to interpolate half-maximal inhibitory (IC_{50}) and cytotoxic (CC_{50}) concentrations.

SARS-CoV-2 infection assay

A549 cells overexpressing the SARS-CoV-2 cellular receptor ACE2 and serine protease TMPRSS2 (A549-TA were a kind gift from Dr. Marek Widera, Frankfurt)[6] were cultured in Dulbecco's MEM with 10% FCS and 2 mM glutamine. Cells were seeded in 96-well plates at a density of 2 x 10^5 cells/well at 37 °C (5% CO_2). The next day, cells were treated in triplicates with live/dead stain IncuCyte® CytoLight Rapid Red Reagent (Sartorius, Goettingen, Germany) and infected with the icSARS-CoV-2-mNeonGreen reporter virus [kind gift from Dr. Pei Yong Shi, University of Texas, Medical Branch, USA (UTMB) and was provided via the World Reference Center for Emerging Viruses and Arboviruses (WRCEVA, Texas, USA)] in the presence of the respective compounds in indicated concentrations.[7] Infected cells were further incubated in an IncuCyte® S3 Live-Cell Analysis Instrument (Sartorius, Goettingen, Germany) and fluorescence monitored after 24 hpi. As an antiviral compound control, Remdesivir was used (full viral inhibition at 1 µM). Average values of triplicates were normalized against infected but untreated cells. For determination of cell viability, puromycin controls (1 µM) were set as 100% cytotoxic. Mean values of three biological replicates were normalized against the puromycin control. Overall average values of three biological replicates and standard deviations are depicted. Regression curves were fitted with GraphPad Prism 9 and used to determine IC_{50} and CC_{50} concentrations.

S5. NMR data

All 1D and 2D raw NMR data have been submitted to the NP-MRD database. The submission will be live at [***https://npmrd-project.org/***](https://npmrd-project.org/) after publication of this manuscript.

NMR tables

Table S2. ^{1}H (#H, mult., *J* in Hz), ^{13}C, ^{15}N , COSY (#H → #H), and HMBC (#H → #C) NMR data of persicamidines A (**1**), B (**2**) and the aglycon of persicamidine B (**2-Aglycon**) in DMSO-d_6 (700 MHz).

	**1**					**2**		**2-Aglycon**	
#	δ_H	δ_C	δ_N	**#H → #H**	**#H → #C**	δ_H	δ_C	δ_H	δ_C
1	3.41, 1H, m	78.1		2, 28a, 28b	28, 2, 1'	3.41, 1H, m	78.1	3.35	67.4
2	2.95, 1H, br d, 8.6	82.4		1	31, 28, 3, 4, 1	2.95, 1H, br d, 8.7	82.4	2.84	84.3
3		43.0					43.1		43.0
4	0.93, 1H, m	61.7		5a, 5b	31, 35, 23, 3	0.93, 1H, m	61.8	0.91	61.8
5a	2.57, 1H, br d, 13.0	19.1		4, 5b, 6	4	2.57, 1H, br d, 13.7	19.1	2.57	19.2
5b	1.31, 1H, m			4, 6, 5a		1.31, 1H, m		1.30	
6a	1.70, 1H, br d, 12.7	43.8		6, 5a, 5b	4	1.70, 1H, br d, 13.2	43.9	1.70	43.8
6b	0.94, 1H, m			6, 5a, 5b		0.93, 1H, m		0.92	
7		36.3					36.5		36.3
8	2.12, 1H, br t, 9.0	47.0		19, 9a, 9b	32, 20, 7, 19, 6, 10	2.12, 1H, br t, 10.0	47.1	2.12	47.1
9a	2.69, 1H, dd, 14.0, 9.0	37.9		8, 9b	7, 8, 10	2.69, 1H, dd, 13.9, 10.0	38.0	2.69	37.9
9b	2.19, 1H, d, 14.0			8, 9a	7, 19, 8, 11, 10	2.19, 1H, d, 13.9		2.19	
10		213.7					213.7		213.7
11		49.9					49.9		49.7
12		125.8					125.8		125.8
13	5.72, 1H, s	131.2		16[w]	33, 34, 11, 15, 18, 16, 12,	5.73, 1H, s	131.2	5.71	131.2
14		149.4					149.4		149.0
15		53.3					53.4		53.4
16	4.07, 1H, br d, 3.5	73.1		17, 13[w]	15, 17, 18, 12, 13, 14	4.08, 1H, d, 3.7	73.1	4.08	73.2
17	3.21, 1H, d, 3.5	59.1		16	19, 15, 18, 16, 10	3.22, 1H, m	59.0	3.21	59.0
18		64.5					64.6		64.3
19	1.57, 1H, m	39.1		20a, 20b, 8	20, 8, 11, 18	1.57, 1H, m	39.2	1.57	39.1
20a	1.58, 1H, m	22.4		20b, 21	19, 18	1.58, 1H, m	22.3	1.58	22.3
20b	1.45, 1H, m			19, 20a	21, 19, 18	1.45, 1H, m		1.45	
21	1.43, 2H, m	19.3		22, 20a		1.43, 2H, m	19.3	1.42	19.3
22	1.03, 1H, m	51.4		21	23	1.02, 1H, m	51.4	1.02	51.4
23		38.2					38.4		38.2
24a	1.53, 1H, br d, 12.6	40.9		24b, 25		1.53, 1H, br d, 12.0	41.0	1.53	41.0
24b	0.82, 1H, m			25, 24a	35	0.82, 1H, m		0.79	
25	1.44, 2H, m	17.5		24a, 24b,	24	1.45, 2H, m	17.6	1.43	17.4
26	0.86, 1H, m	54.7		25, 28[w]	31, 25, 30, 27, 3, 1	0.86, 1H, m	54.7	0.87	55.2
27		38.8					38.9		39.2
28a	2.07, 1H, br dd, 12.7, 4.1	41.0		1, 28b	25, 30, 27, 26, 29, 1, 2	2.07, 2H, dd, 13.3, 4.8	41.0	1.92	41.7
28b	0.82, 1H, m			1, 28a		0.82, 1H, m		0.80	
29a	3.35, 1H, d, 10.4	63.1			30, 27, 28, 26	3.35, 1H, d, 10.4	63.0	3.40	62.8
29b	3.21, 1H, d, 10.4				30, 27, 28	3.21, 1H, m		3.15	
30	0.83, 3H, s	27.2			26, 28, 26, 29, 1	0.83, 3H, s	27.3	0.83	27.2
31	0.83, 3H, s	14.2			26, 4, 2	0.83, 3H, s	14.2	0.80	14.3
32	0.76, 3H, s	17.8			7, 6, 8, 22	0.76, 3H, s	17.8	0.75	17.7
33	1.44, 3H, s	21.3			11, 18, 12, 13, 10	1.44, 3H, s	21.2	1.44	21.3

34	0.99, 3H, s	26.3			15, 16, 12	0.99, 3H, s	26.2	0.98	26.3
35	0.82, 3H, s	15.9			23, 24, 22	0.82, 3H, s	15.8	0.81	15.9
40	5.90, 1H, br s		85.7	1''					
41			181.0						
1'	4.56, 1H, br d, 9.5	100.5		2'a, 2'b	2', 5', 1, 3'	4.56, 1H, dd, 9.7, 1.5	100.5		
2'a	2.38, 1H, br dd, 11.2, 4.6	35.8		2'b, 3', 1'	4', 3', 1'	2.37, 1H, ddd, 12.0, 4.9, 1.5	35.8		
2'b	1.10, 1H, m			2'a, 3', 1'	3', 1'	1.10, 1H, m			
3'	3.04, 1H, m	80.0		2'a, 2'b, 4'	7', 4'	3.04, 1H, m	80.0		
4'	2.79, 1H, br t, 8.9	74.9		3', 5'	6', 5', 3'	2.79, 1H, t, 8.9	75.0		
5'	3.10, 1H, dq, 9.1, 6.2	71.3		6', 4'	6', 4', 3', 1'	3.10, 1H, dq, 8.9, 6.2	71.4		
6'	1.14, 3H, d, 6.2	18.0		5'	5', 4', 1'	1.14, 3H, d, 6.2	17.9		
7'	3.30, 3H, s	56.1			3'	3.30, 3H, s	56.1		
1''	3.32, 1H, m	48.3		2'', 3'', 3'', 40	4'', 2'', 3'', 14	3.53, 1H, sept, 6.5	42.7	3.51	42.6
2''	1.00, 3H, d, 6.5	19.5		1''	3'', 1''	1.02, 3H, d, 6.5	22.0	1.02	21.8
3''a	1.47, 1H, m	28.3		1'', 3''b	4'', 2'', 1''	1.03, 3H, d, 6.5	22.0	1.03	21.8
3''b	1.28, 1H, m			4'', 1'', 3''a	4'', 2'', 1'', 14				
4''	0.82, 3H, t, 7.4	10.4		3''	3'', 1''				
				[w] weak long-range 5*J* COSY correlations are observed					

Table S3. ^{1}H NMR data of persicamidines A-E (**1**-**5**) in CD_3OD (700 MHz).

#	**1**	**2**	**3**	**4**	**5**
1	3.63-3.55*, 1H, m	3.61-3.55*, 1H, ddd, 12.0, 9.1, 4.9	3.61-3.54*, 1H, m	3.62-3.54*, 1H, m	3.60-3.55*, 1H, m
2	3.13, 1H, d, 8.9	3.13, 1H, d, 8.9	3.13, 1H, d, 8.9	3.13, 1H, d, 9.0	3.13, 1H, d, 8.9
4	1.07-1.01*, 1H, m	1.05*, 1H, dd, 12.3, 1.8	1.09-1.02*, 1H, m	1.08-1.02*, 1H, m	1.08-1.03*, 1H, m
5a	2.60, 1H, br dt, 13.6, 3.8	2.60, 1H, br dt, 12.8, 2.8	2.59, 1H, br d, 13.2	2.59, 1H, br d, 12.8	2.59, 1H, br d, 12.4
5b	1.53-1.45*, 1H, m	1.51-1.44*, 1H, m	1.51-1.44*, 1H, m	1.52-1.45*, 1H, m	1.53-1.44*, 1H, m
6a	1.84, 1H, dt, 13.0, 3.2	1.84, 1H, dt, 12.9, 3.2	1.83, 1H, dt, 13.0, 3.1	1.83, 1H, dt, 12.5, 2.9	1.83, 1H, dt, 13.0, 3.2
6b	1.09-1.05*, 1H, m	1.06*, 1H, td, 13.4, 4.3	1.09-1.02*, 1H, m	1.08-1.02*, 1H, m	1.08-1.03*, 1H, m
8	2.31-2.24*, 1H, m	2.28, 1H, t, 10.0	2.28, 1H, t, 10.4	2.27*, 1H, t, 9.9	2.27*, 1H, t, 10.1
9a	2.81, 1H, dd, 14.0, 11.0	2.80, 1H, dd, 14.1, 11.0	2.81, 1H, dd, 14.0, 11.02	2.81, 1H, dd, 14.1, 11.1	2.80, 1H, dd, 14.0, 11.1
9b	2.40, 1H, br d, 14.1	2.42, 1H, br d, 14.2	2.40, 1H, d, 14.1	2.37, 1H, d, 14.1	2.37, 1H, br d, 14.0
13	6.14, 1H, s	6.19, 1H, s	6.13, 1H, s	6.05, 1H, s	6.04, 1H, s
16	4.31, 1H, br d, 1.6	4.33, 1H, br s	4.31, 1H, br s	4.27, 1H, br s	4.31, 1H, br d, 2.9
17	3.42-3.39*, 1H	3.43-3.41*, 1H	3.39*, 1H, d, 3.6	3.37, 1H, d, 3.4	3.38, 1H, d, 3.8
19	1.78-1.71*, 1H, m	1.78-1.71*, 1H, m	1.78-1.69*, 1H, m	1.75-1.70*, 1H, m	1.75-1.70*, 1H, m
20a	1.78-1.71*, 1H, m	1.78-1.71*, 1H, m	1.78-1.69*, 1H, m	1.75-1.70*, 1H, m	1.75-1.70*, 1H, m
20b	1.64-1.59*, 1H, m	1.65-1.60*, 1H, m	1.64-1.59*, 1H, m	1.64-1.59*, 1H, m	1.64-1.59*, 1H, m
21	1.59-1.52*, 2H, m	1.59-1.52*, 2H, m	1.60-1.50*, 2H, m	1.60-1.50*, 2H, m	1.59-1.52*, 2H, m
22	1.13, 1H, t, 9.4	1.13, 1H, dd, 10.0, 9.0	1.12, 1H, dd, 9.6, 9.6	1.15-1.10*, 1H	1.12, 1H, br t, 9.5
24a	1.67, 1H, dt, 12.6, 2.6	1.67, 1H, dt, 12.8, 3.2	1.67, 1H, dt, 12.9, 2.9	1.66, 1H, dt, 12.4, 2.6	1.66, 1H, dt, 12.7, 2.9
24b	0.92-0.88*, 1H, m	0.92-0.88*, 1H, m	0.99-0.88*, 1H, m	0.92-0.88*, 1H, m	0.92-0.88*, 1H, m
25a	1.63-1.52*, 2 H, m	1.62-1.57*, 1H, m	1.62-1.55*, 1H, m	1.62-1.57*, 1H, m	1.63-1.56*, 1H, m
25b		1.55-1.50*, 1H, m	1.55-1.49*, 1H, m	1.56-1.49*, 1H, m	1.55-1.50*, 1H, m
26	0.99*, 1H, dd, 12.3, 1.6	0.99*, 1H, dd, 12.2, 2.1	0.99*, 1H, dd, 12.0, 1.6	0.99*, 1H, dd, 12.2, 2.0	0.99*, 1H, dd, 11.9, 1.8
28a	2.24, 1H, dd, 13.4, 4.7	2.25, 1H, dd, 13.3, 4.7	2.24, 1H, dd, 13.4, 4.8	2.24, 1H, dd, 13.4, 4.9	2.24, 1H, dd, 13.3, 4.7
28b	1.02*, 1H, t, 12.6	1.02*, 1H, t, 12.9	1.02*, 1H, t, 12.3	1.02*, 1H, t, 12.2	1.02*, 1H, t, 11.9
29a	3.59, 1H, d, 11.0	3.59, 1H, d, 11.3	3.59, 1H, d, 11.3	3.59, 1H, d, 11.1	3.59, 1H, d, 11.2
29b	3.39*, 1H, d, 11.4	3.40*, 1H, d, 11.1	3.39*, 1H, d, 11.1	3.39, 1H, d, 11.3	3.39, 1H, d, 11.4
30	0.95, 3H, s	0.95, 3H, s	0.95, 3H, s	0.95, 3H, s	0.95, 3H, s
31	0.96, 3H, s	0.96, 3H, s	0.96, 3H, s	0.96, 3H, s	0.96, 3H, s
32	0.88, 3H, s	0.88, 3H, s	0.87, 3H, s	0.87, 3H, s	0.87, 3H, s
33	1.59, 3H, s	1.60, 3H, s	1.59, 3H, s	1.58, 3H, s	1.59, 3H, s
34	1.24, 3H, s	1.34, 3H, s	1.30, 3H, s	1.24, 3H, s	1.24, 3H, s
35	0.93, 3H, s	0.93, 3H, s	0.93, 3H, s	0.93*, 3H, s	0.93, 3H, s
1'	4.68, 1H, dd, 9.7, 1.8	4.68, 1H, dd, 9.7, 1.8	4.68, 1H, dd, 9.7, 1.6	4.68, 1H, dd, 9.8, 1.9	4.68, 1H, dd, 9.8, 1.8
2'a	2.44, 1H, ddd, 12.4, 5.0, 1.8	2.44, 1H, ddd, 12.5, 5.1, 1.9	2.44, 1H, ddd, 12.4, 4.9, 1.6	2.44, 1H, ddd, 12.4, 5.0, 1.8	2.44, 1H, ddd, 12.4, 5.0, 1.8
2'b	1.34-1.29*, 1H, m	1.31, 1H, ddd, 12.0, 12.0, 10.0	1.34-1.28, 1H, m	1.35-1.29*, 1H, m	1.30*, 1H, ddd, 11.9, 11.9, 9.9
3'	3.18, 1H, ddd, 11.5, 8.7, 4.9	3.17, 1H, ddd, 11.6, 8.7, 5.0	3.17, 1H, ddd, 11.6, 8.8, 5.0	3.17, 1H, ddd, 11.6, 8.8, 5.0	3.17, 1H, ddd, 11.6, 8.8, 5.0
4'	2.96, 1H, dd, 9.0, 9.0	2.96, 1H, dd, 9.0, 9.0	2.96, 1H, dd, 9.0, 9.0	2.96, 1H, dd, 9.0, 9.0	2.96, 1H, dd, 9.0, 9.0
5'	3.26, 1H, dq, 9.2, 6.1	3.26, 1H, dq, 9.2, 6.1	3.26, 1H, dq, 9.2, 6.2	3.26, 1H, dq, 9.3, 6.1	3.26*, 1H, dq, 9.3, 6.2
6'	1.28, 3H, d, 6.2	1.27, 3H, d, 6.2	1.27, 3H, d, 6.2	1.27, 3H, d, 6.3	1.27, 3H, d, 6.2
7'	3.42, 3H, s	3.42, 3H, s	3.42, 3H, s	3.42, 3H, s	3.42, 3H, s
1''	3.73-3.65, 1H, m	3.95-3.86, 1H, m	3.61-3.54*, 1H, m	3.62-3.54*, 1H, m	3.28-3.20*, 2H, m
2''	1.15, 3H, d, 6.5	1.24, 3H, d, 6.6	1.15, 3H, d, 6.6	1.11, 3H, d, 6.8	1.17, 3H, t, 7.2
3''	1.55*, 2H, br t, 7.2	1.23, 3H, d, 6.5	1.78-1.69*, 1H, m	-	-
4''/5''/6''	0.94, 3H, d, 7.3	-	0.95, 3H, d, 5.8; 0.94, 3H, d, 6.6	0.93*, 9H, s	-

*Overlapped/embedded signals

Table S4. ^{13}C NMR data of persicamidines A-E (**1**-**5**) in CD_3OD (175 MHz).

#	1	2	3	4	5
1	80.2	80.2	80.2	80.2	80.3
2	84.8	84.8	84.8	84.8	84.8
3	44.9	44.9	44.9	44.9	44.9
4	63.9	63.9	63.9	64	63.9
5	21.1	21.1	21.1	21.1	21.1
6	45.7	45.7	45.7	45.7	45.7
7	38.3	38.2	38.3	38.3	38.3
8	48.9	49.0	49.0	49.0	49.0
9	39.4	39.4	39.4	39.4	39.4
10	214.7	214.3	214.8	215.4	215.1
11	51.3	51.3	51.3	51.4	51.4
12	128.9	129	128.8	128.5	128.6
13	133.9	133.9	133.9	133.8	133.8
14	156.8	156.8	156.9	156.3	156.3
15	56.0	56.1	56.0	56.0	56.1
16	71.1	70.5	71.2	72.3	72
17	60.5	60.4	60.5	60.7	60.7
18	66.7	66.7	66.6	66.6	66.5
19	41.1	41.0	41.1	41.2	41.2
20	24.0	24.0	24.0	24.0	24.0
21	21.0	21.0	21.0	21.0	21.0
22	53.6	53.6	53.6	53.6	53.6
23	40.1	40.1	40.1	40.1	40.1
24	42.8	42.8	42.8	42.8	42.8
25	19.2	19.2	19.2	19.2	19.2
26	56.9	56.9	56.9	56.9	56.9
27	40.6	40.6	40.6	40.6	40.6
28	42.2	42.2	42.2	42.2	42.2
29	65.5	65.5	65.5	65.5	65.5
30	27.8	27.8	27.8	27.8	27.7
31	15.1	15.1	15.1	15.1	15.1
32	18.9	18.9	18.9	18.9	18.9
33	22.0	21.9	22.0	22.1	22.1
34	26.3	26.3	26.3	26.5	26.3
35	17.0	16.9	16.9	16.9	16.9
1'	102.4	102.4	102.5	102.5	102.4
2'	37.2	37.2	37.2	37.2	37.2
3'	81.9	81.8	81.8	81.9	81.9
4'	77.0	77.0	77.0	77.0	77.0
5'	73.3	73.3	73.3	73.3	73.3
6'	18.4	18.4	18.4	18.4	18.4
7'	57.4	57.4	57.3	57.4	57.3
1''	51.2	45.9	55.1	57.7	37.8
2''	20.4	22.6	17.6	16.1	14.6
3''	30.3	22.5	34.4	35.5	-
4''/5''/6''	10.7	22.5	19.2/18.8	26.4	-

Table S5. ^{1}H (700 MHz) and ^{13}C (175 MHz) NMR data of persicamidines A (**1**) and B (**2**) in $CDCl_3$.

#	**1**		**2**	
	δ_H	δ_C	δ_H	δ_C[a]
1	3.61, 1H, m	78.9	3.60, 1H, ddd, 12.8, 9.0, 4.7	78.8
2	3.20, 1H, br d, 9.2	82.7	3.20, 1H, br d, 9.0	82.7
3		43.6		43.6
4	1.01, 1H, m	62.4	1.01, 1H, m	62.5
5a	2.54, 1H, br d, 13.7	19.9	2.55, 1H, br d, 14.2	19.9
5b	1.51, 1H, m		1.50, 1H, m	
6a	1.82, 1H, m	44.4	1.82, 1H, dt, 13.1, 3.4	44.3
6b	1.02, 1H, m		1.02, 1H, m	
7		37.0		37.0
8	2.29, 1H, m	47.4	2.28, 1H, m	47.0
9a	2.77, 1H, dd, 13.7, 10.9	38.5	2.75, 1H, dd, 14.3, 10.8	38.4
9b	2.49, 1H, d, 13.7		2.50, 1H, dd, 14.3, 1.6	
10		214.0		212.7
11		49.3		49.8
12		125.7		126.5
13	6.18, 1H, s	132.1	6.22, 1H, s	132.1
14		153.7		155.8
15		54.0		54.3
16	4.37, 1H, br s	70.4[a]	4.40, 1H, br s	70.6
17	3.33, 1H, m	59.6	3.39, 1H, embedded	59.1
18		65.6		65.9
19	1.69, 1H, m	39.9	1.70, 1H, m	39.8
20	1.68, 2H, m	22.8	1.68, 2H, m	22.6
21	1.51, 2H, m	19.9	1.51, 2H, m	19.9
22	1.11, 1H, t, 9.3	52.1	1.11, 1H, t, 9.6	52.0
23		38.8		38.9
24a	1.65, 1H, m	41.5	1.65, 1H, m	41.5
24b	0.87, 1H, m		0.87, 1H, m	
25	1.59, 1H, m	17.9	1.59, 1H, m	17.9
	1.46, 1H, m		1.46, 1H, m	
26	1.02, 1H, m	55.6	1.00, 1H, m	55.6
27		39.7		39.4
28a	2.28, 1H, br dd, 13.0, 4.3	40.0	2.28, 1H, br dd, 12.9, 4.2	40.0
28b	1.14, 1H, t, 13.0		1.14, 1H, t, 12.9	
29a	3.69, 1H, d, 10.6	65.1	3.69, 1H, d, 10.9	65.2
29b	3.46, 1H, d, 10.6		3.46, 1H, d, 10.9	
30	0.99, 3H, s	27.0	1.00, 3H, s	26.9
31	0.96, 3H, s	14.4	0.96, 3H, s	14.3
32	0.87, 3H, s	18.3	0.86, 3H, s	18.4
33	1.59, 3H, s	21.9	1.61, 3H, s	22.0
34	1.43, 3H, s	26.5	1.48, 3H, s	26.7
35	0.90, 3H, s	16.4	0.90, 3H, s	16.4
40				
41				
1'	4.64, 1H, dd, 9.8, 2.0	100.3	4.65, 1H, dd, 9.8, 1.9	100.3
2'a	2.35, 1H, ddd, 12.3, 4.3, 2.0	35.2	2.33, 1H, ddd, 12.4, 4.3, 1.9	35.1
2'b	1.49, 1H, m		1.49, 1H, m	

3'	3.17, 1H, m	80.6	3.18, 1H, m	80.6
4'	3.16, 1H, m	75.2	3.15, 1H, m	75.3
5'	3.34, 1H, m	71.8	3.35, 1H, dq, 8.8, 6.2	71.8
6'	1.34, 3H, d, 6.1	18.0	1.34, 3H, d, 6.2	17.9
7'	3.40, 3H, s	56.4	3.40, 3H, s	56.4
1''	3.63, 1H, m	50.1	3.88, 1H, br s	45.1
2''	1.21, 3H, d, 6.5	20.2	1.29, 3H, d, 6.5	22.8
3''a	1.57, 1H, m	29.8	1.27, 3H, d, 6.5	22.5
3''b	1.26, 1H, m			
4''	0.92, 3H, t, 7.3	10.3		

[a] estimated from HSQC/HMBC data collected at 700 MHz

Table S6. ^{1}H (700 MHz) and ^{13}C (175 MHz) NMR data (DMSO-*d*$_6$) of persicamidine A aglycon formed via Payne-type rearrangement.

#	**δ_H**	**δ_C[a]**
1	3.36, 1H, embedded	67.5
2	2.85, 1H, br d, 8.2	84.2
3		43.0
4	0.98, 1H, m	62.2
5a	2.54, 1H, embedded	19.6
5b	1.30, 1H, m	
6a	167, 1H, m	44.6
6b	1.06, 1H, m	
7		35.8
8	1.87, 1H, d, 8.8	42.0
9a	2.49, 1H, embedded	36.7
9b	2.25, 1H, m	
10		210.7
11		49.9
12		124.4
13	6.73, 1H, s	134.5
14		151.0
15		51.9
16	4.00, 1H, d, 5.6	85.4
17	4.21, 1H, t, 5.6	74.3
18		94.7
19	2.25, 1H, m	42.7
20	1.58, 1H, m	17.8
	1.45, 1H	
21	1.45, 1H, m	19.3
	1.36, 1H, m	
22	1.31, 1H, m	50.4
23		38.2
24a	1.56, 1H, m	41.4
24b	0.87, 1H, m	
25a	1.47, 1H, m	17.6
25b	1.27, 1H, m	
26	0.92, 1H, m	55.2

27		39.0
28a	1.93, 1H, br dd, 12.9, 4.3	41.6
28b	0.83, 1H, m	
29a	3.40, 1H, embedded	62.8
29b	3.16, 1H, d, 10.4	
30	0.85, 3H, s	27.2
31	0.80, 3H, s	14.3
32	0.78, 3H, s	18.4
33	1.40, 3H, s	25.1
34	1.14, 3H, s	25.8
35	0.85, 3H, s	16.9
1''	3.33, 1H, embedded	47.8
2''	1.00, 3H, d, 6.5	19.4
3''a	1.45, 1H, m	28.3
3''b	1.28, 1H, m	
4''	0.79, 3H, m	10.3
2-OH	4.03, br s	
17-OH	4.95, d, 5.6	
29-OH	4.27, br s	
40-NH	5.78, br d, 8.1	

[a] estimated from HSQC/HMBC data collected at 700 MHz

Table S7. Mass spectrometry data deposited in MassIVE repository

Dataset	Comments
C4NS15-E_BD3_01_10178_analysis.baf	MS data for fraction containing persicamidines A-E
4NS15-Fr3-1-813_BD5_01_11312_analysis.baf	MS data for persicamidine A
LK-4NS15-813-aglycone_GB2_1_8329_analysis.baf	MS data for persicamidine A rearranged aglycon
LK-4NS15-799-Fr2_BB5_1_3359_analysis.baf	MS data for persicamidine B
4NS15-Fr4-827_BD7_01_11314_analysis.baf	MS data for persicamidine C
4NS15-Fr5-841_BD8_01_11315_analysis.baf	MS data for persicamidine D
4NS15-Fr1-785_BD3_01_11310_analysis.baf	MS data for persicamidine E

Mass spectrometry data files used during structure elucidation have been submitted to the MassIVE repository with public accession number MSV000090724 (download link: ftp://massive.ucsd.edu/MSV000090724/)

NMR figures

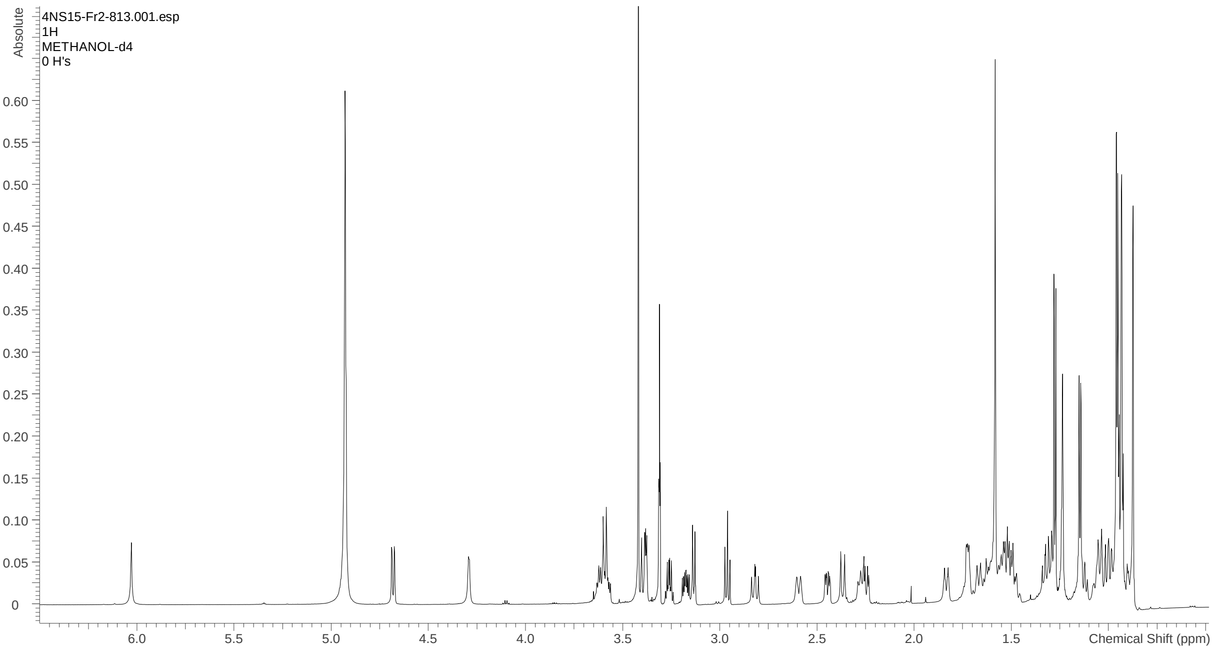

Figure S6. ^{1}H NMR spectrum of persicamidine A (**1**) in CD_3OD (700 MHz)

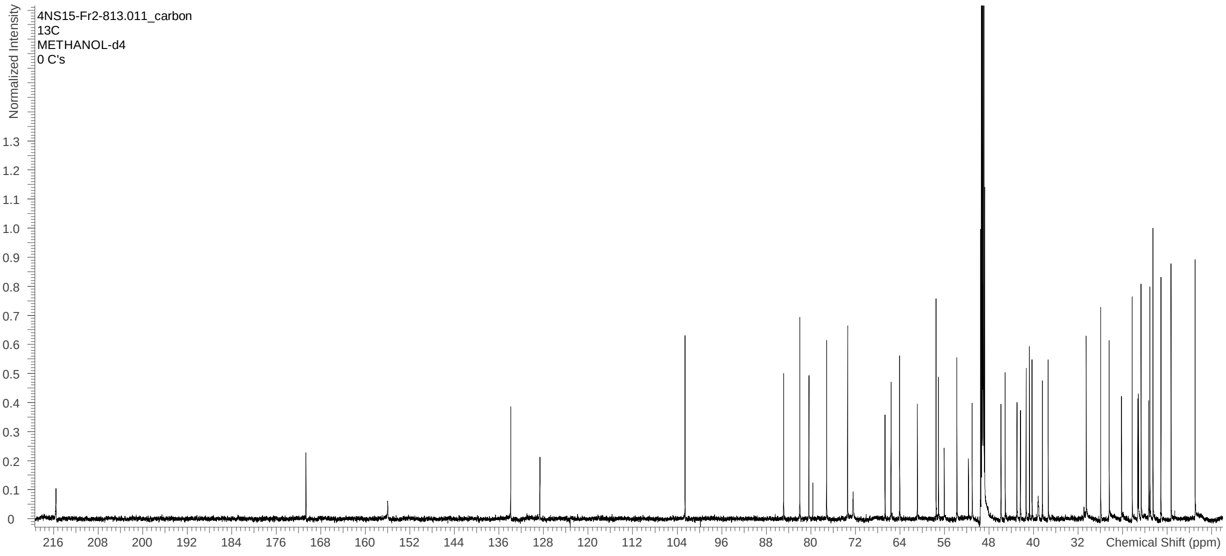

Figure S7. ^{13}C NMR spectrum of persicamidine A (**1**) in CD_3OD (175 MHz)

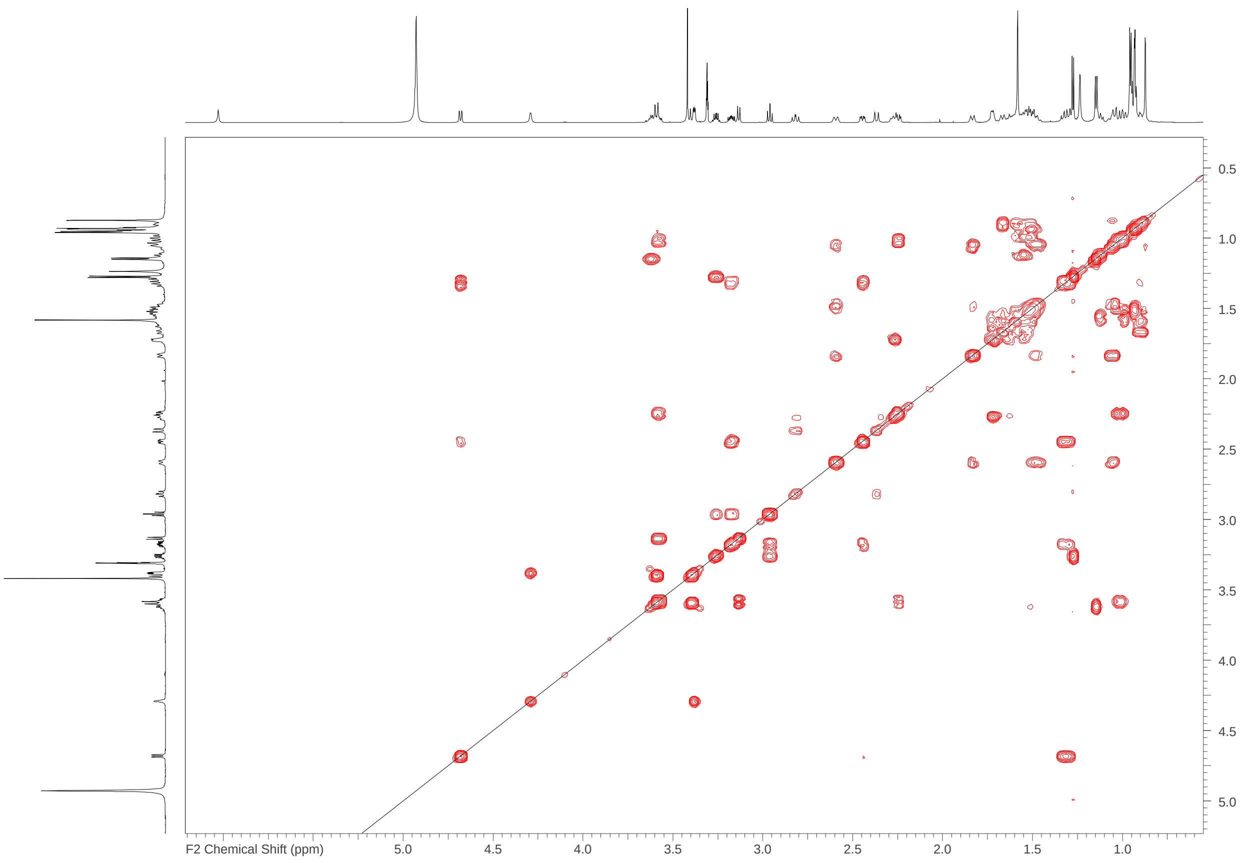

Figure S8. COSY NMR spectrum of persicamidine A (**1**) in CD_3OD (700 MHz)

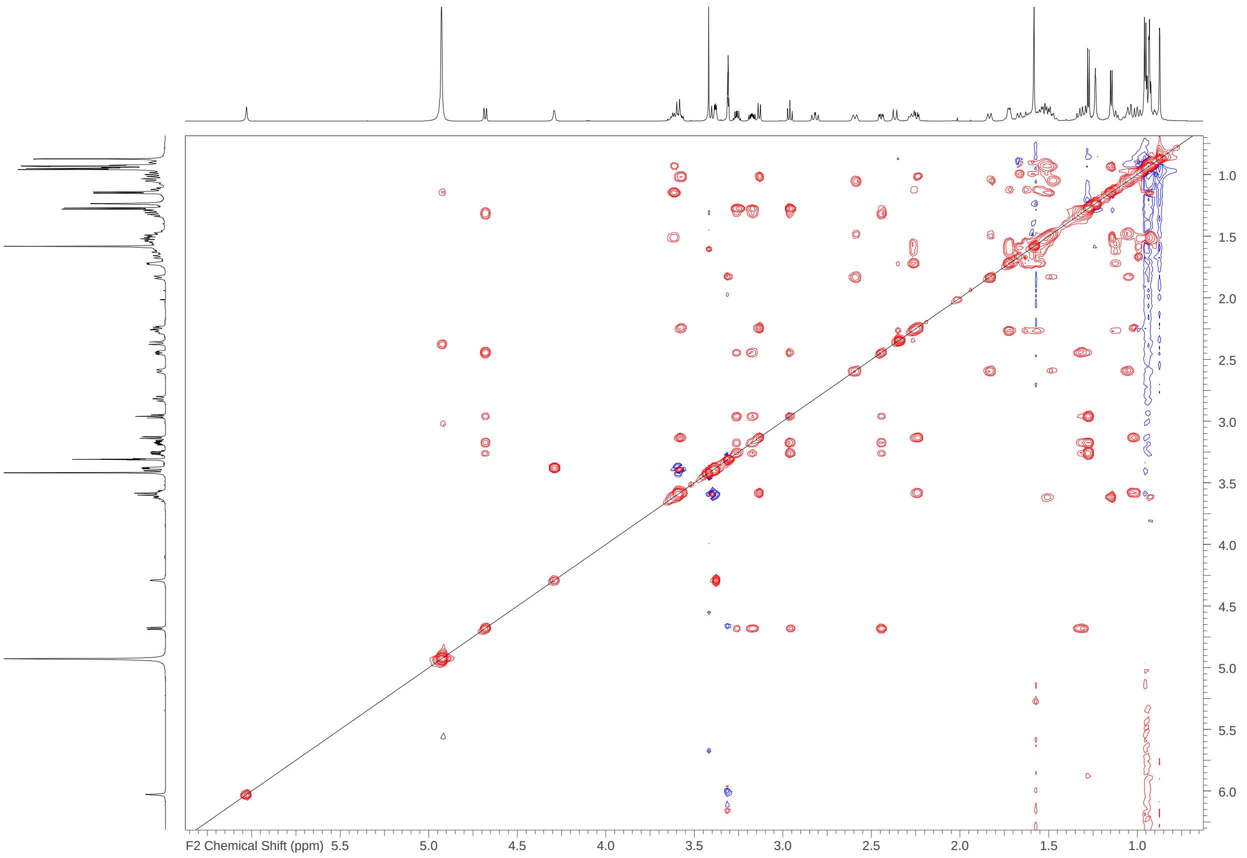

Figure S9. TOCSY NMR spectrum of persicamidine A (**1**) in CD_3OD (700 MHz)

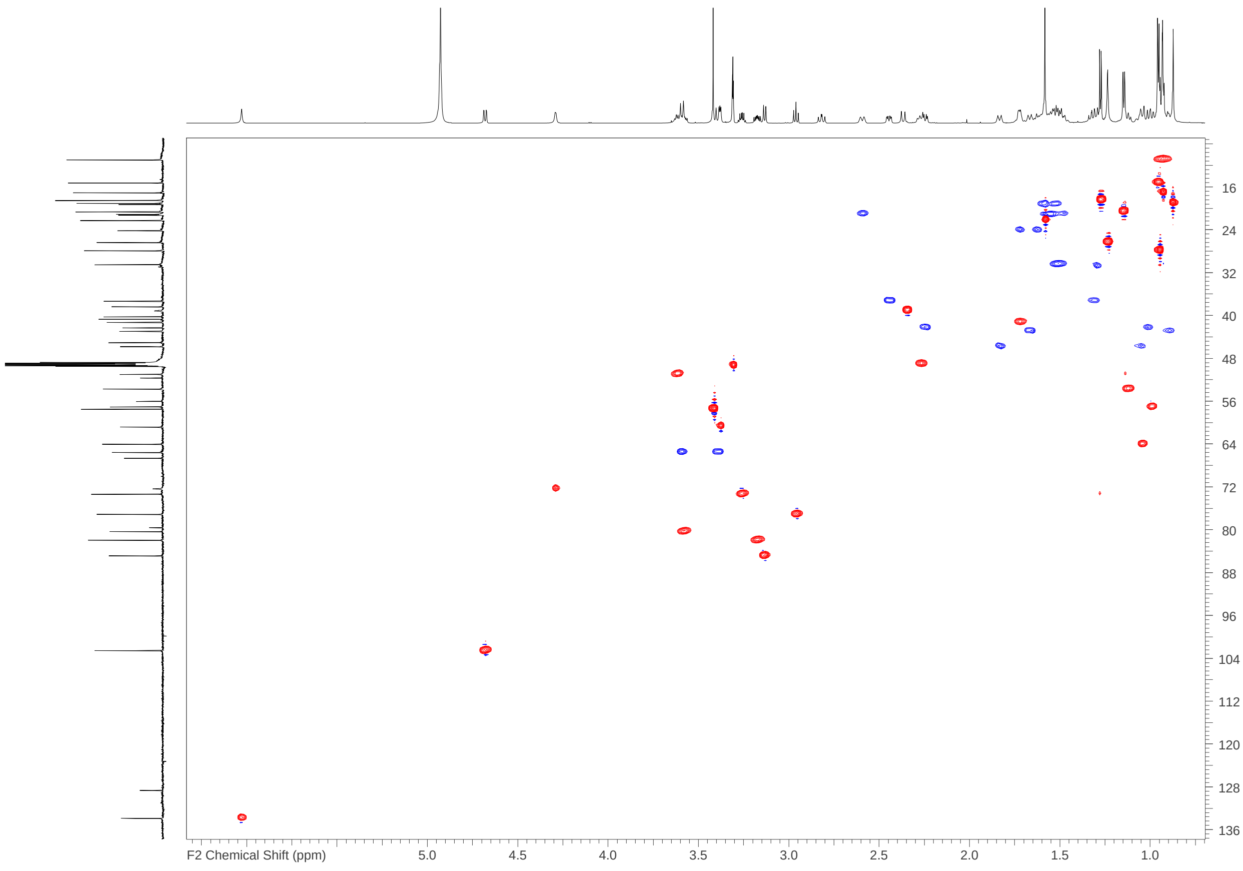

Figure S10. HSQC NMR spectrum of persicamidine A (**1**) in CD_3OD (700 MHz)

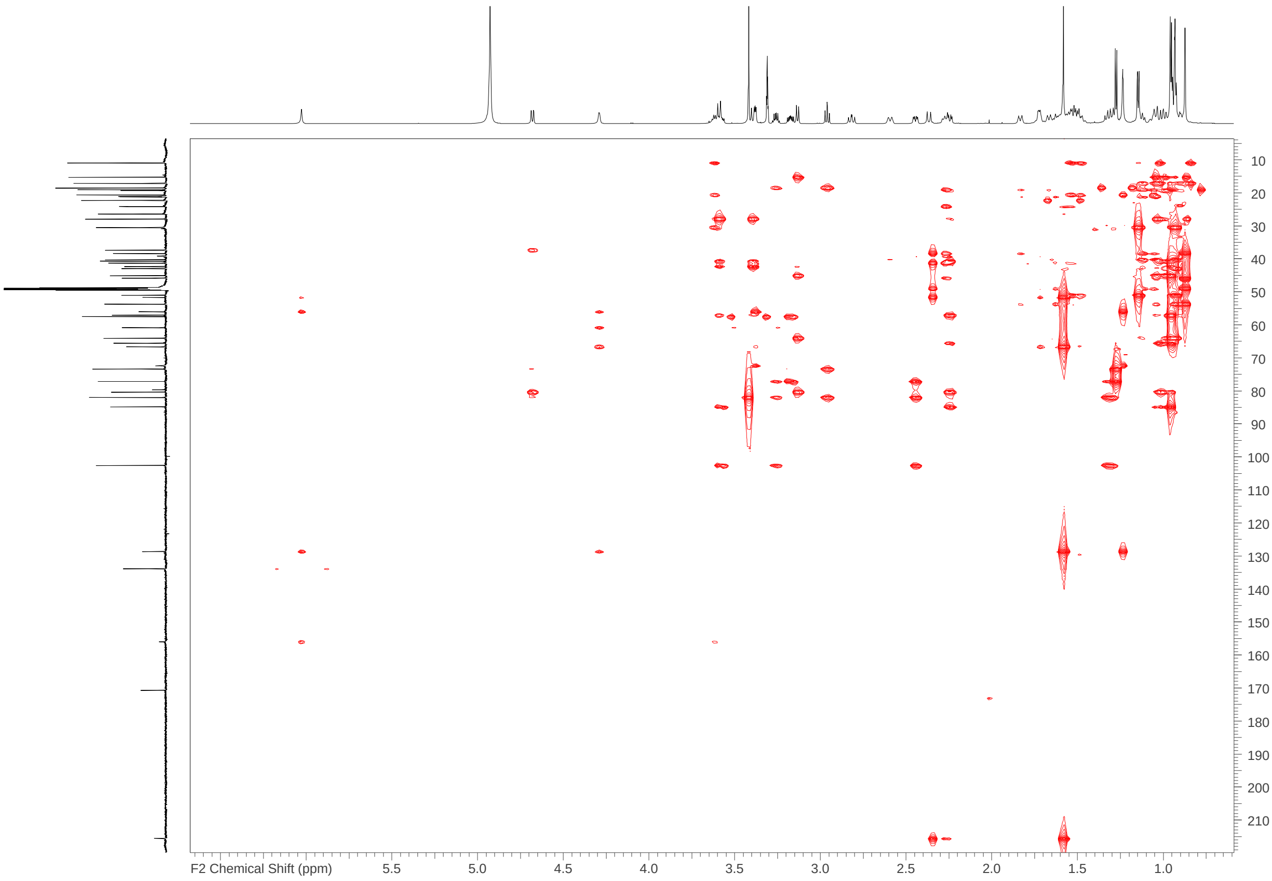

Figure S11. HMBC NMR spectrum of persicamidine A (**1**) in CD_3OD (700 MHz)

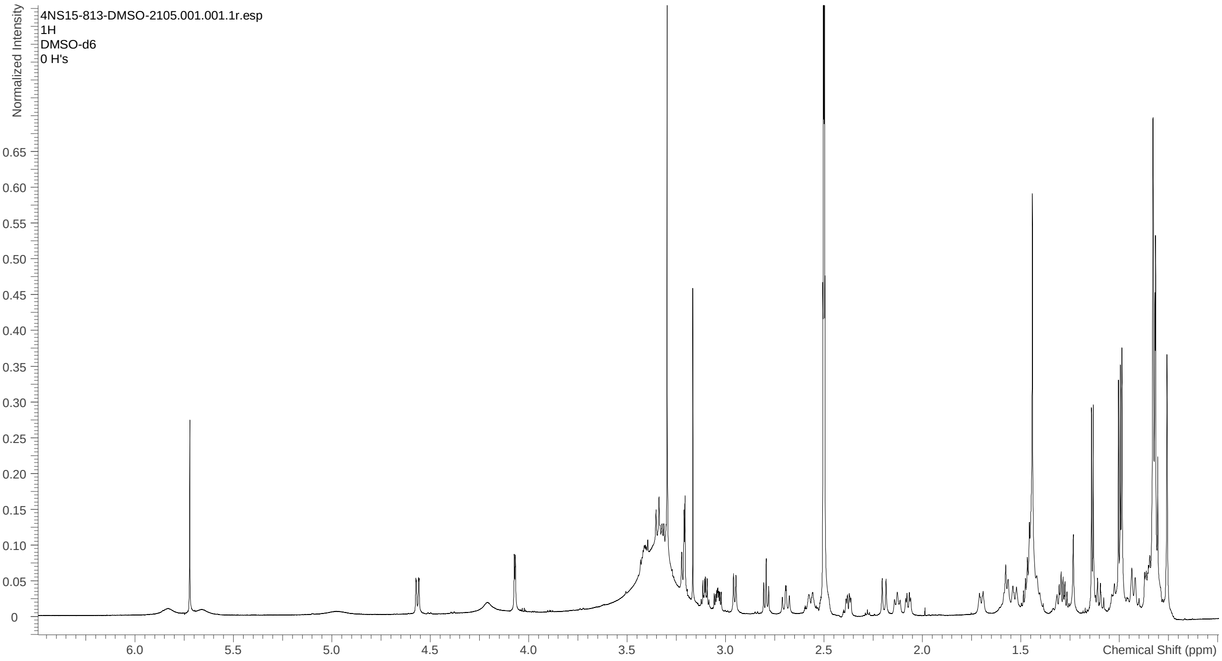

Figure S12. ^{1}H NMR spectrum of persicamidine A (**1**) in DMSO-d_6 (700 MHz)

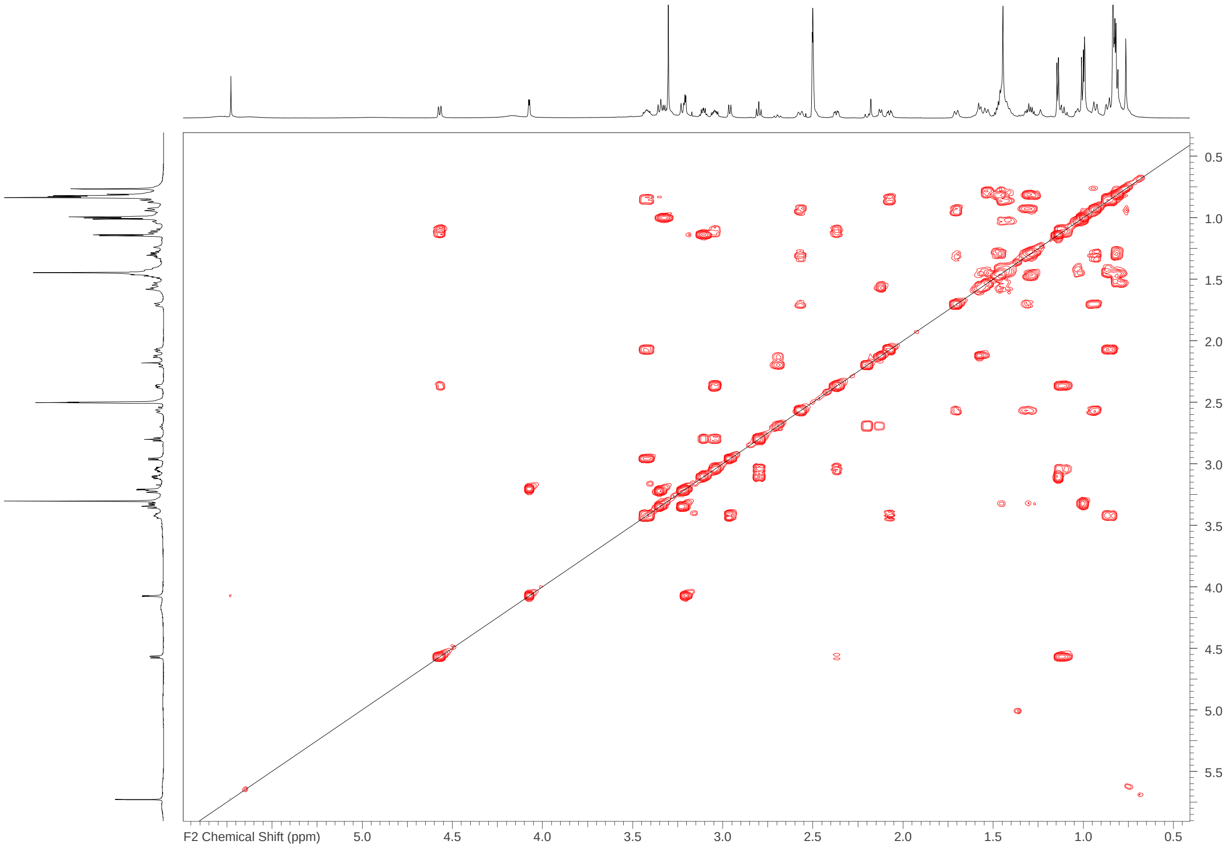

Figure S13. COSY NMR spectrum of persicamidine A (**1**) in DMSO-d_6 (700 MHz)

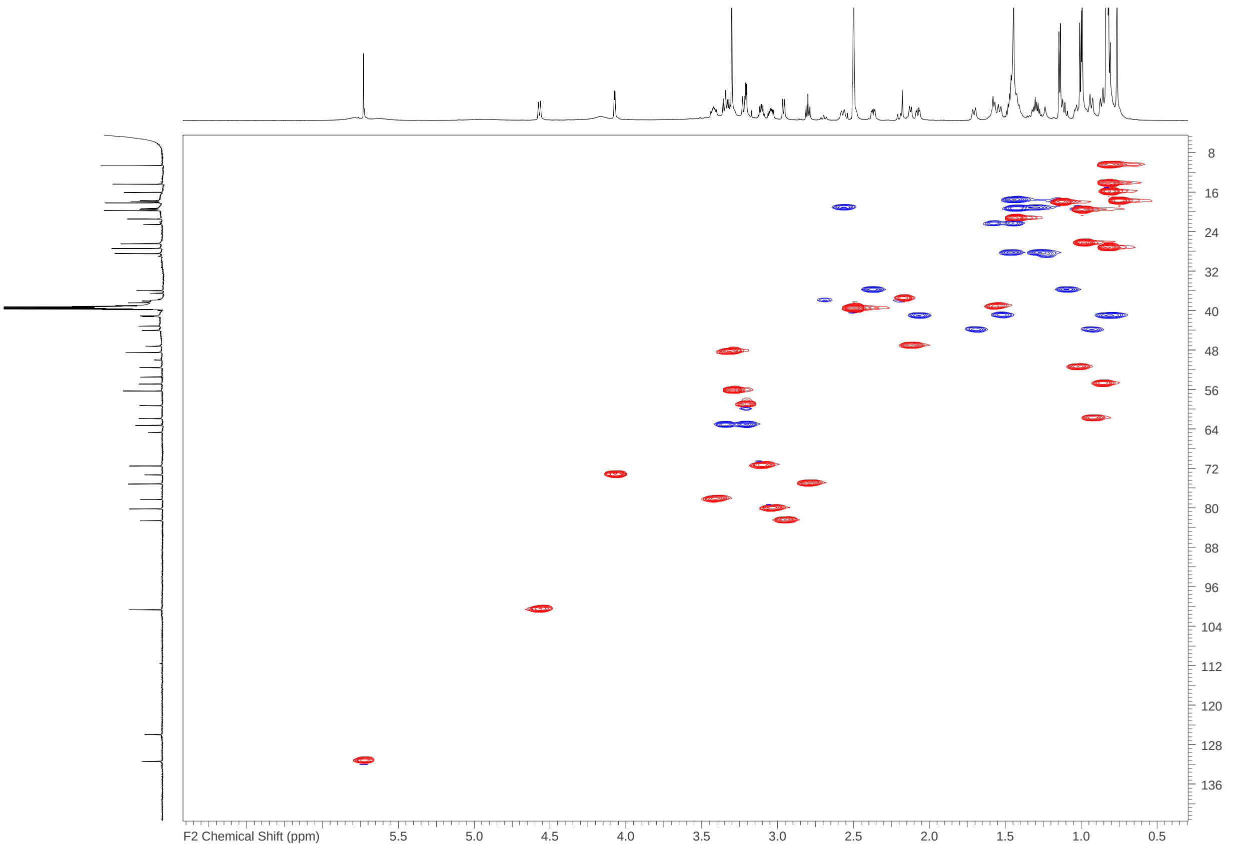

Figure S14. HSQC NMR spectrum of persicamidine A (**1**) in DMSO-d_6 (700 MHz)

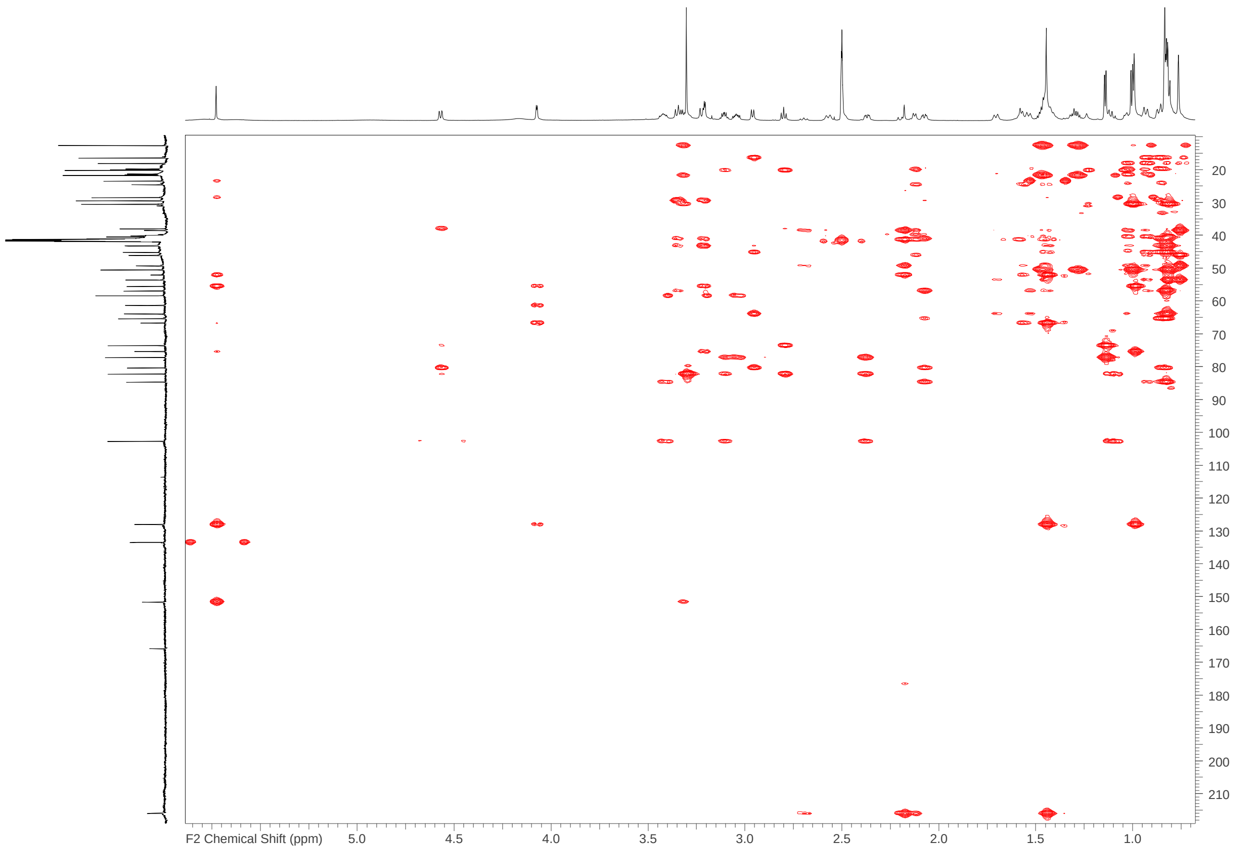

Figure S15. HMBC NMR spectrum of persicamidine A (**1**) in DMSO-d_6 (700 MHz)

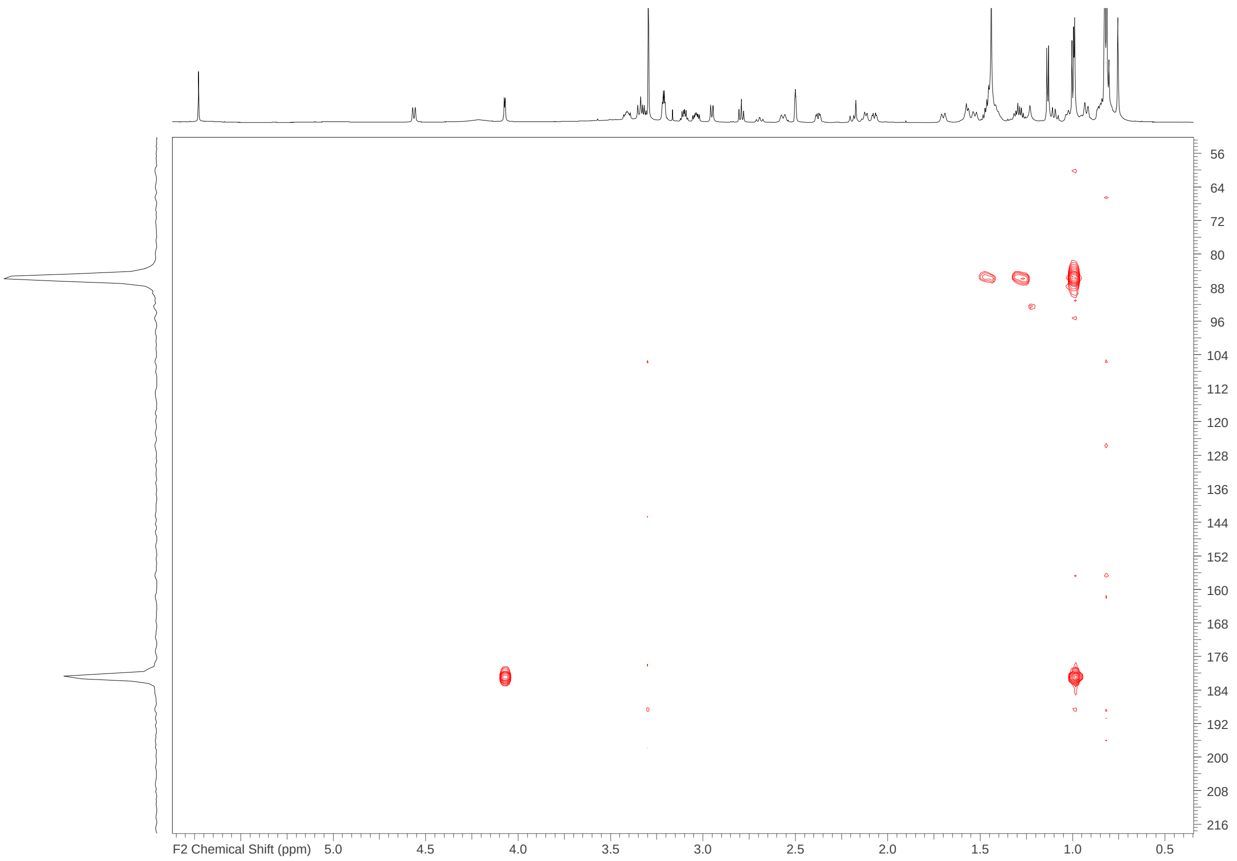

Figure S16. ^{1}H-^{15}N HMBC spectrum of persicamidine A (**1**) in DMSO-d_6 (700 MHz)

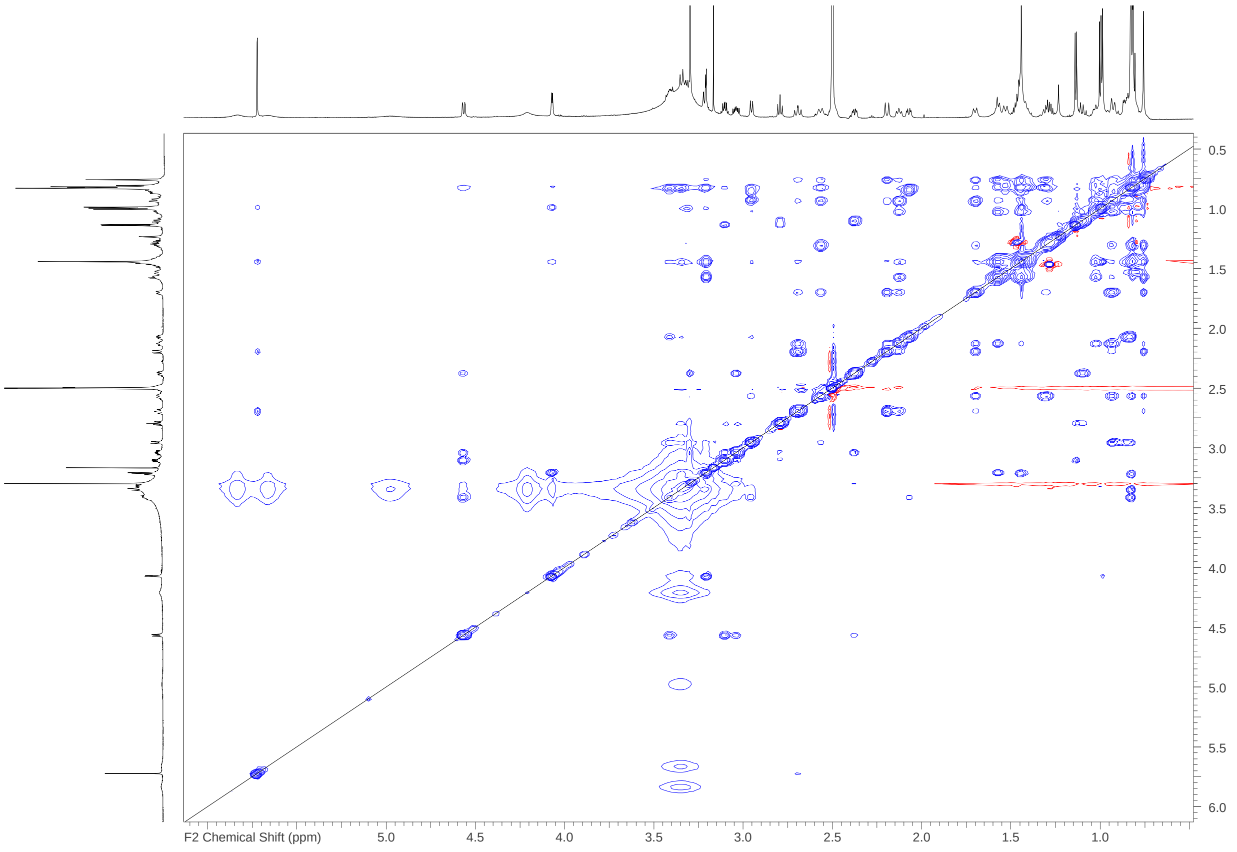

Figure S17. NOESY NMR spectrum of persicamidine A (**1**) in DMSO-d_6 (700 MHz)

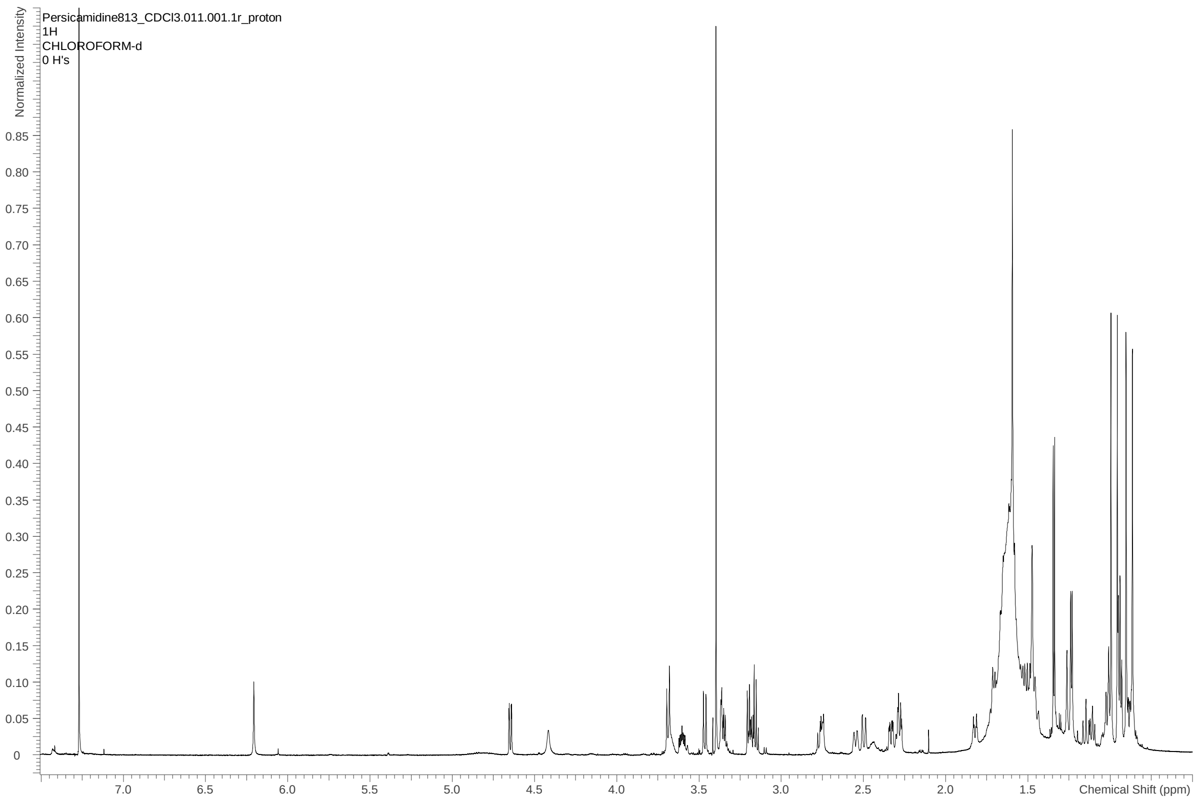

Figure S18. ^{1}H NMR spectrum of persicamidine A (**1**) in $CDCl_3$ (700 MHz)

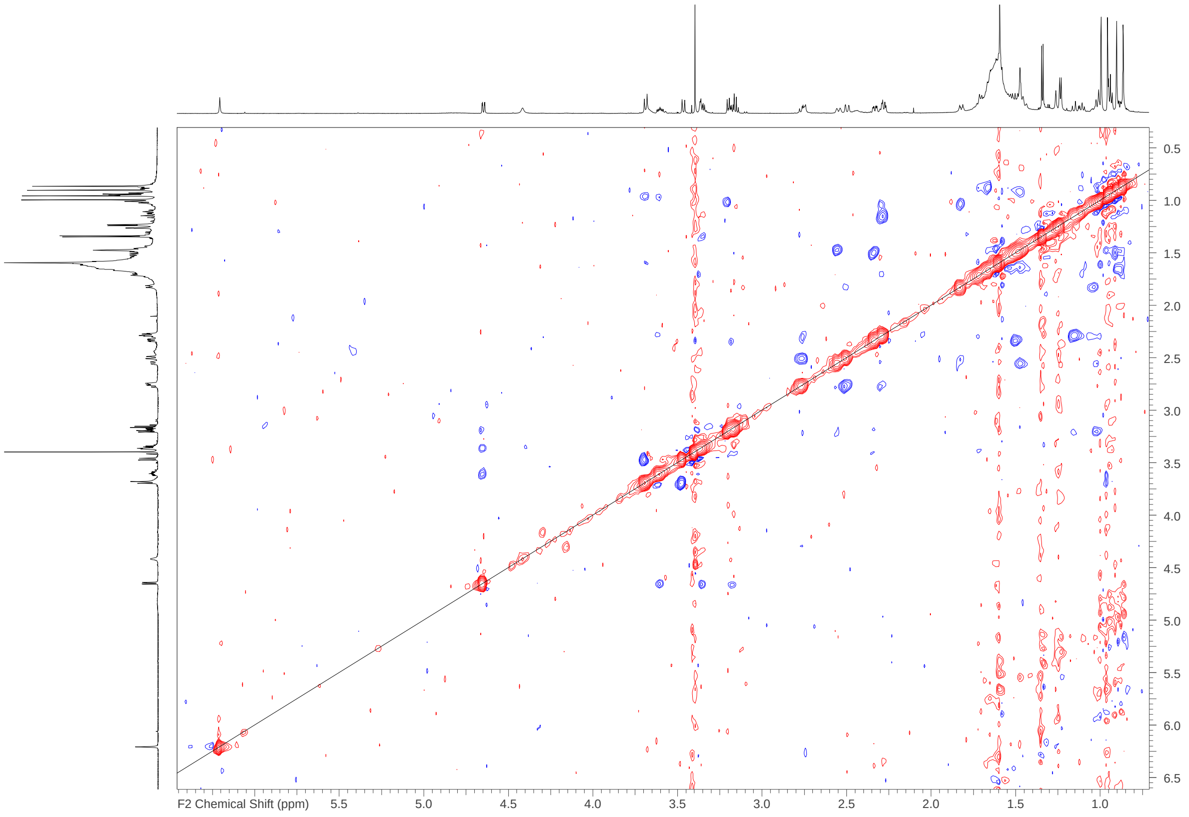

Figure S19. ROESY NMR spectrum of persicamidine A (**1**) in $CDCl_3$ (700 MHz)

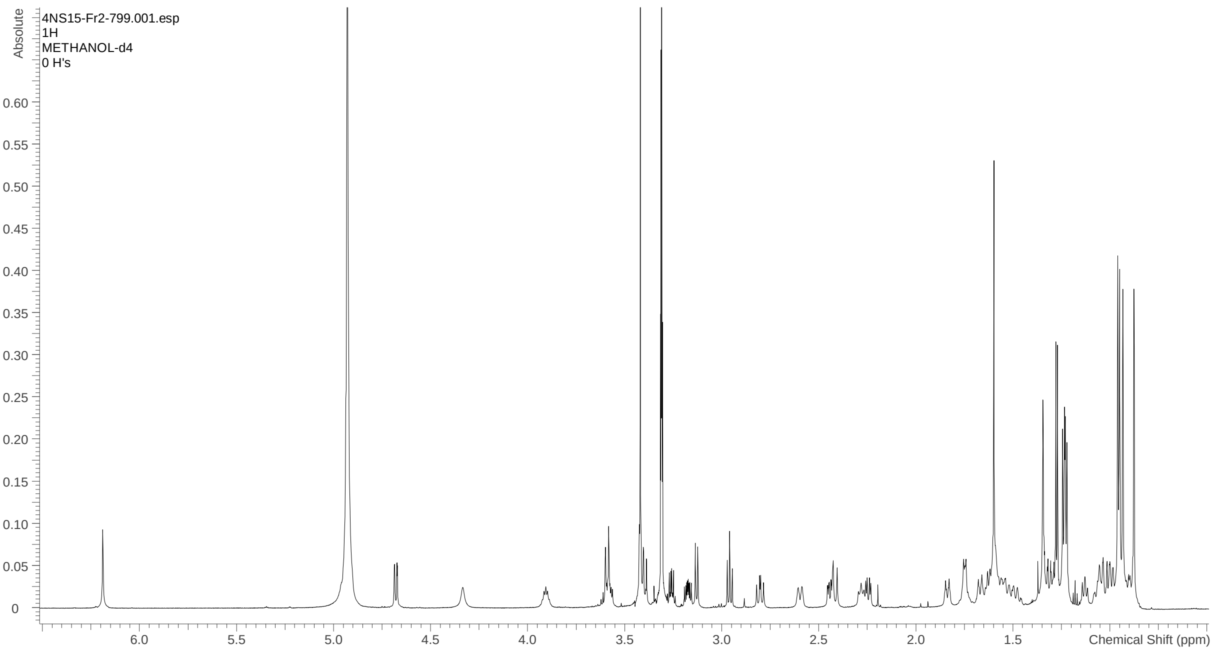

Figure S20. ^{1}H NMR spectrum of persicamidine B (**2**) in CD_3OD (700 MHz)

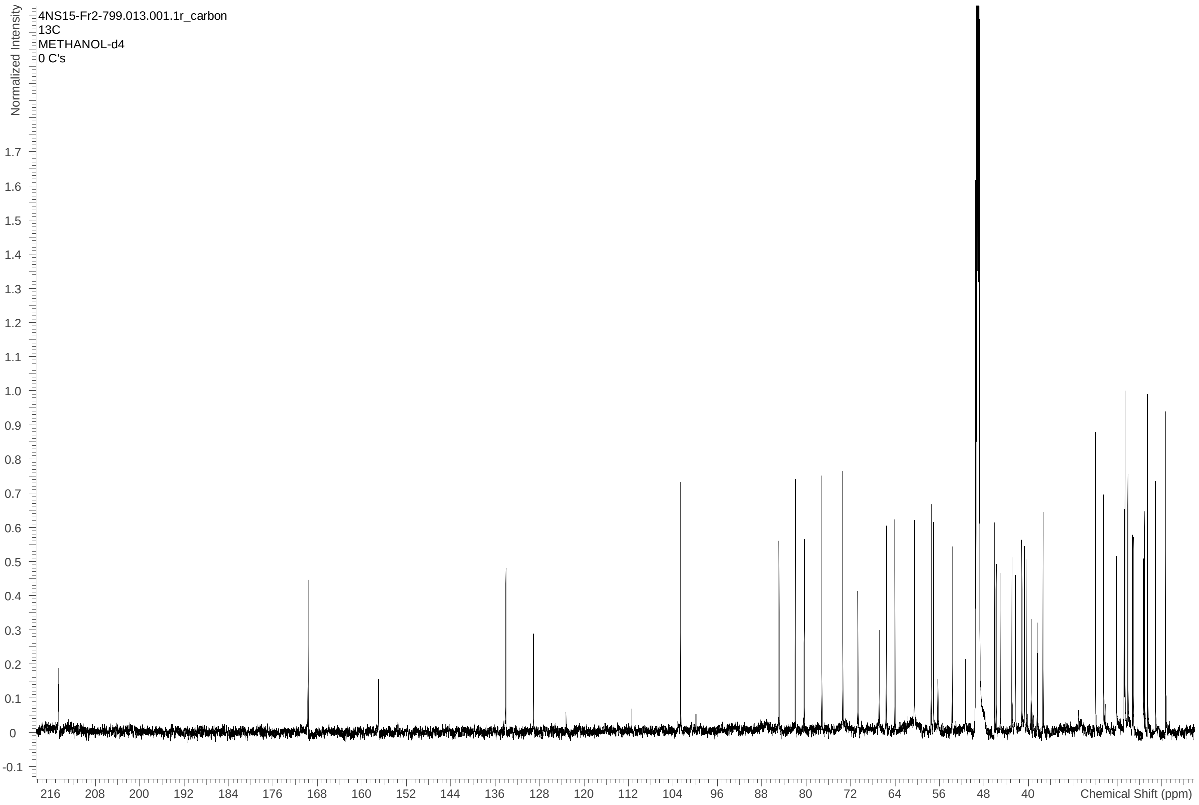

Figure S21. ^{13}C NMR spectrum of persicamidine B (**2**) in CD_3OD (175 MHz)

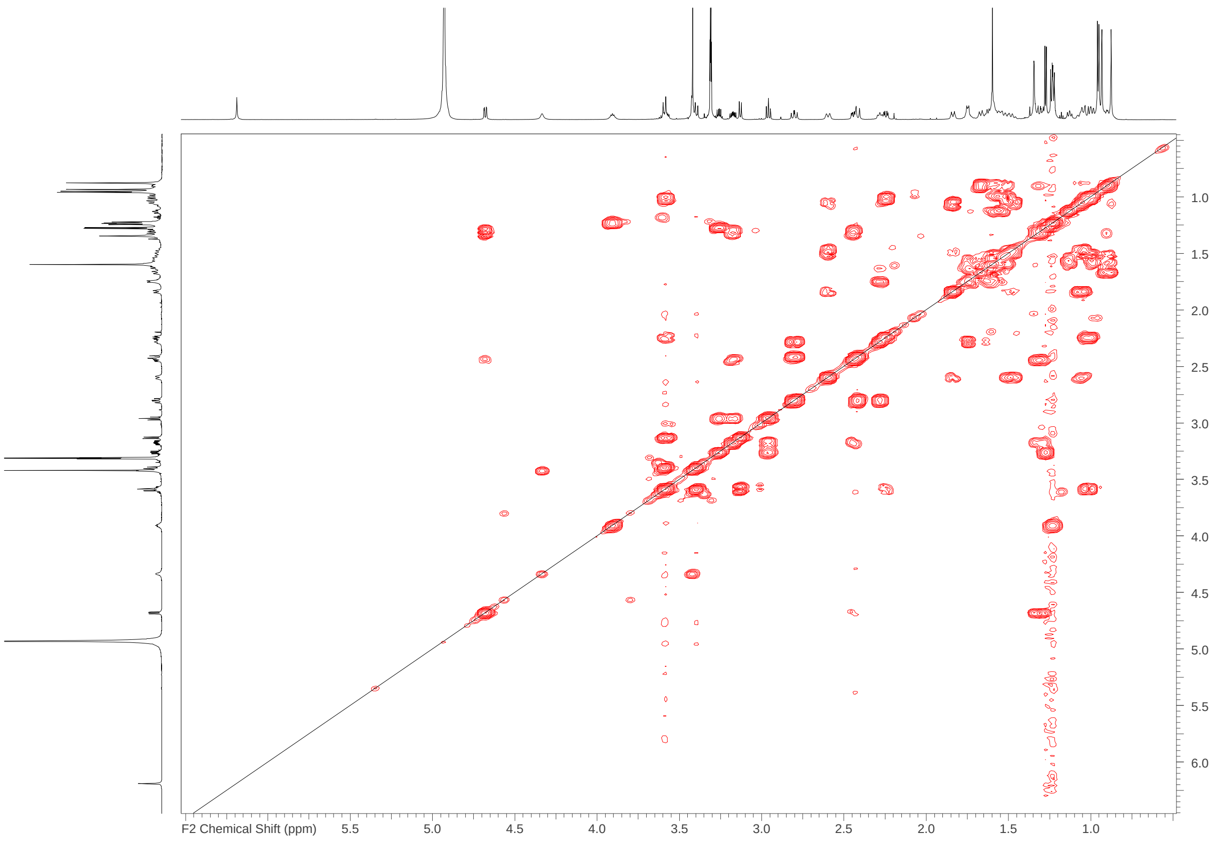

Figure S22. COSY NMR spectrum of persicamidine B (**2**) in CD_3OD (700 MHz)

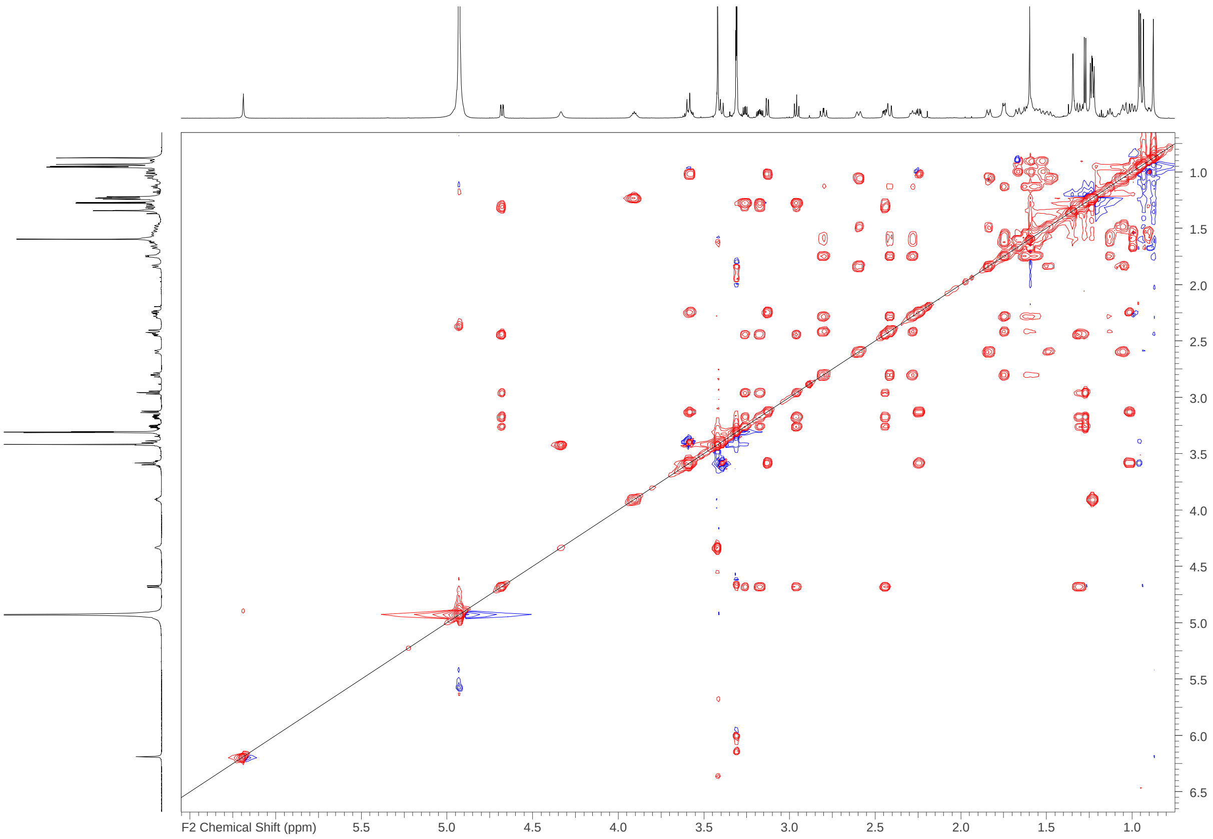

Figure S23. TOCSY NMR spectrum of persicamidine B (**2**) in CD_3OD (700 MHz)

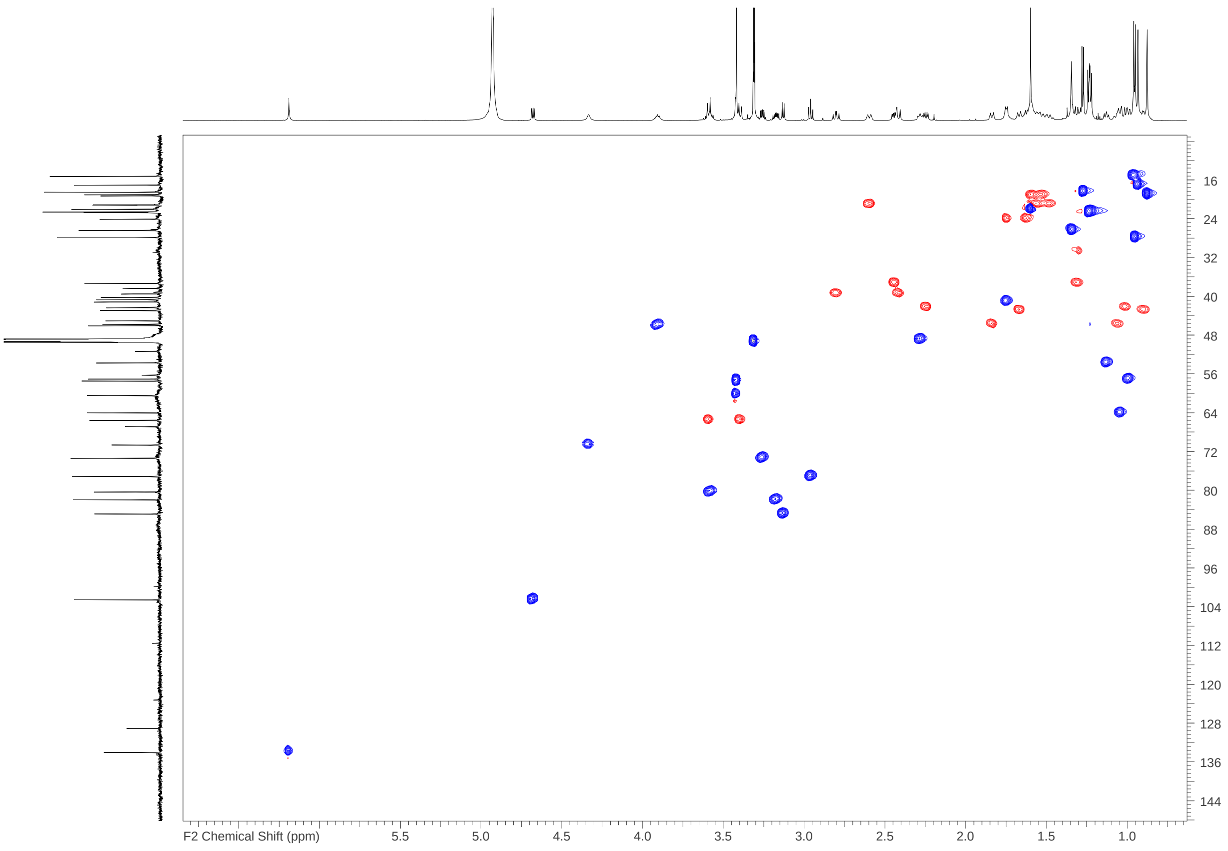

Figure S24. HSQC NMR spectrum of persicamidine B (**2**) in CD_3OD (700 MHz)

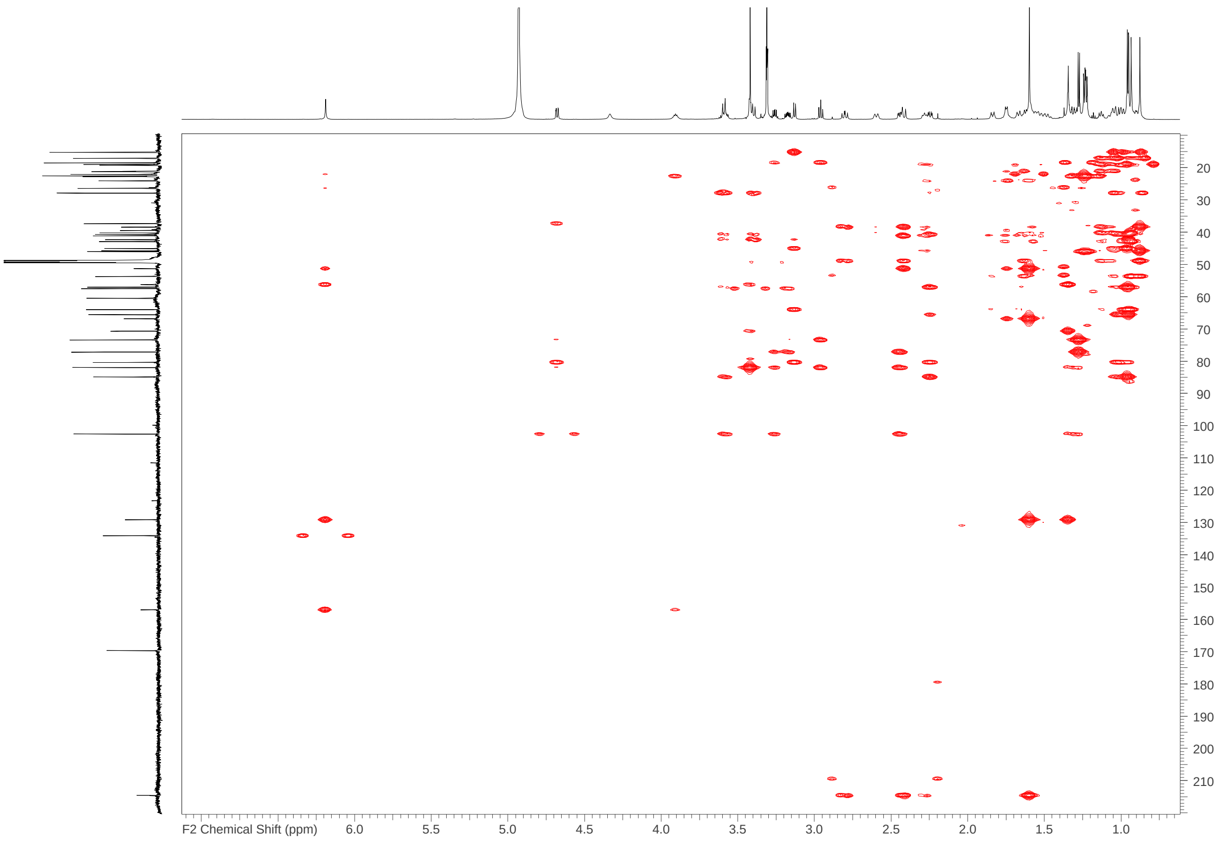

Figure S25. HMBC NMR spectrum of persicamidine B (**2**) in CD_3OD (700 MHz)

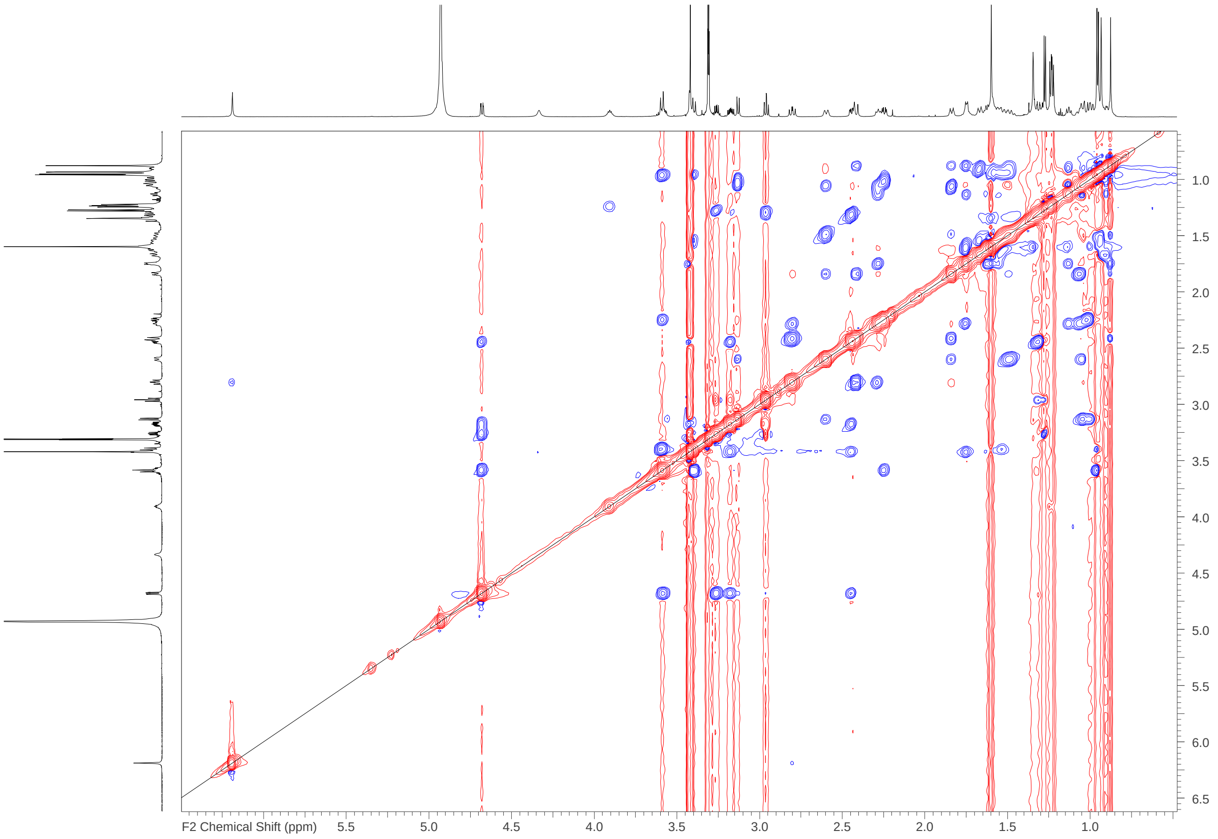

Figure S26. ROESY NMR spectrum of persicamidine B (**2**) in CD_3OD (700 MHz)

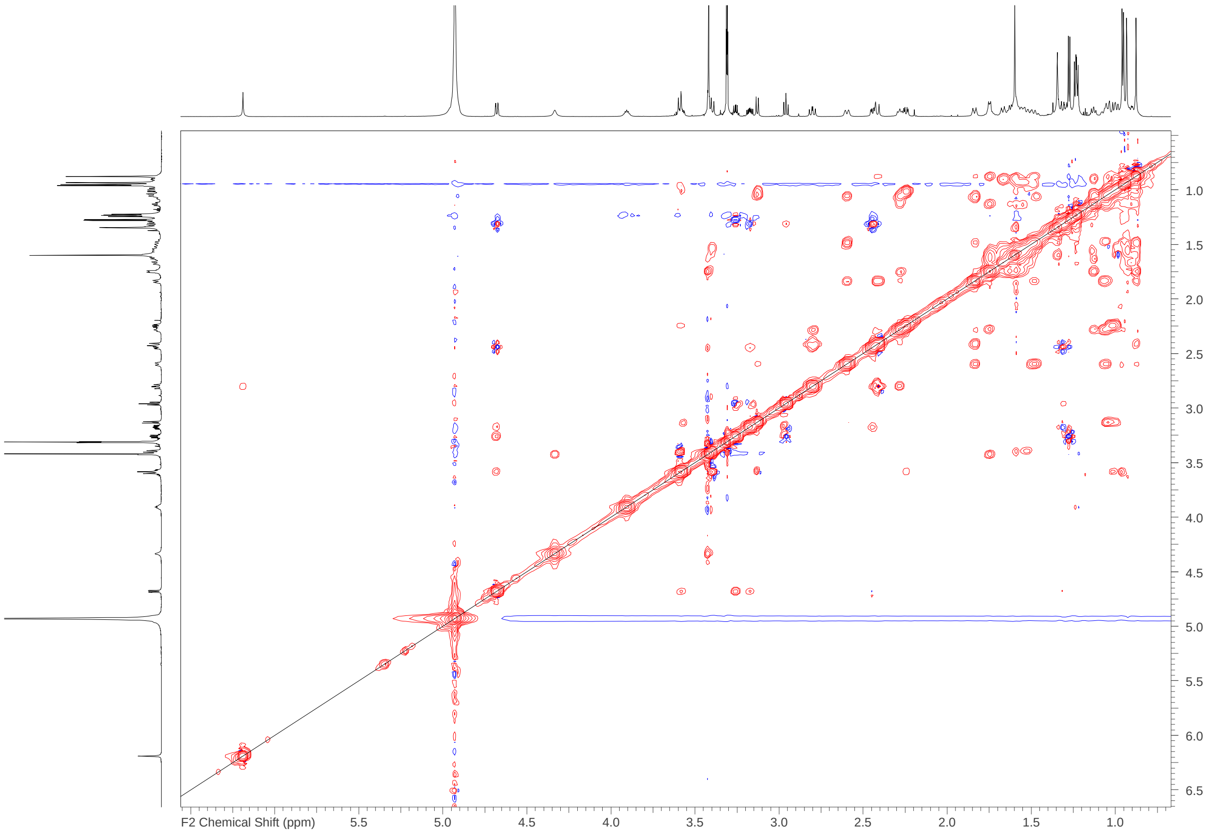

Figure S27. NOESY NMR spectrum of persicamidine B (**2**) in CD_3OD (700 MHz)

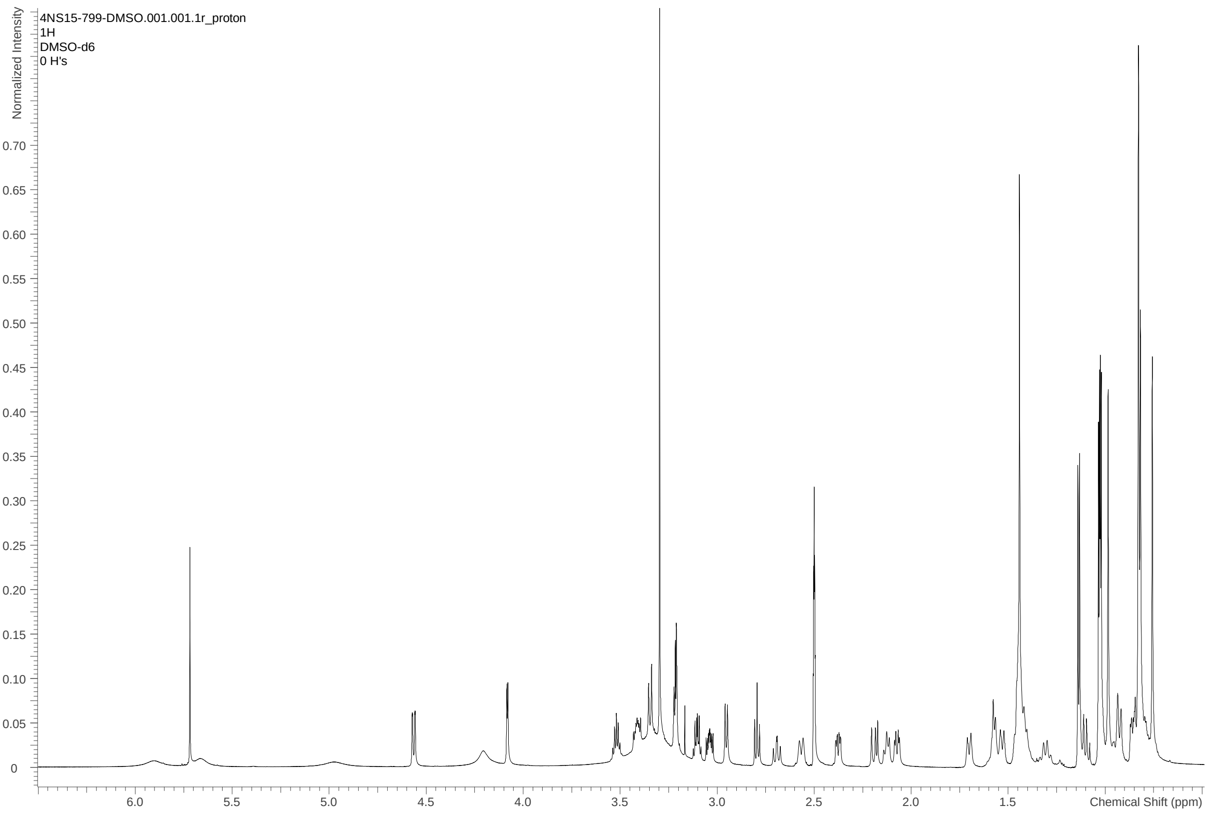

Figure S28. ^{1}H NMR spectrum of persicamidine B (**2**) in DMSO-d_6 (700 MHz)

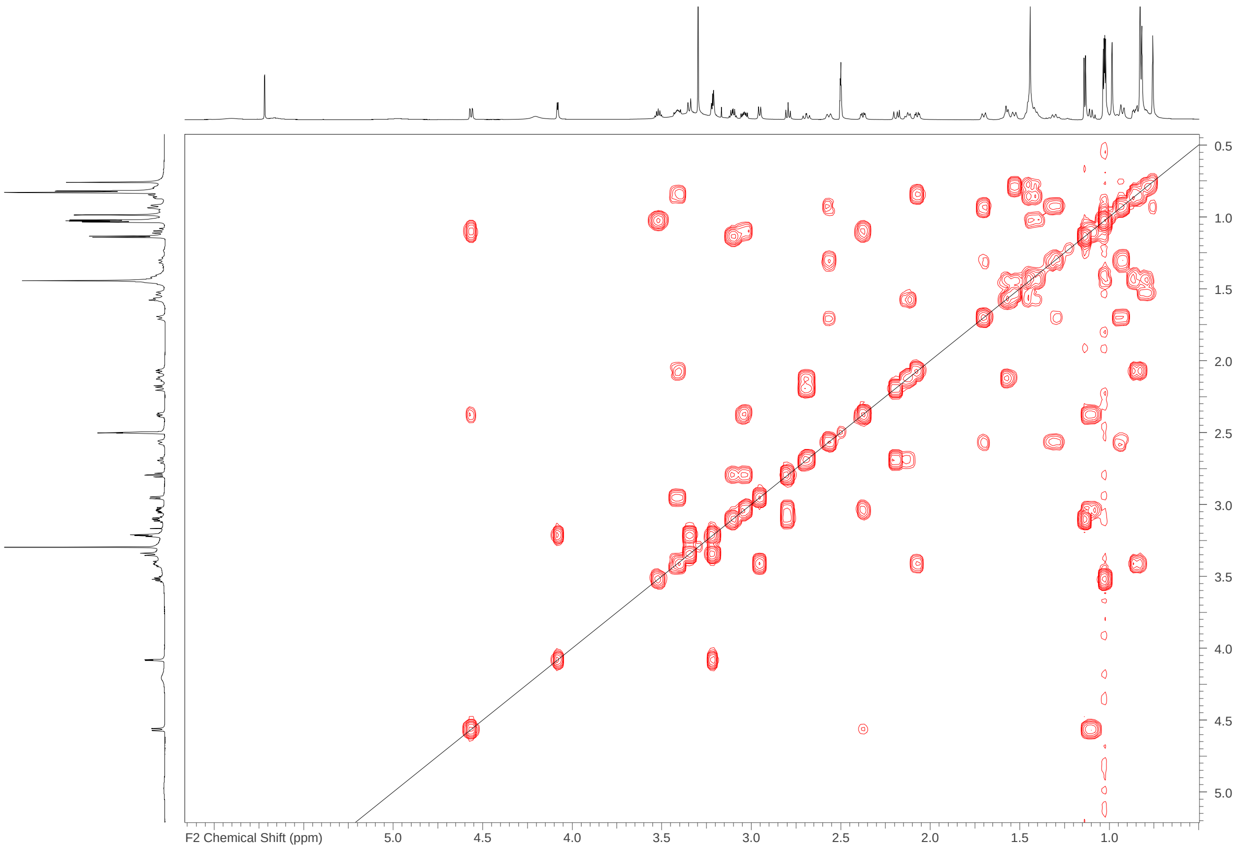

Figure S29. COSY NMR spectrum of persicamidine B (**2**) in DMSO-d_6 (700 MHz)

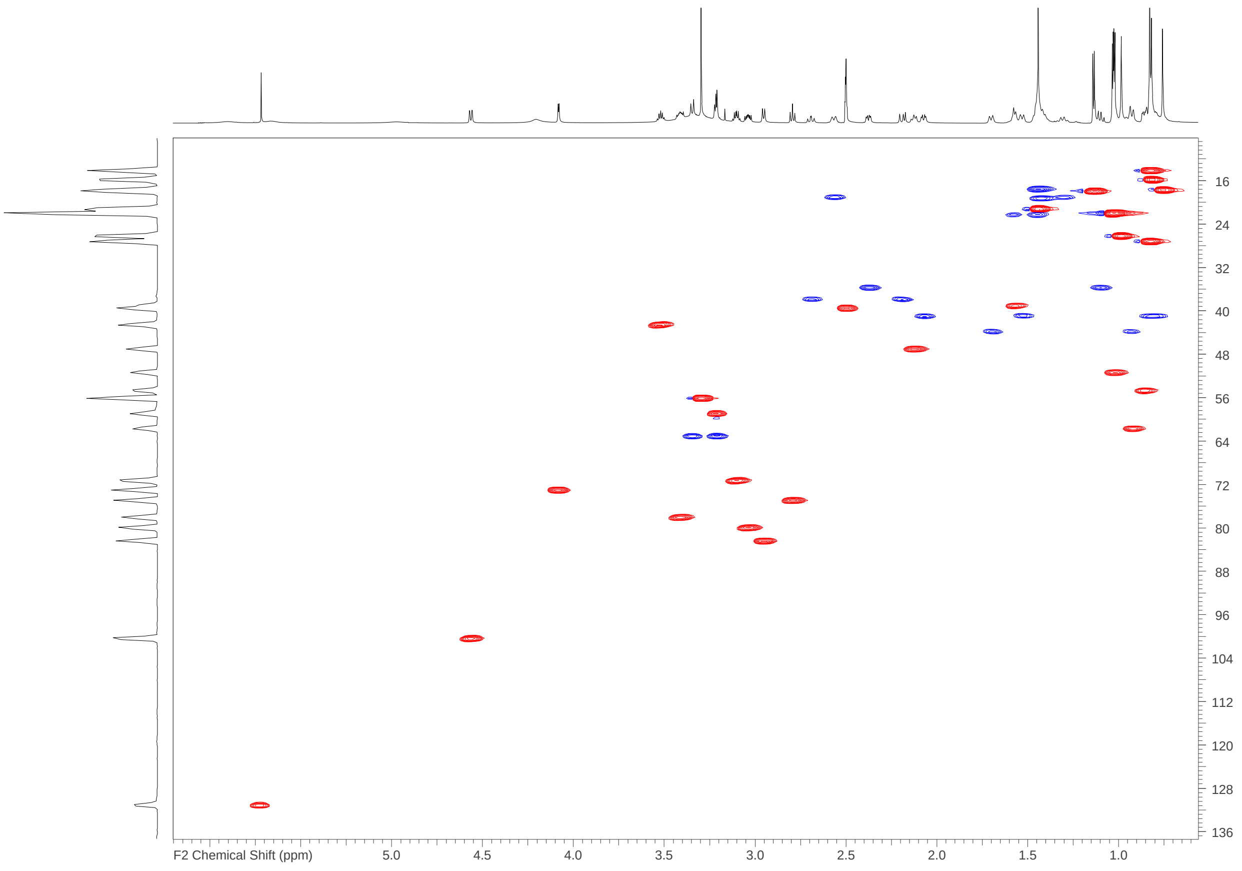

Figure S30. HSQC NMR spectrum of persicamidine B (**2**) in DMSO-d_6 (700 MHz)

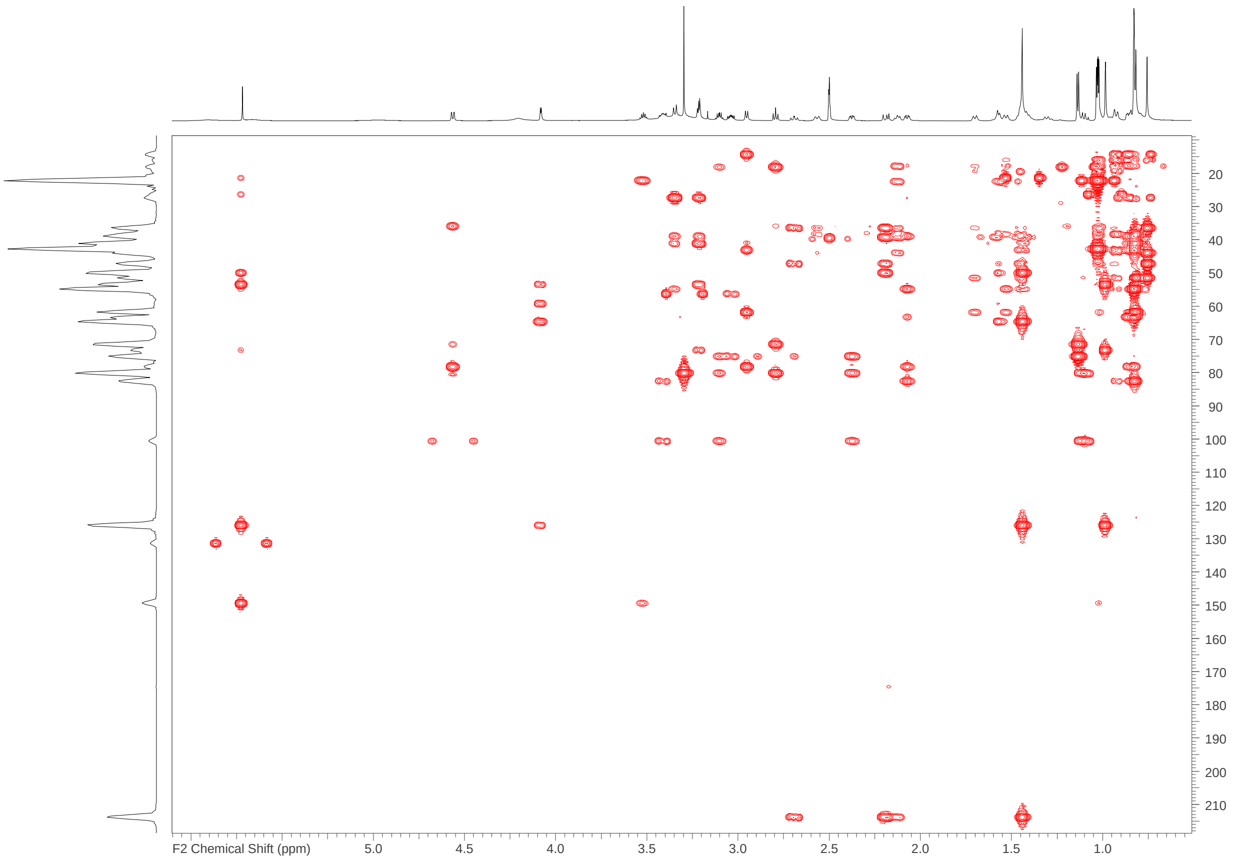

Figure S31. HMBC NMR spectrum of persicamidine B (**2**) in DMSO-d_6 (700 MHz)

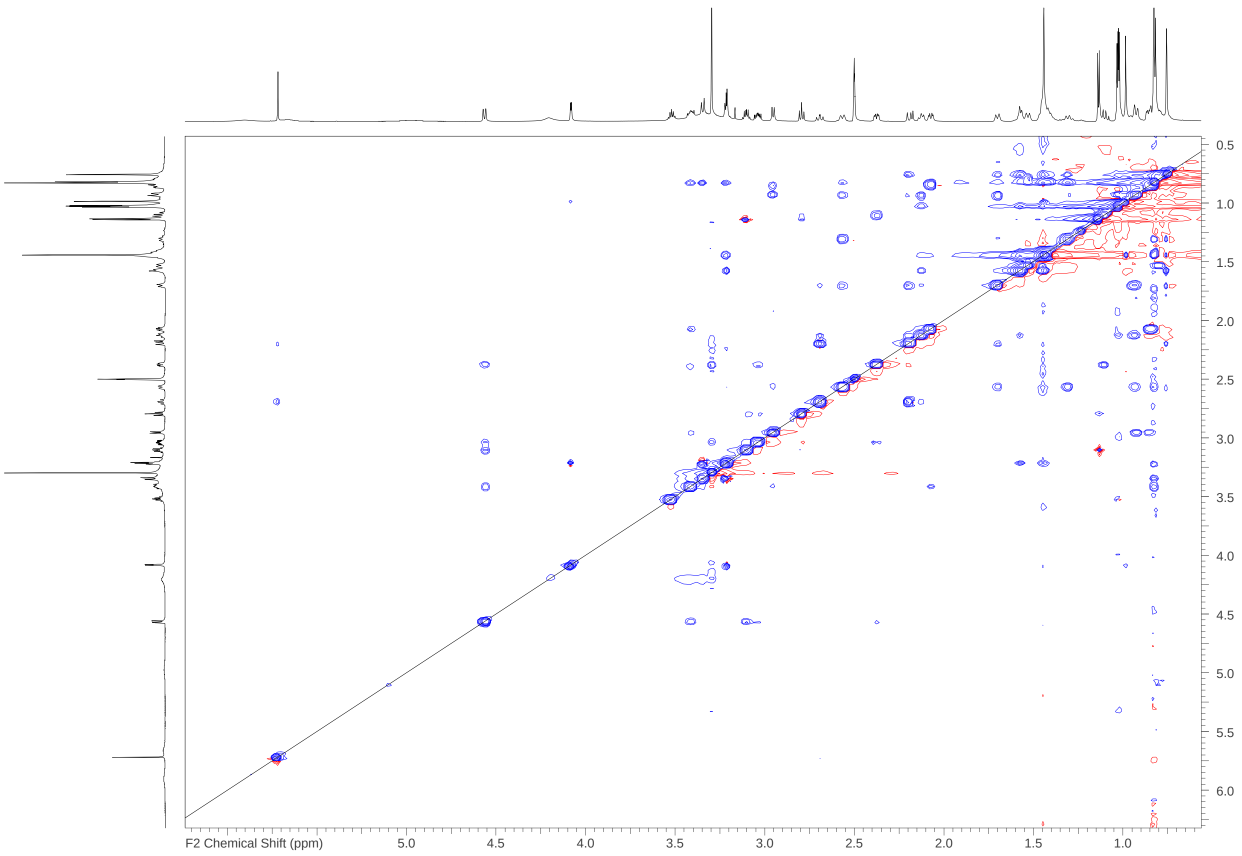

Figure S32. NOESY NMR spectrum of persicamidine B (**2**) in DMSO-d_6 (700 MHz)

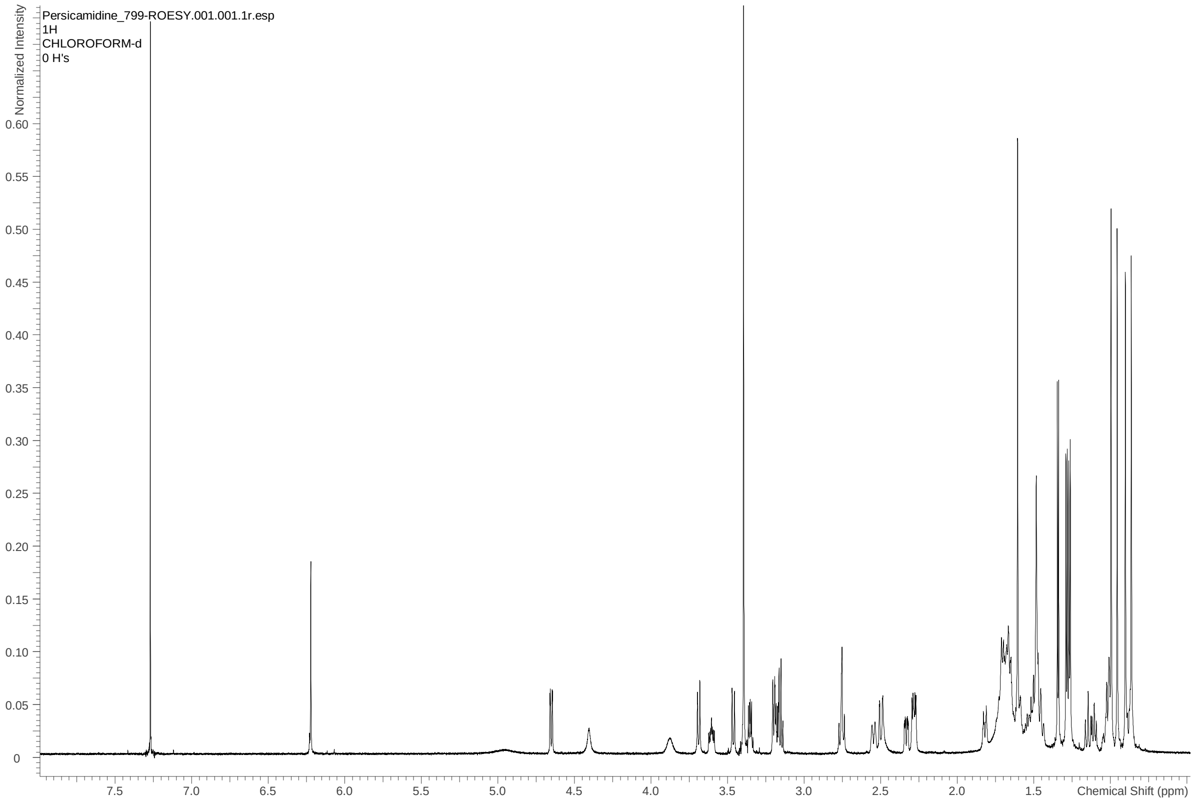

Figure S33. ^{1}H NMR spectrum of persicamidine B (**2**) in $CDCl_3$ (700 MHz)

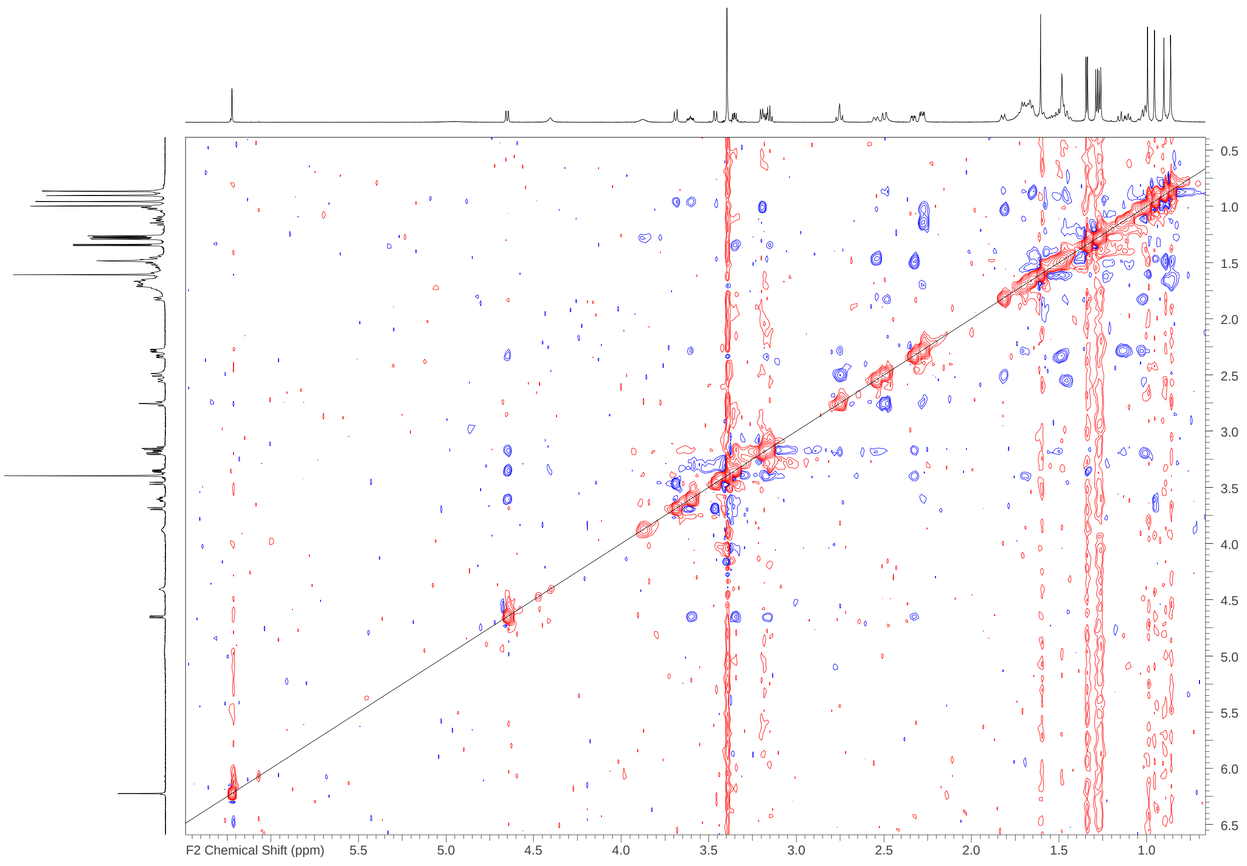

Figure S34. ROESY NMR spectrum of persicamidine B (**2**) in $CDCl_3$ (700 MHz)

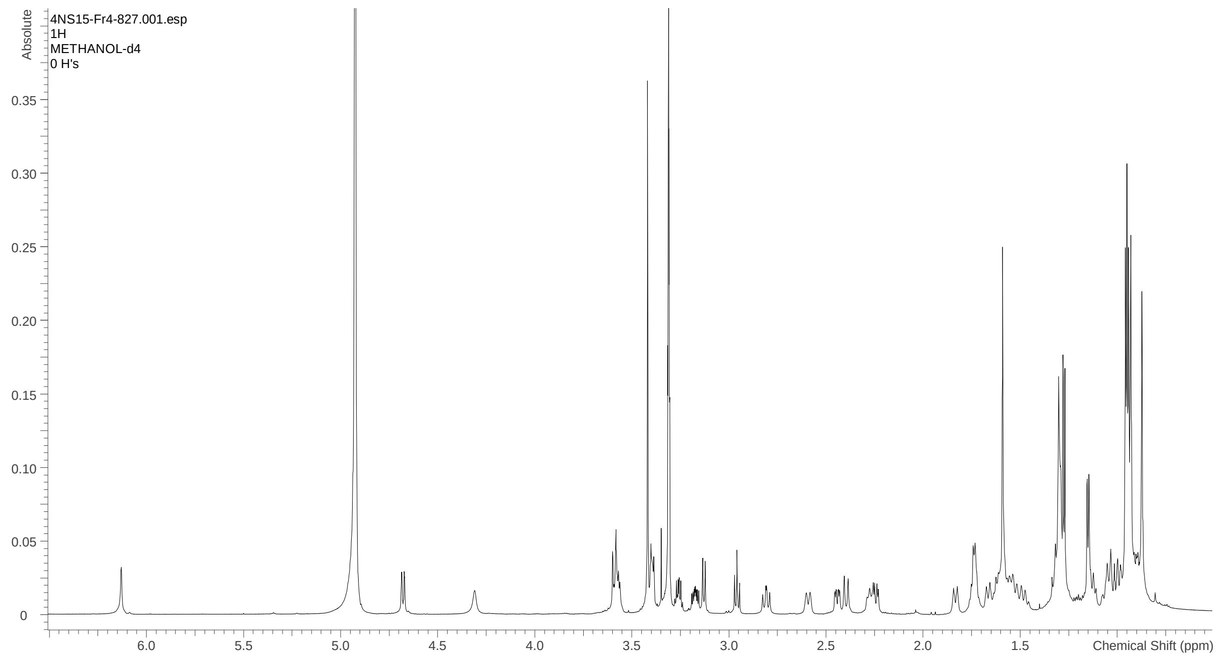

Figure S35. ^{1}H NMR spectrum of persicamidine C (**3**) in CD_3OD (700 MHz)

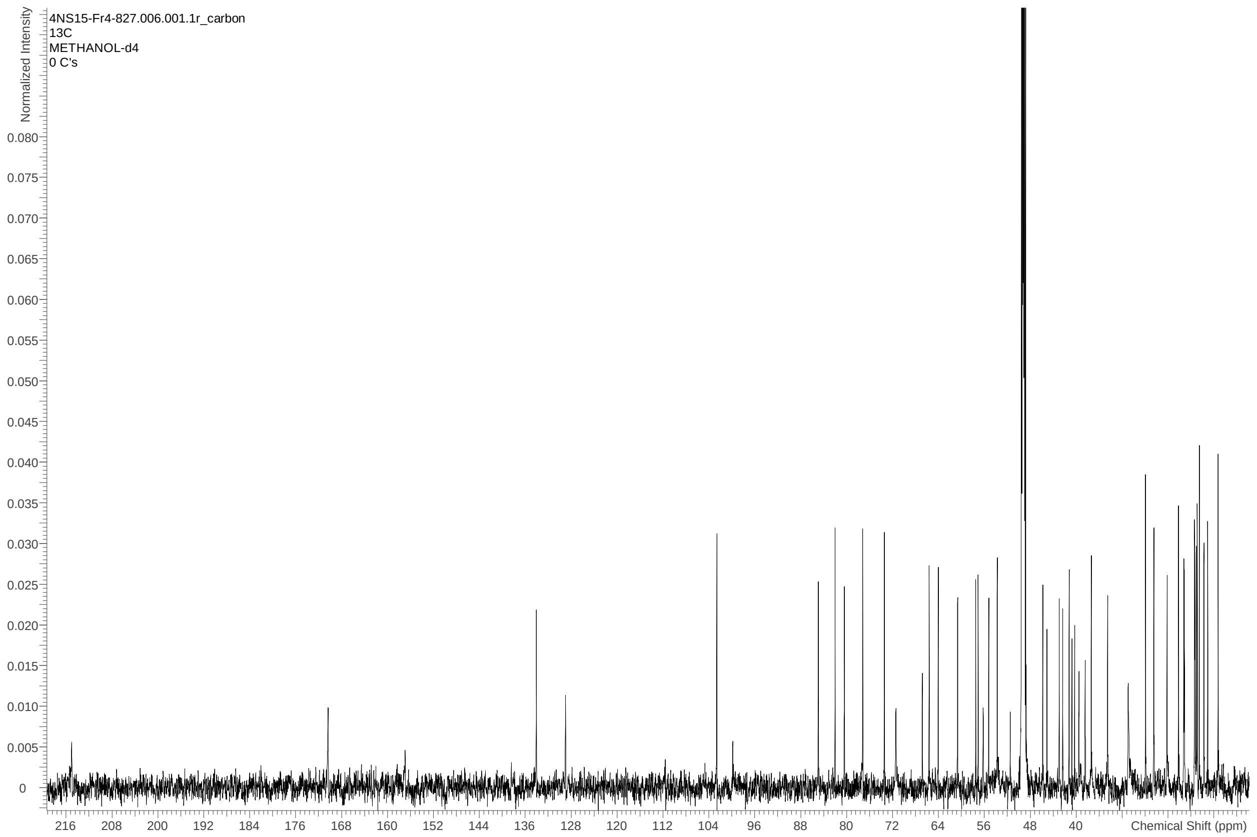

Figure S36. ^{13}C NMR spectrum of persicamidine C (**3**) in CD_3OD (175 MHz)

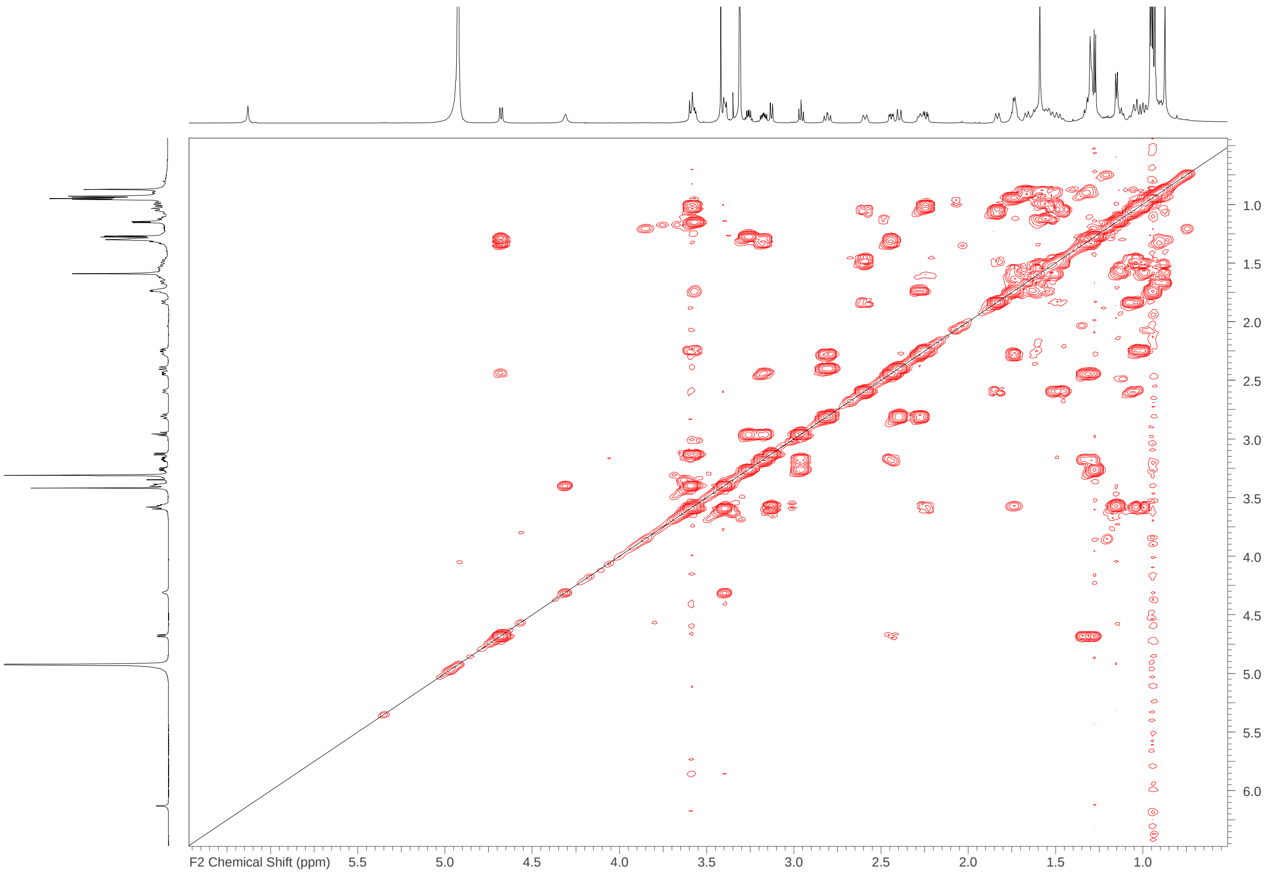

Figure S37. COSY NMR spectrum of persicamidine C (**3**) in CD_3OD (700 MHz)

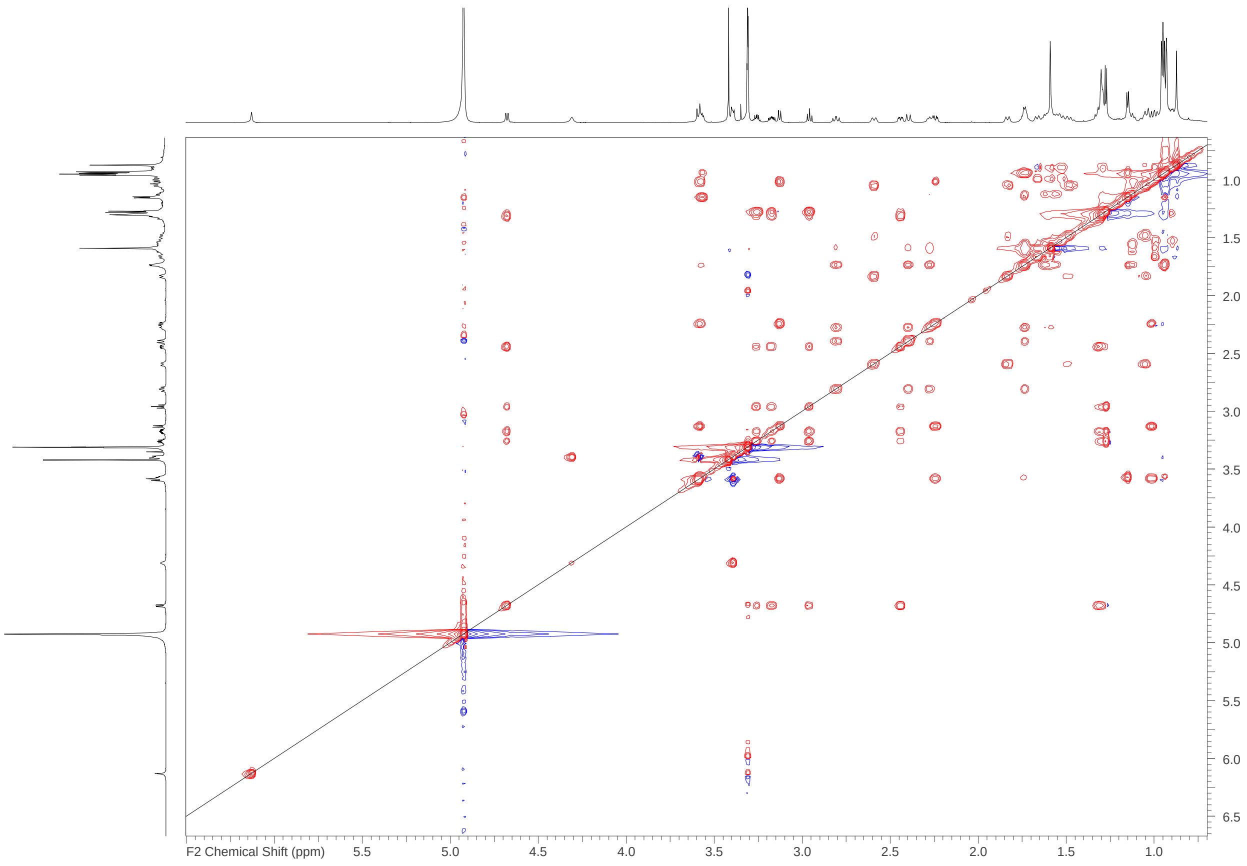

Figure S38. TOCSY NMR spectrum of persicamidine C (**3**) in CD_3OD (700 MHz)

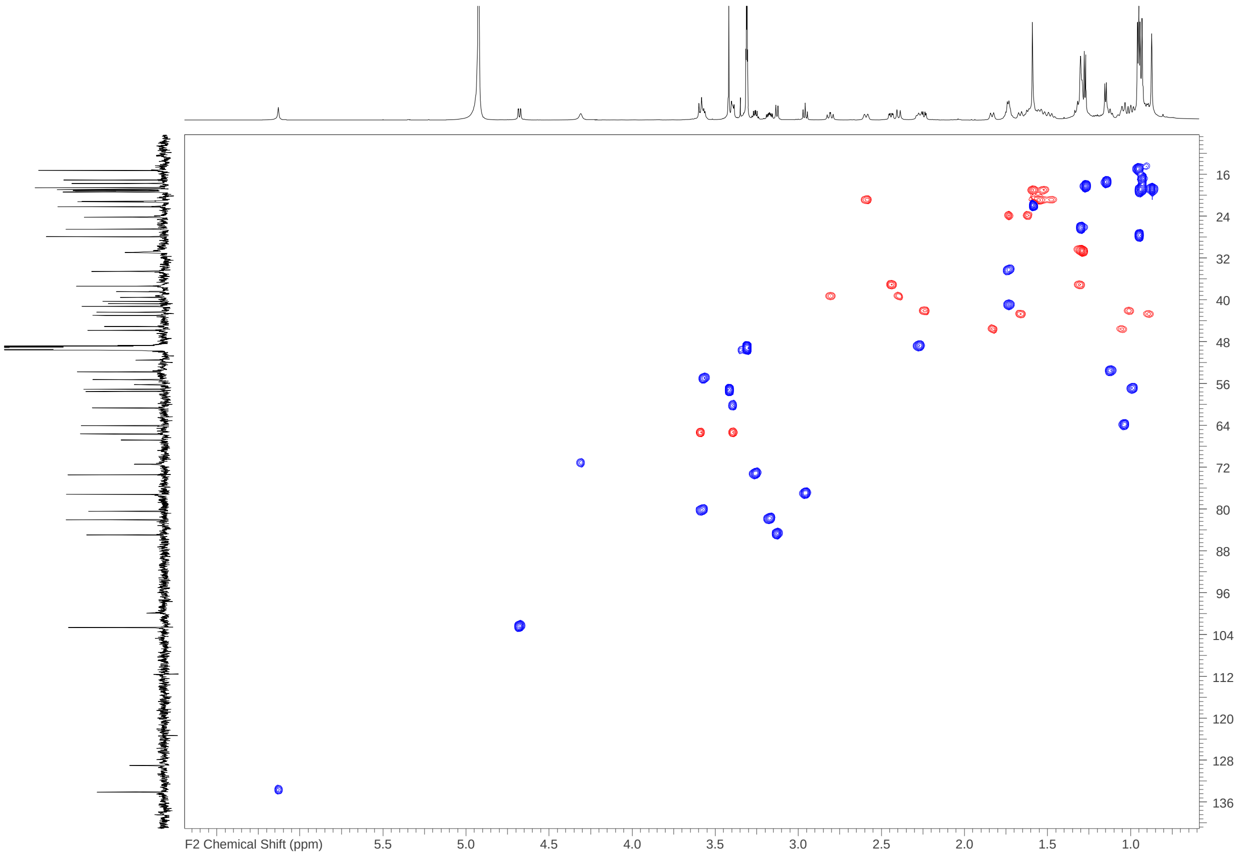

Figure S39. HSQC NMR spectrum of persicamidine C (**3**) in CD_3OD (700 MHz)

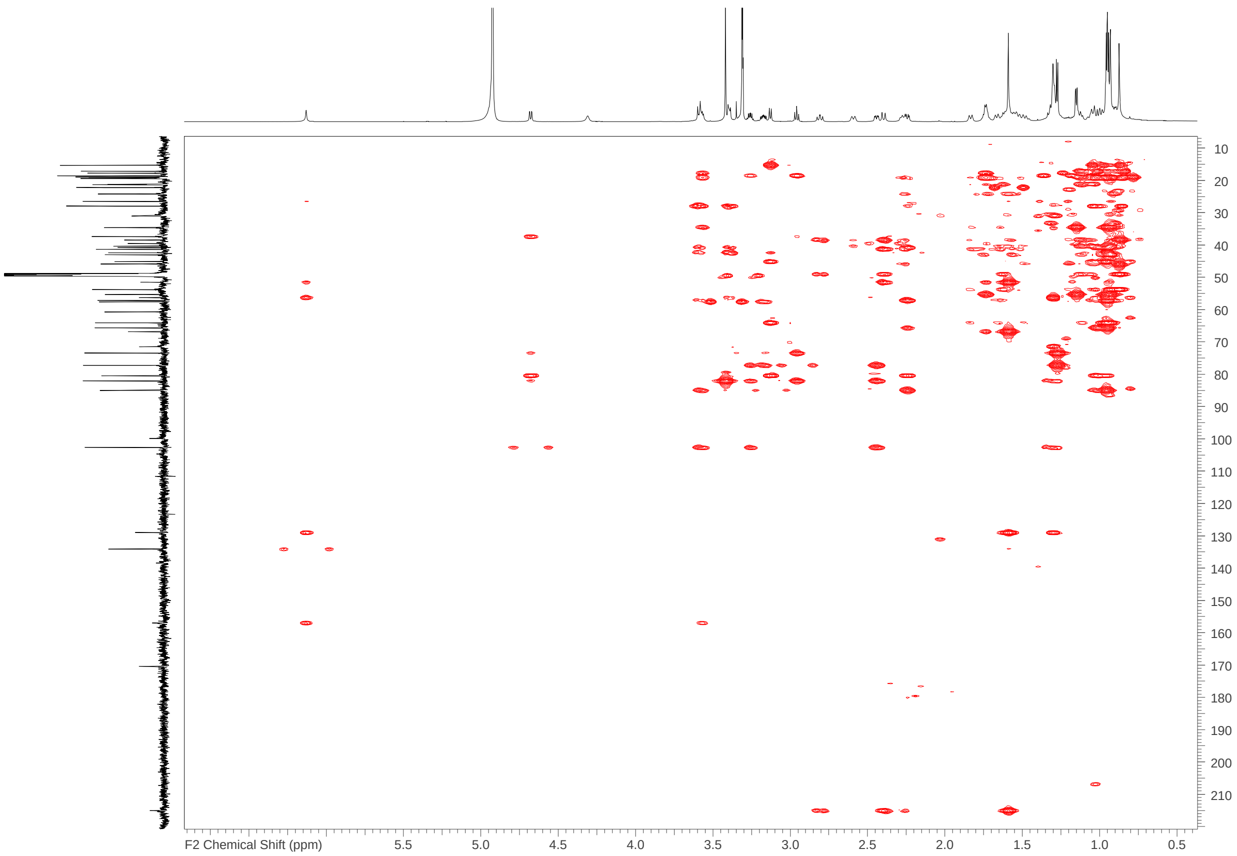

Figure S40. HMBC NMR spectrum of persicamidine C (**3**) in CD_3OD (700 MHz)

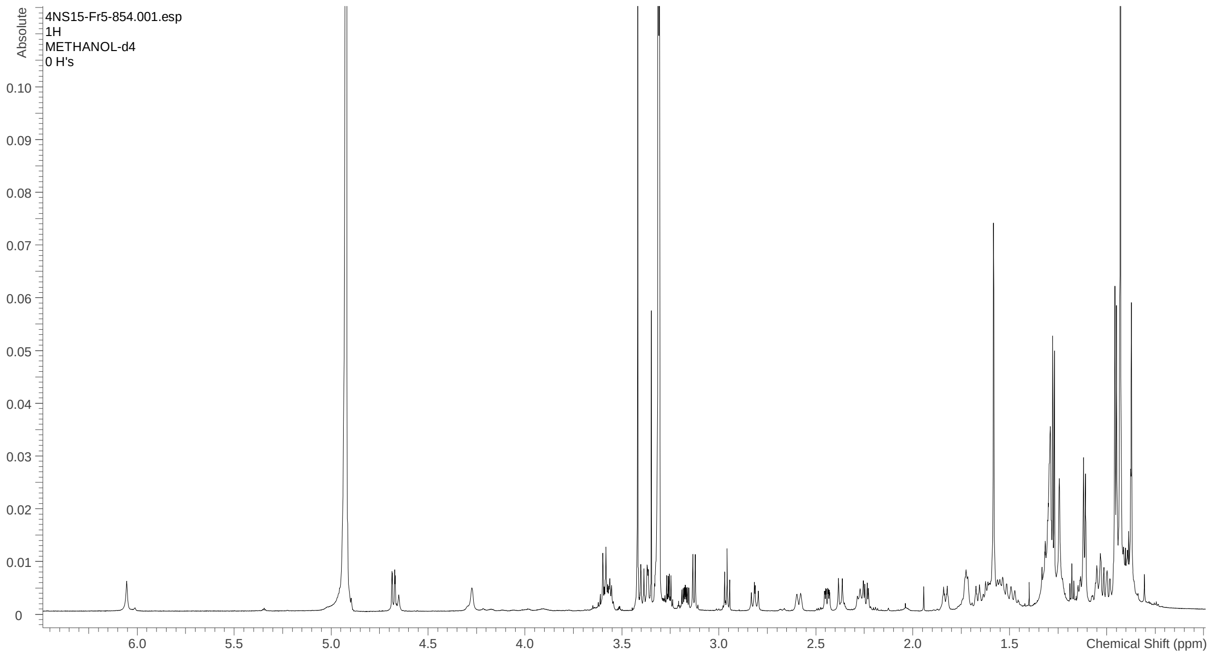

Figure S41. ^{1}H NMR spectrum of persicamidine D (**4**) in CD_3OD (700 MHz)

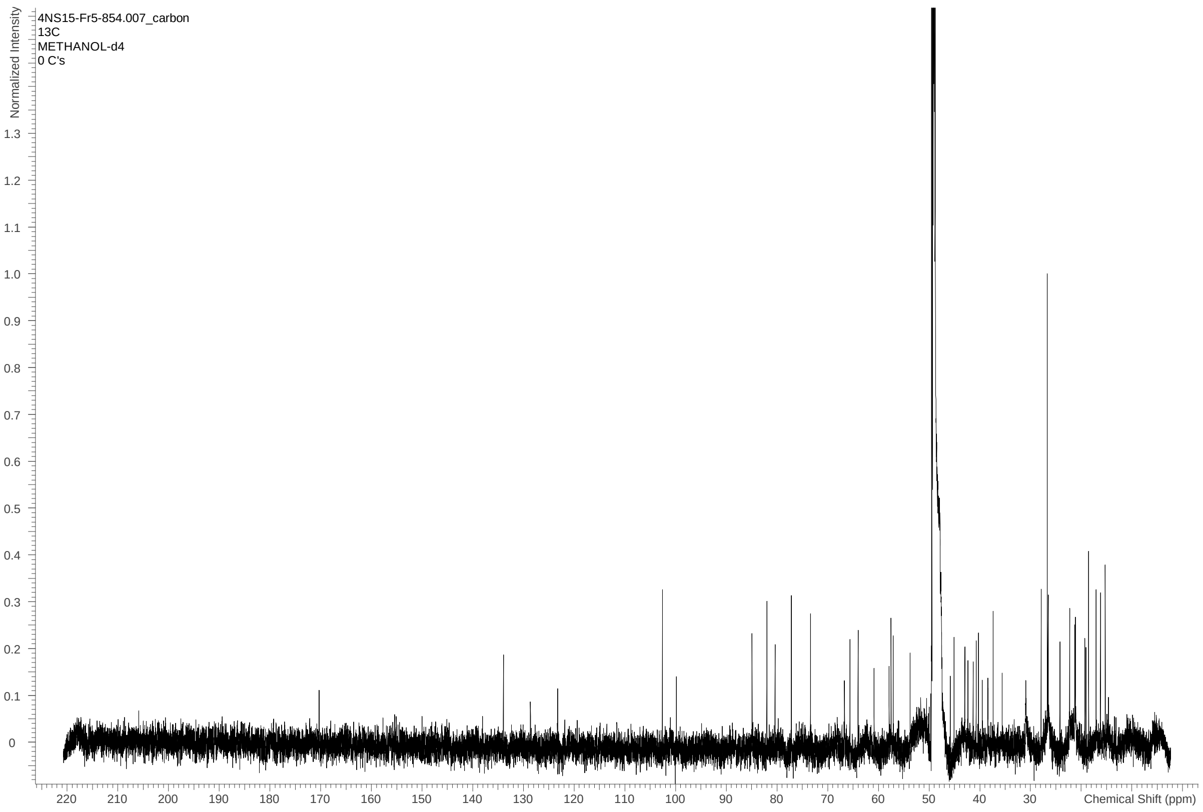

Figure S42. ^{13}C NMR spectrum of persicamidine D (**4**) in CD_3OD (175 MHz)

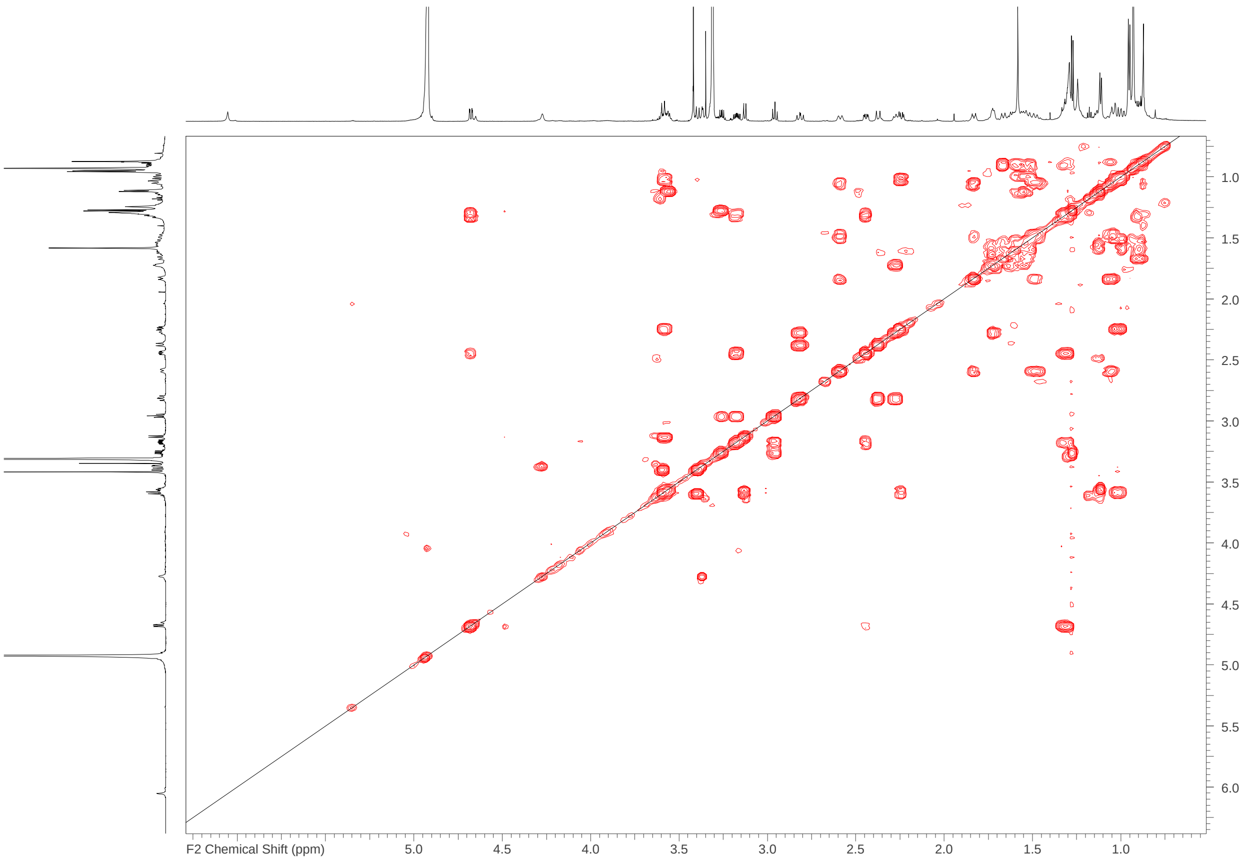

Figure S43. COSY NMR spectrum of persicamidine D (**4**) in CD_3OD (700 MHz)

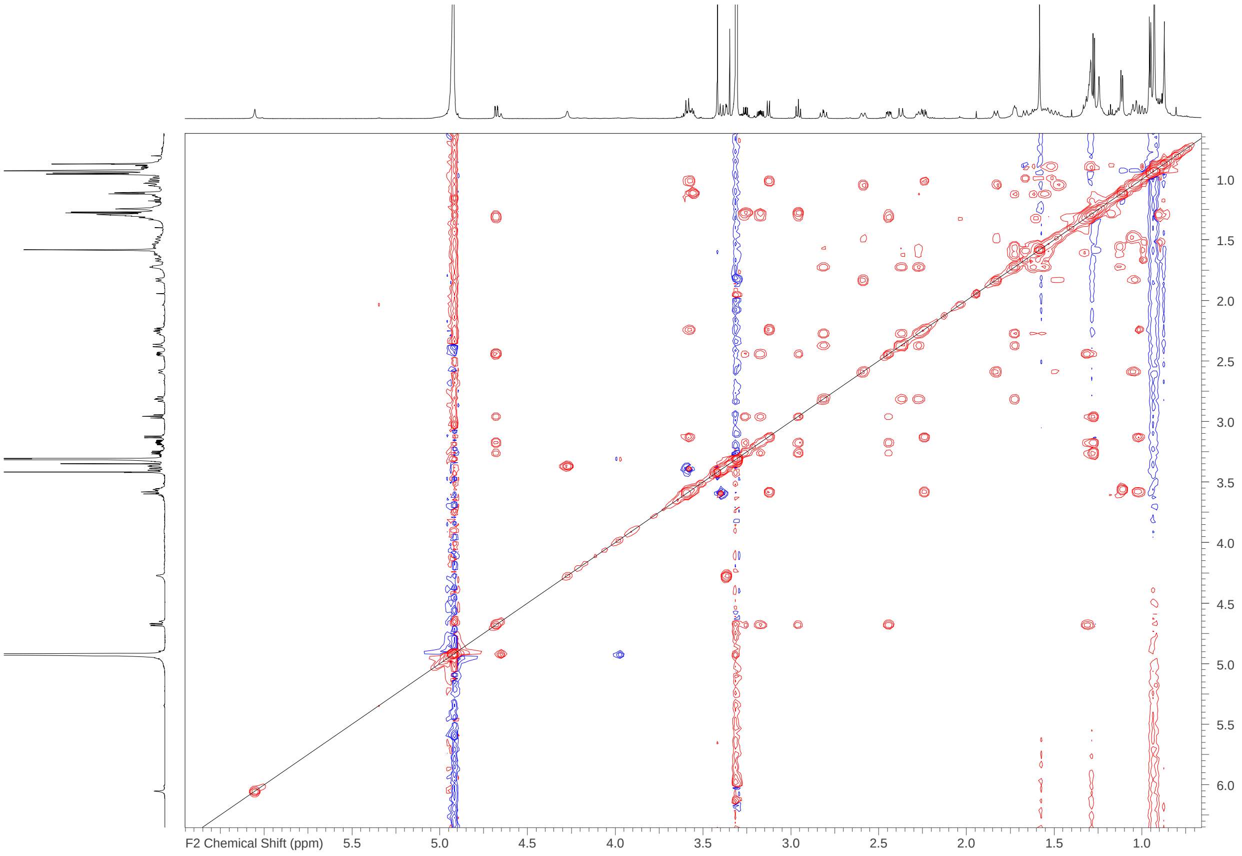

Figure S44. TOCSY NMR spectrum of persicamidine D (**4**) in CD_3OD (700 MHz)

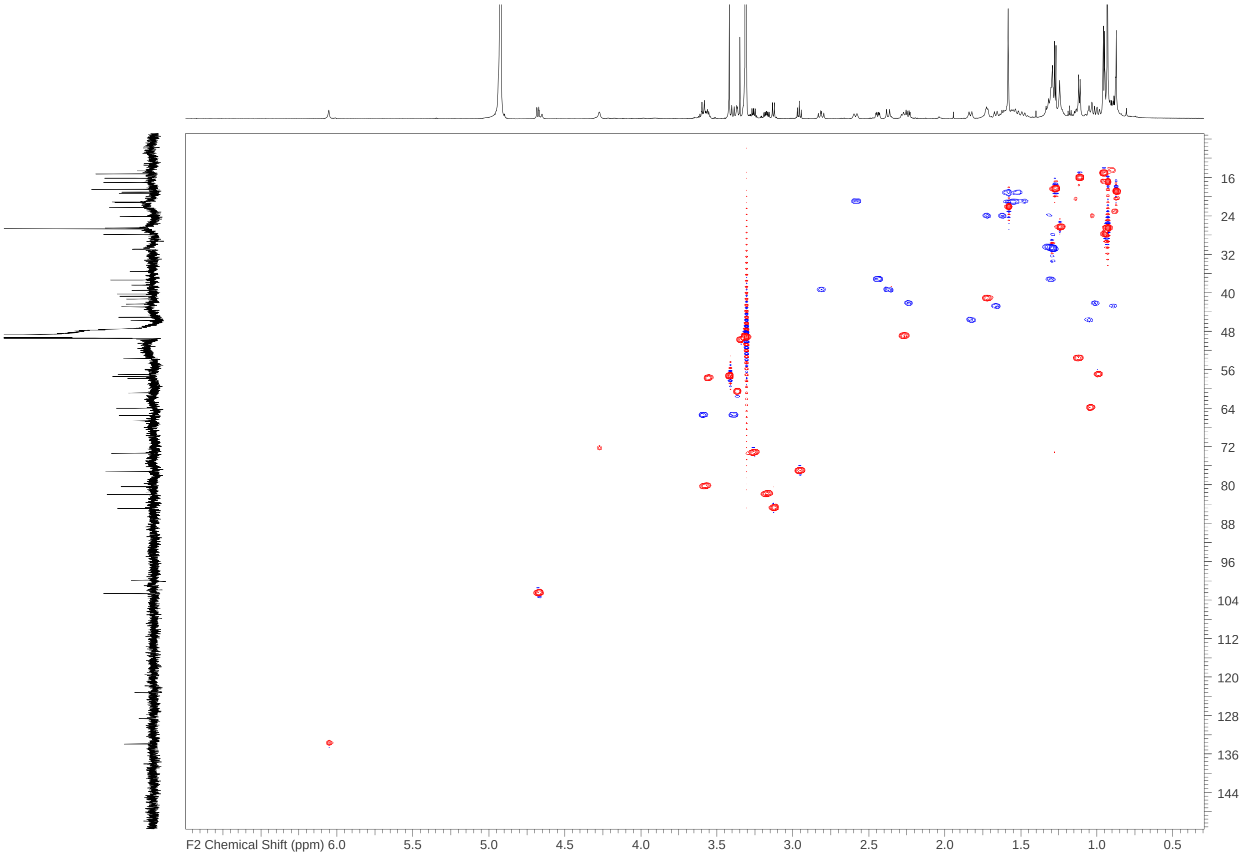

Figure S45. HSQC NMR spectrum of persicamidine D (**4**) in CD_3OD (700 MHz)

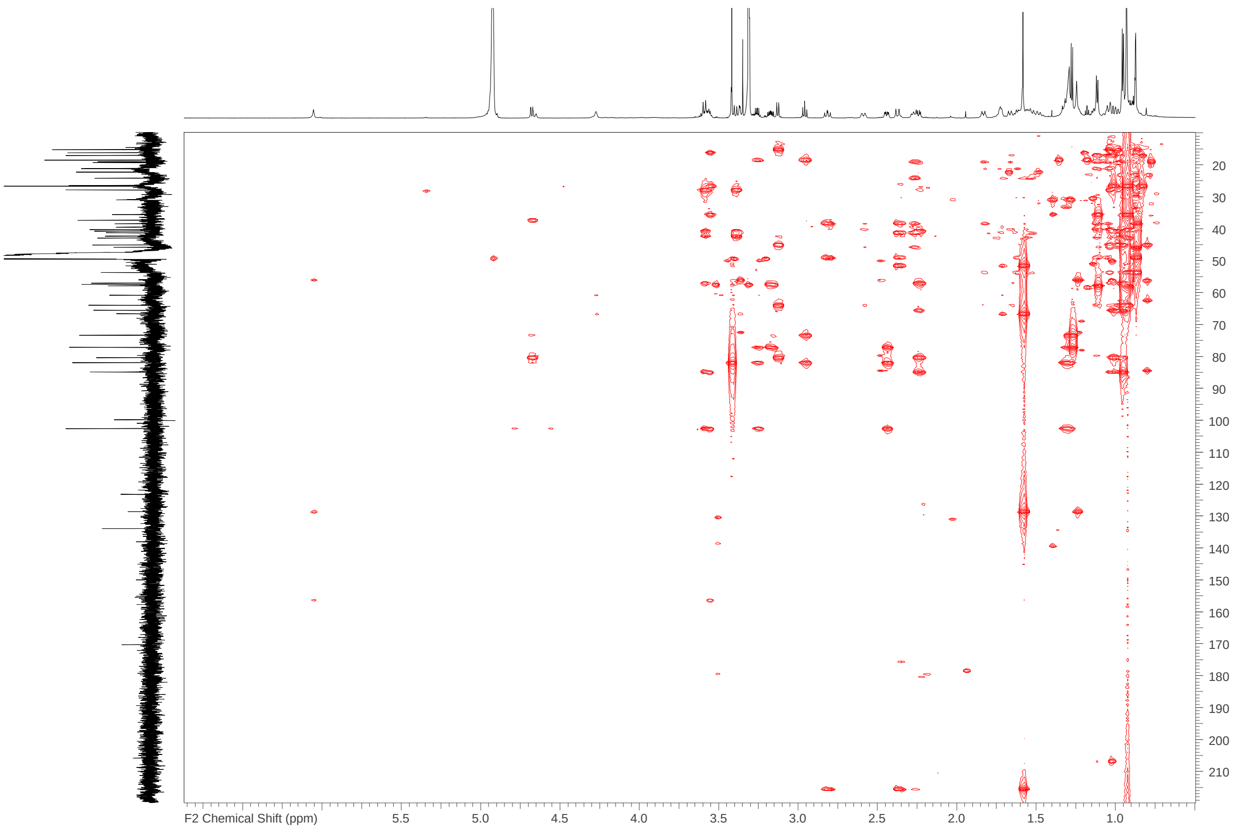

Figure S46. HMBC NMR spectrum of persicamidine D (**4**) in CD_3OD (700 MHz)

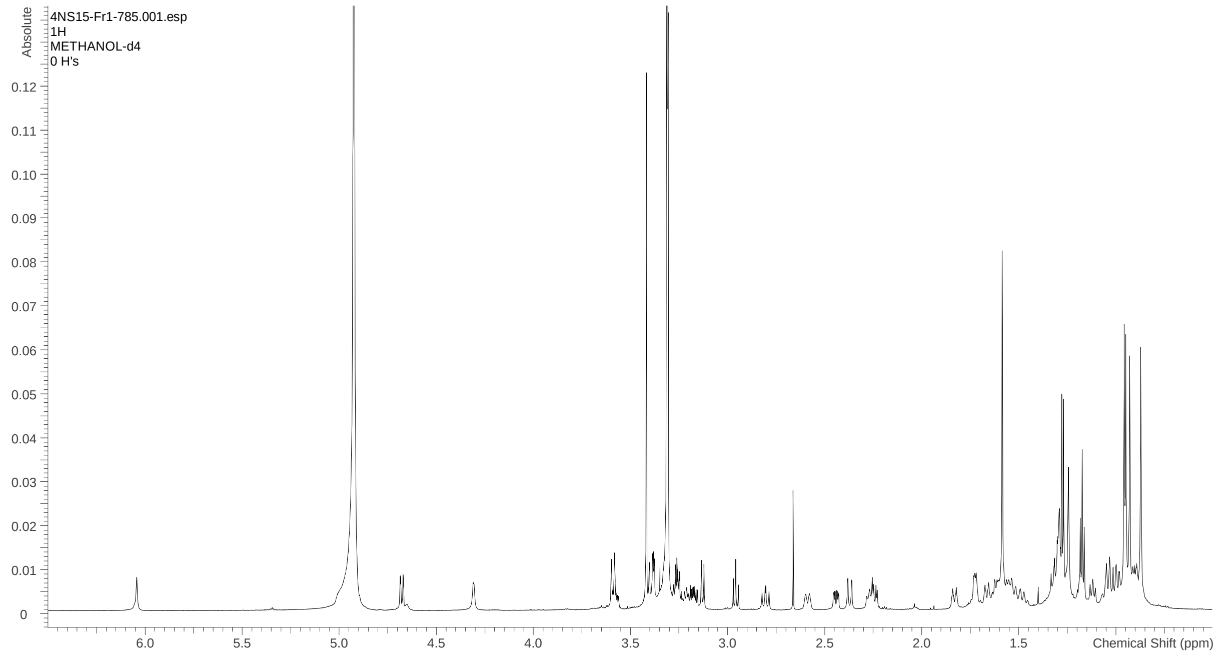

Figure S47. ^{1}H NMR spectrum of persicamidine E (**5**) in CD_3OD (700 MHz)

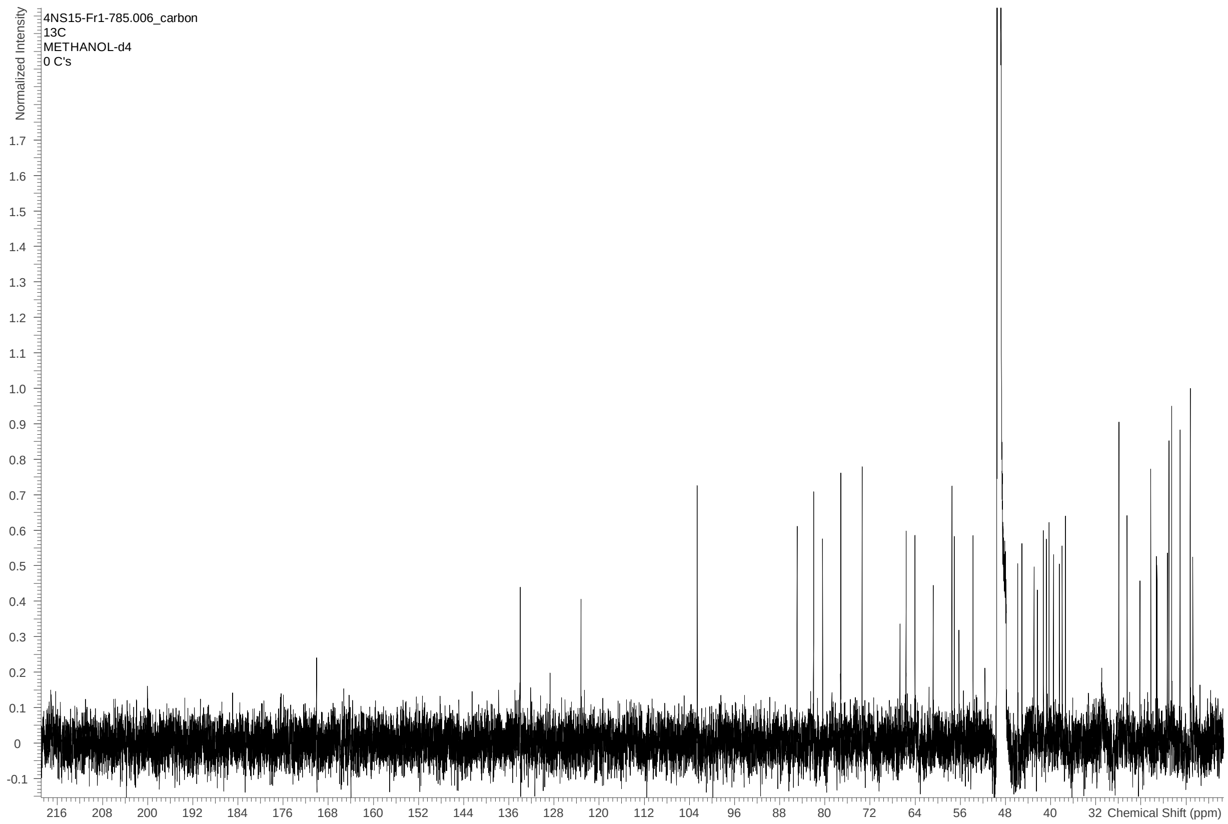

Figure S48. ^{13}C NMR spectrum of persicamidine E (**5**) in CD_3OD (175 MHz)

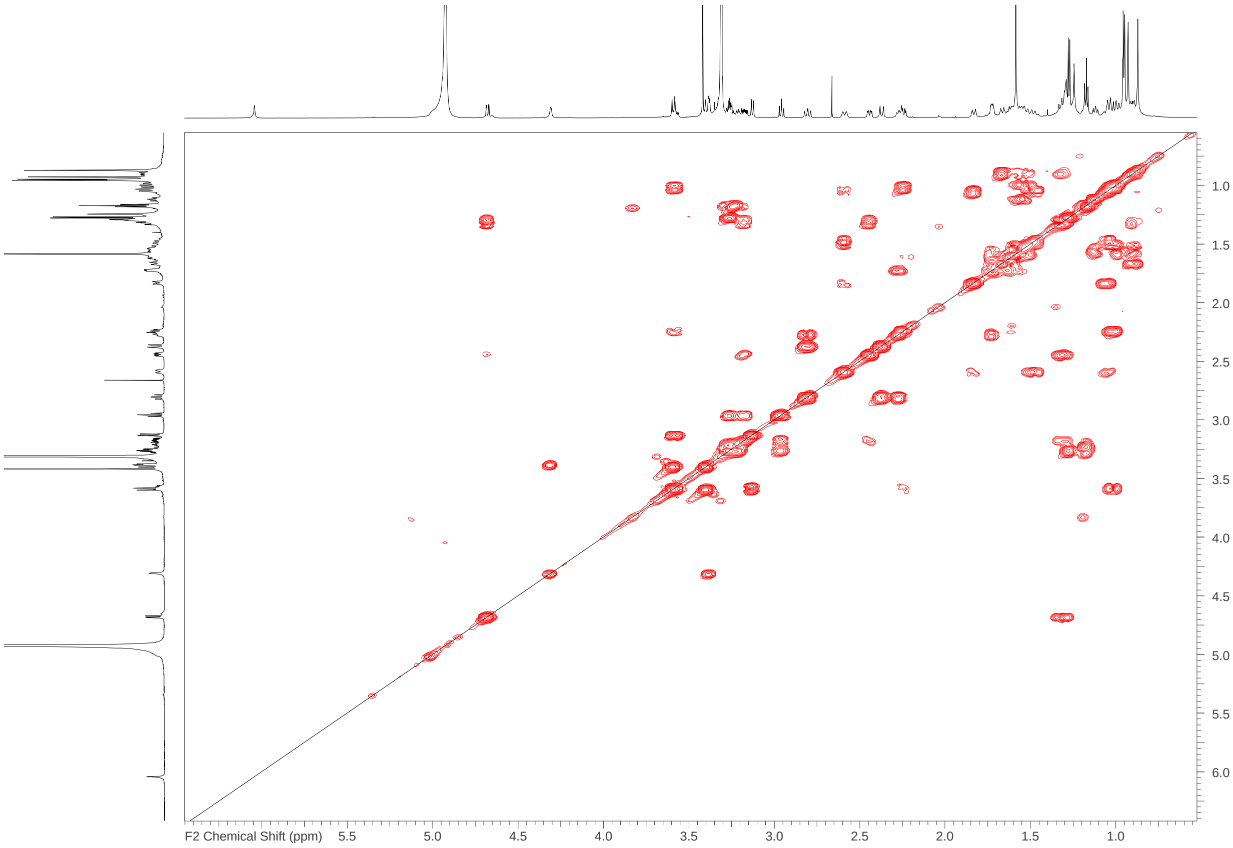

Figure S49. COSY NMR spectrum of persicamidine E (**5**) in CD_3OD (700 MHz)

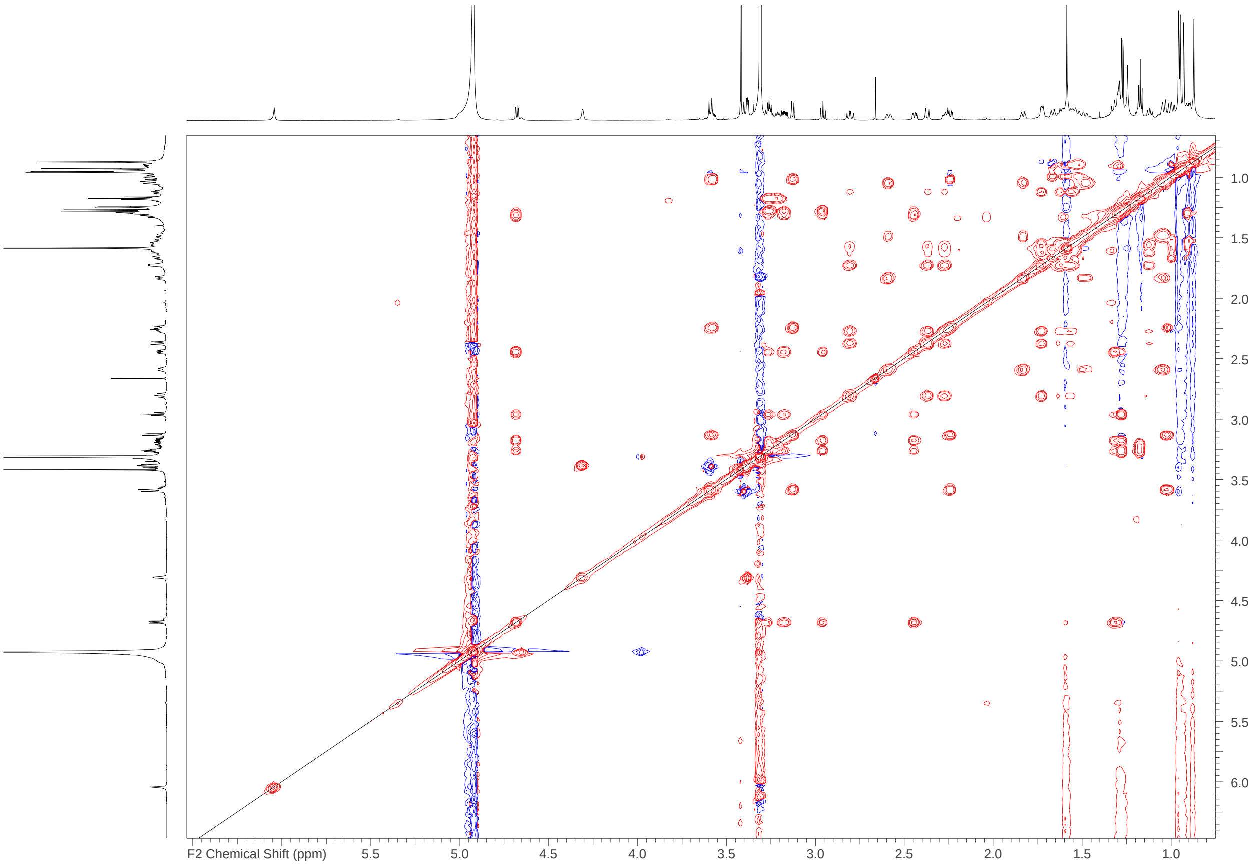

Figure S50. TOCSY NMR spectrum of persicamidine E (**5**) in CD_3OD (700 MHz)

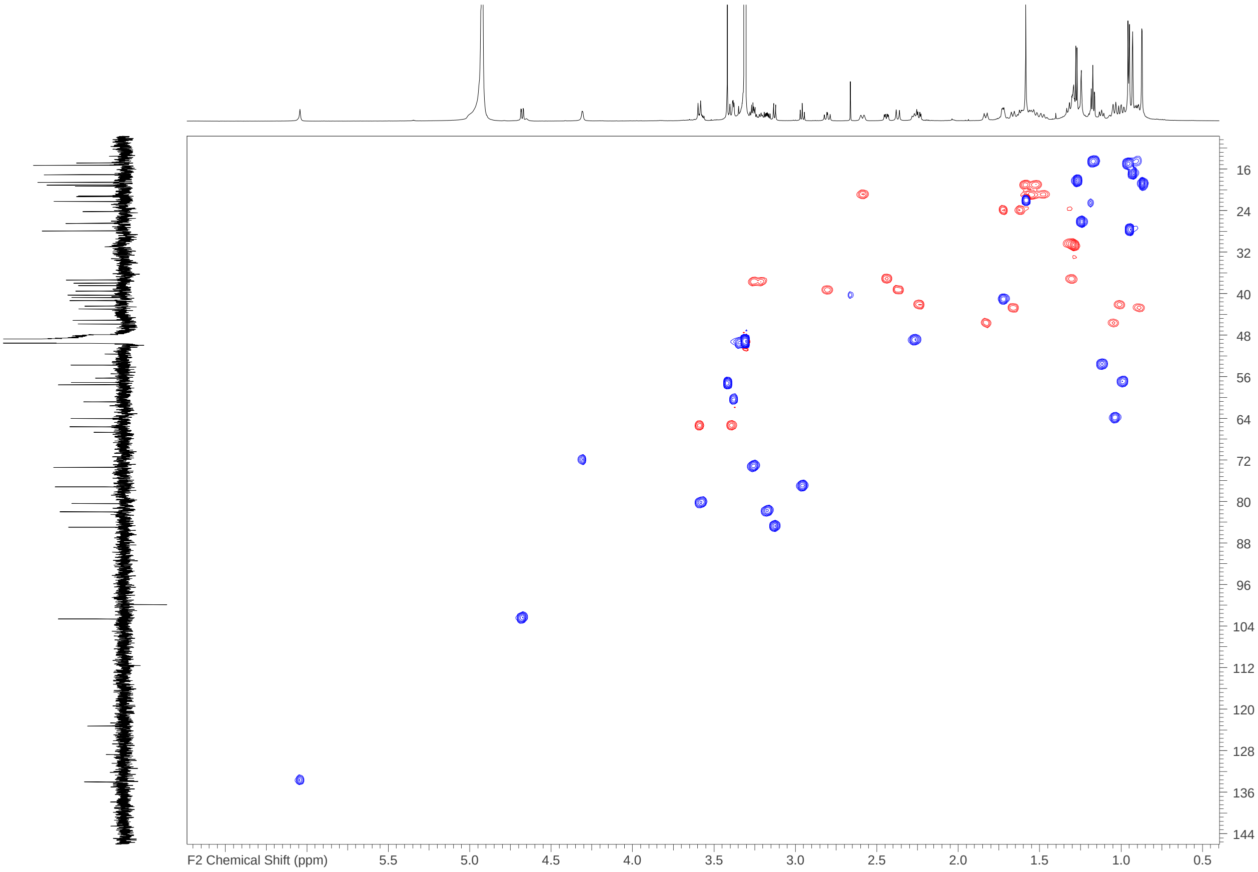

Figure S51. HSQC NMR spectrum of persicamidine E (**5**) in CD_3OD (700 MHz)

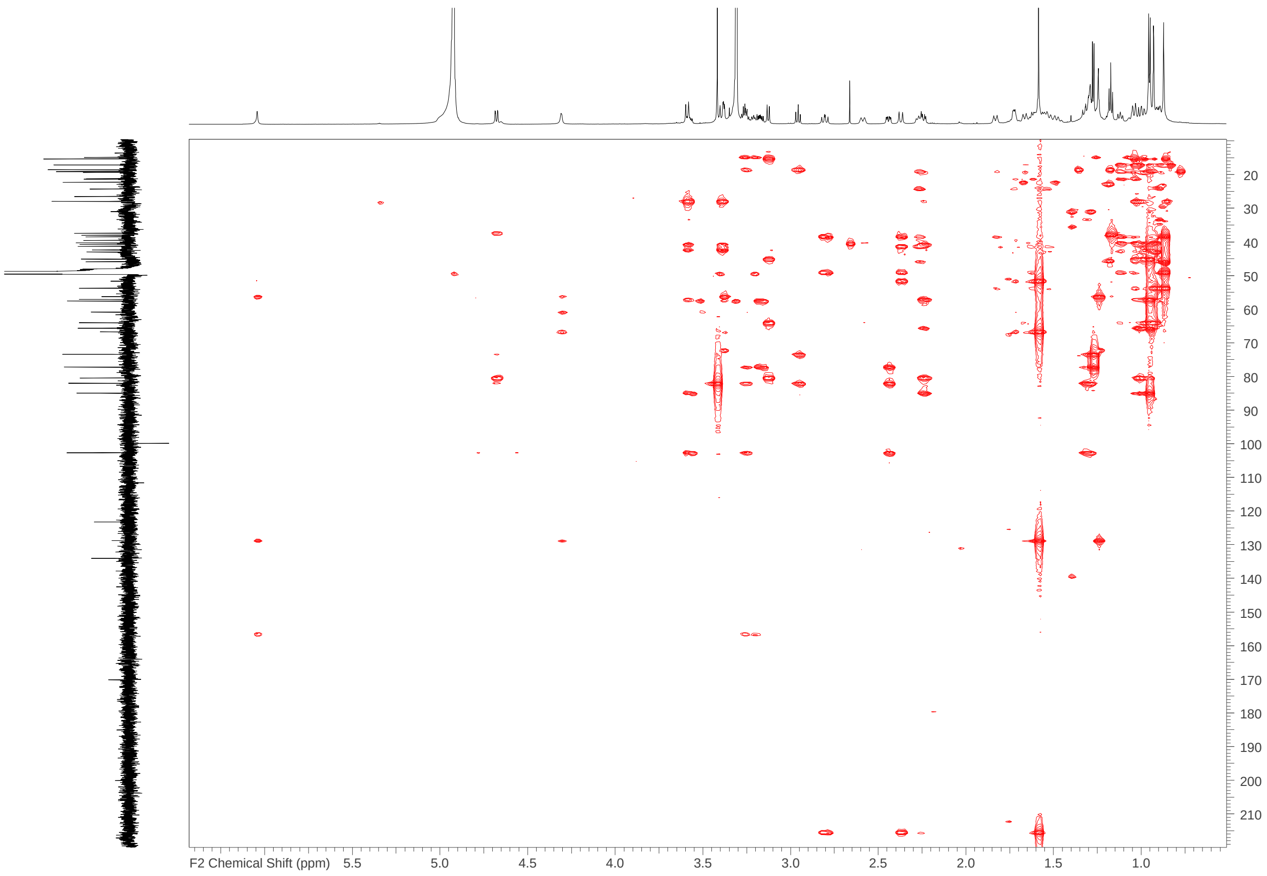

Figure S52. HMBC NMR spectrum of persicamidine E (**5**) in CD_3OD (700 MHz)

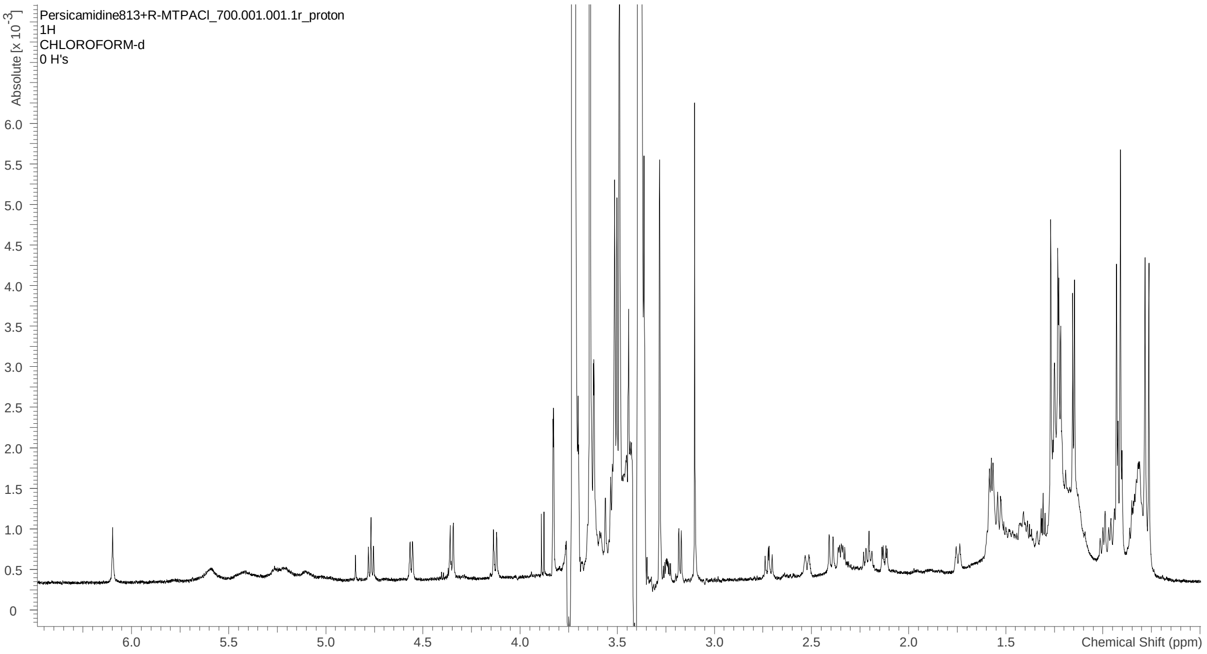

Figure S53. ^{1}H NMR spectrum of (*S*)-Mosher ester of persicamidine A in $CDCl_3$ (700 MHz)

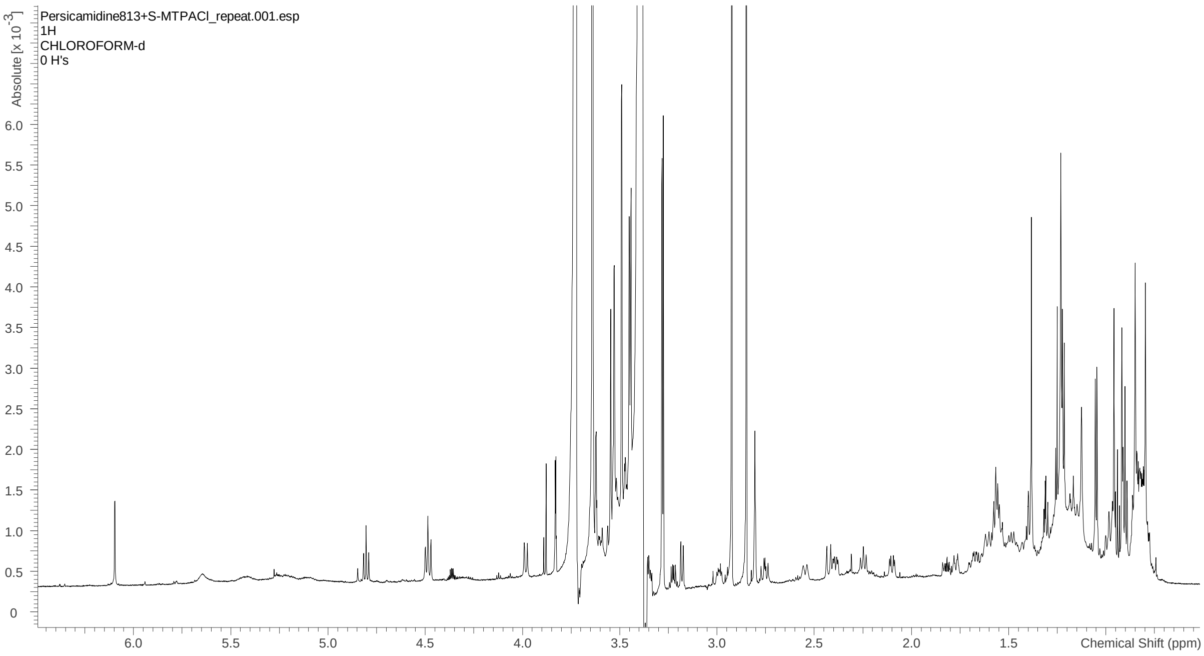

Figure S54. ^{1}H NMR spectrum of (*R*)-Mosher ester of persicamidine A in $CDCl_3$ (700 MHz)

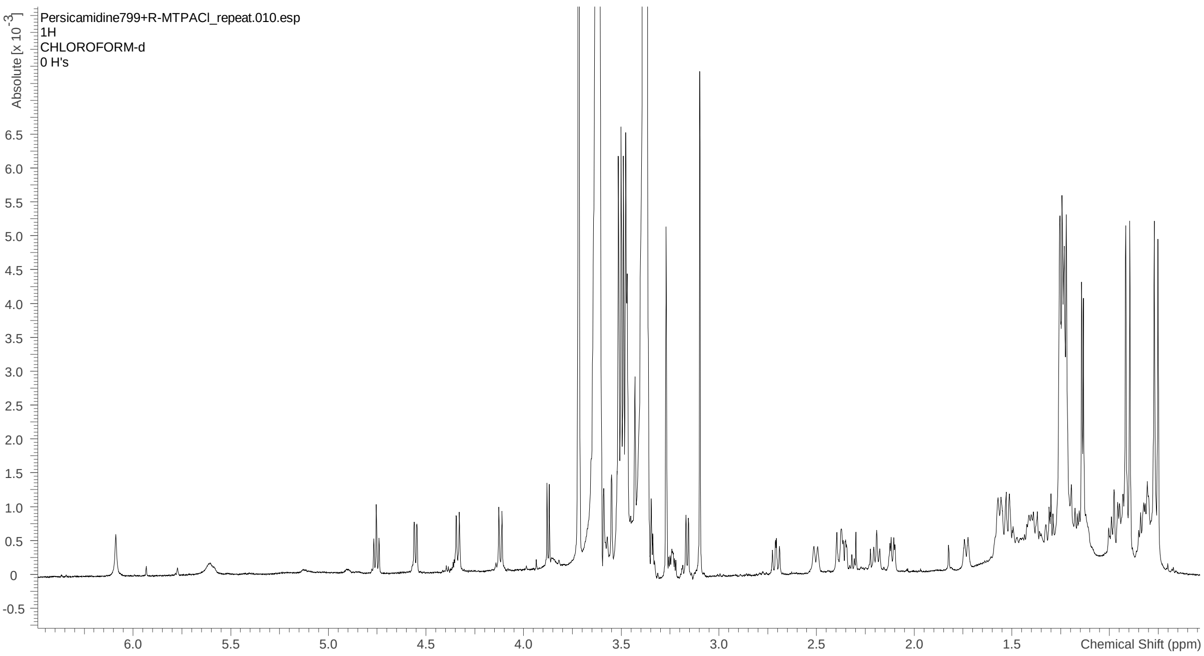

Figure S55. ^{1}H NMR spectrum of (*S*)-Mosher ester of persicamidine B in $CDCl_3$ (700 MHz)

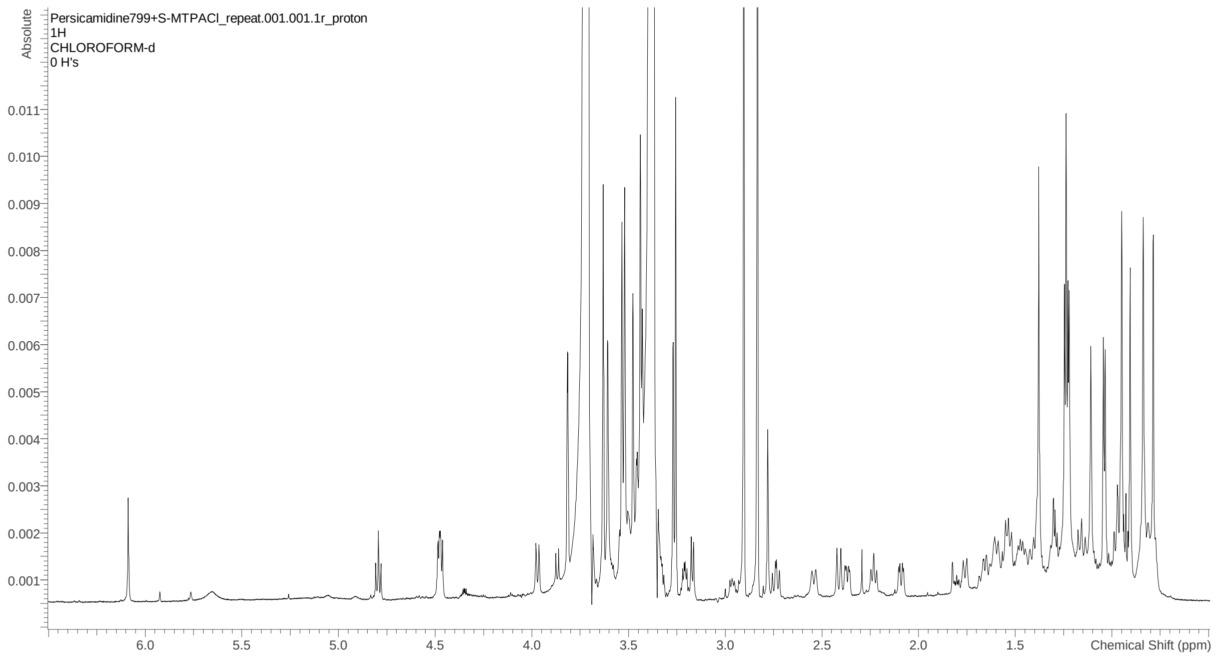

Figure S56. ^{1}H NMR spectrum of (*R*)-Mosher ester of persicamidine B in $CDCl_3$ (700 MHz)

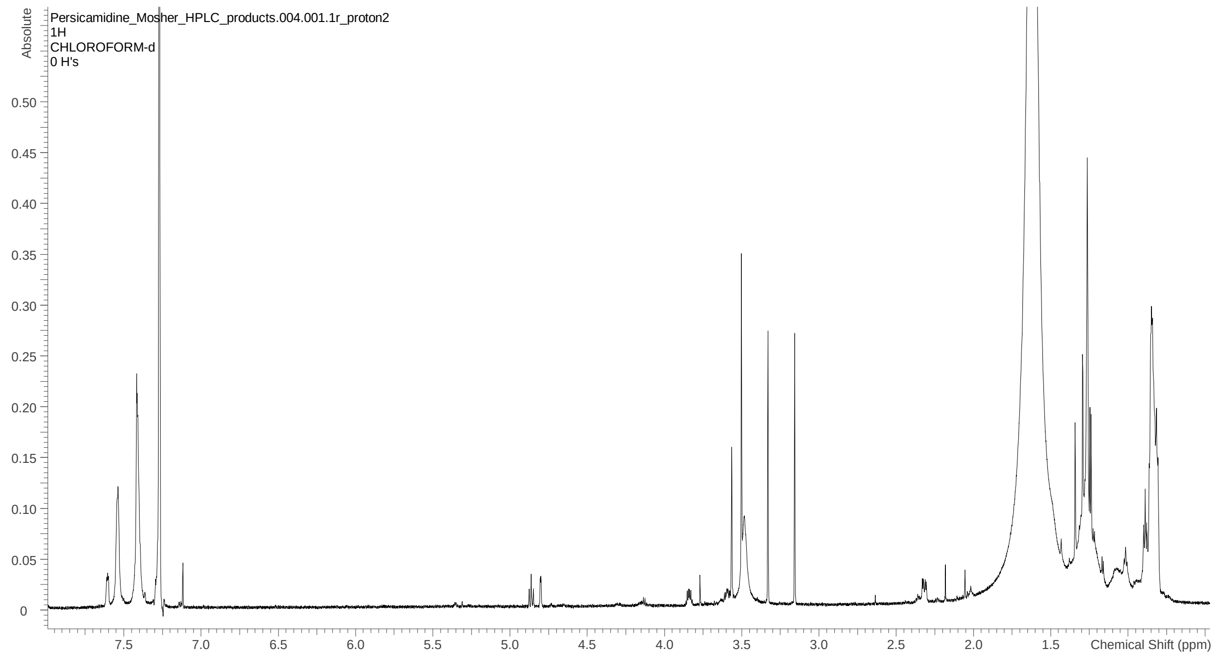

Figure S57. ^{1}H NMR spectrum of (*S*)-Mosher ester of the cleaved glycon derivative in $CDCl_3$ (700 MHz)

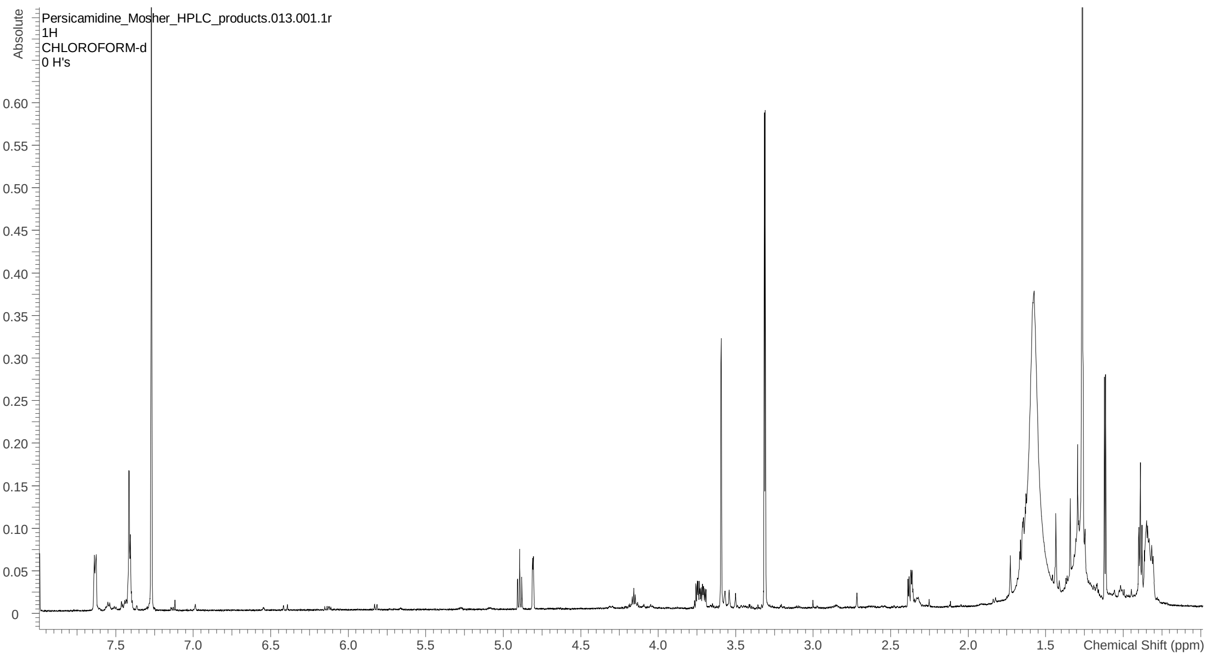

Figure S58. ^{1}H NMR spectrum of (*R*)-Mosher ester of the cleaved glycon derivative in $CDCl_3$ (700 MHz)

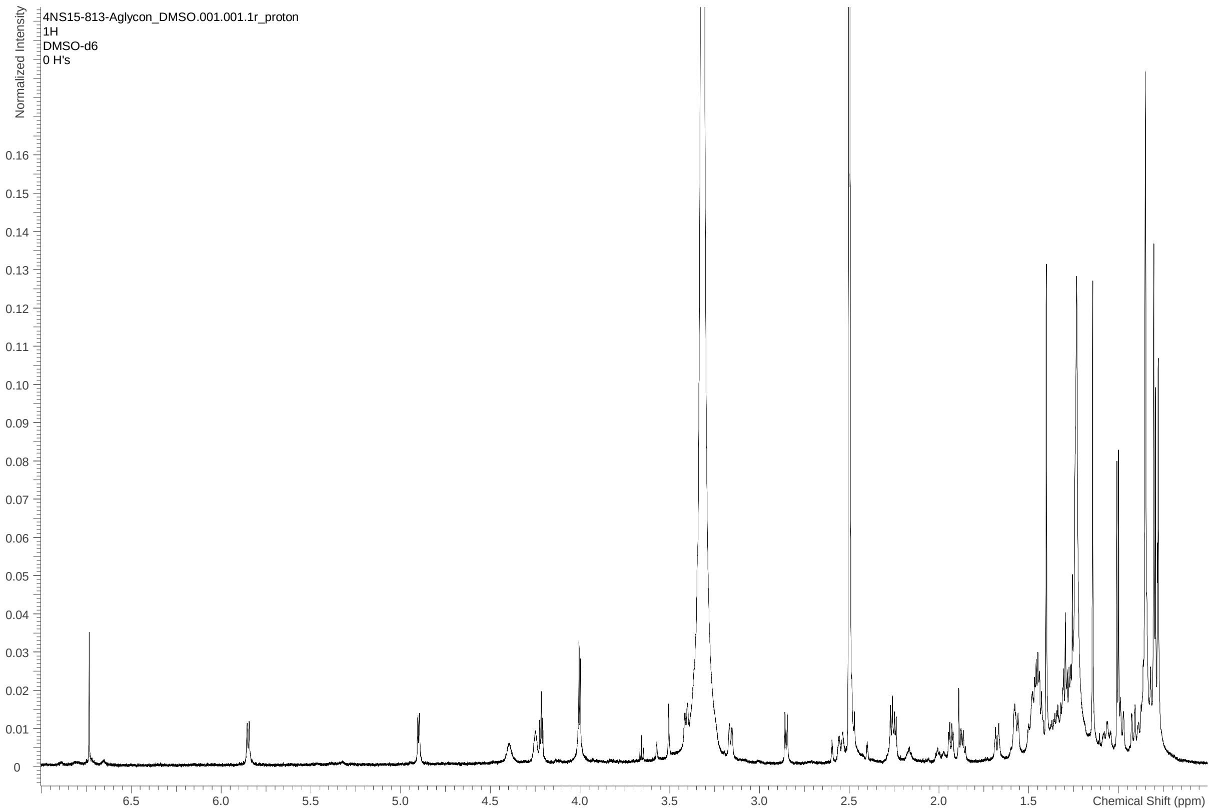

Figure S59. ^{1}H NMR spectrum of persicamidine A aglycon formed via Payne-type rearrangement in DMSO-d_6 (700 MHz)

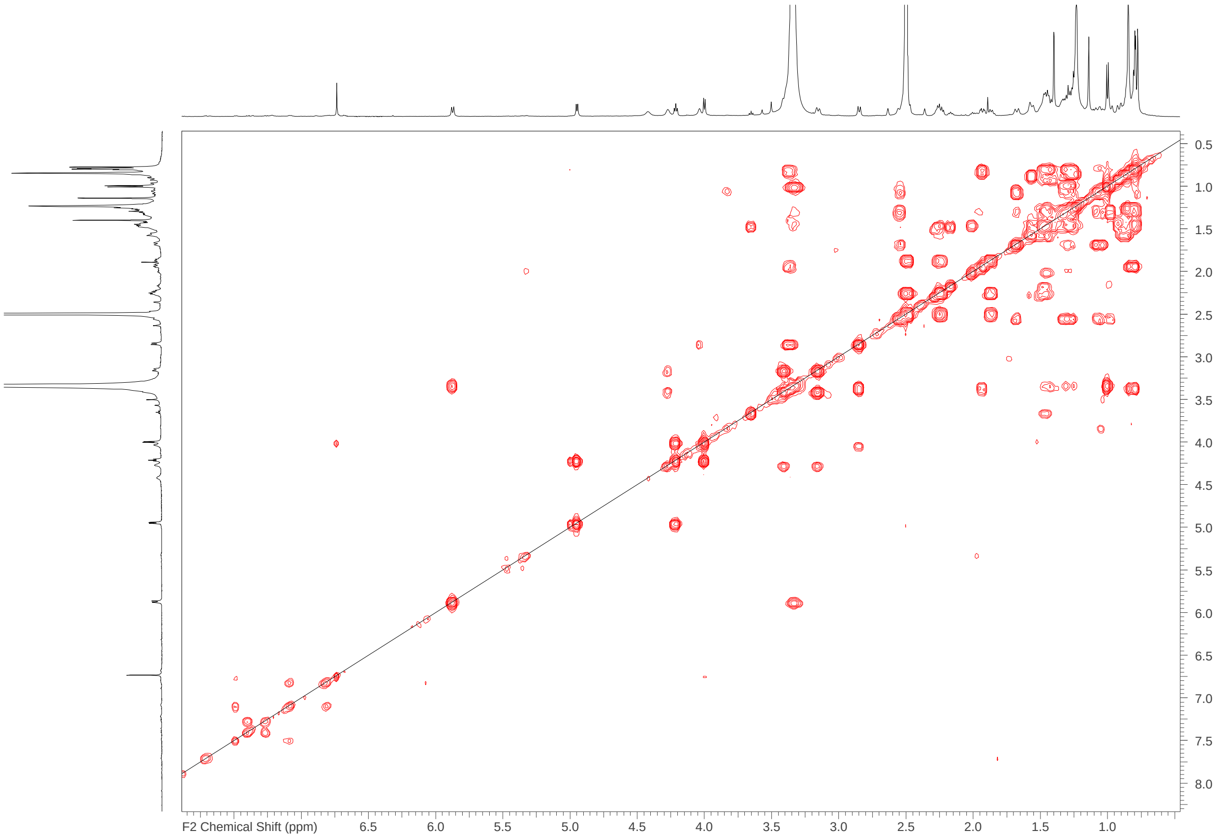

Figure S60. COSY NMR spectrum of persicamidine A aglycon formed via Payne-type rearrangement in DMSO-d_6 (500 MHz)

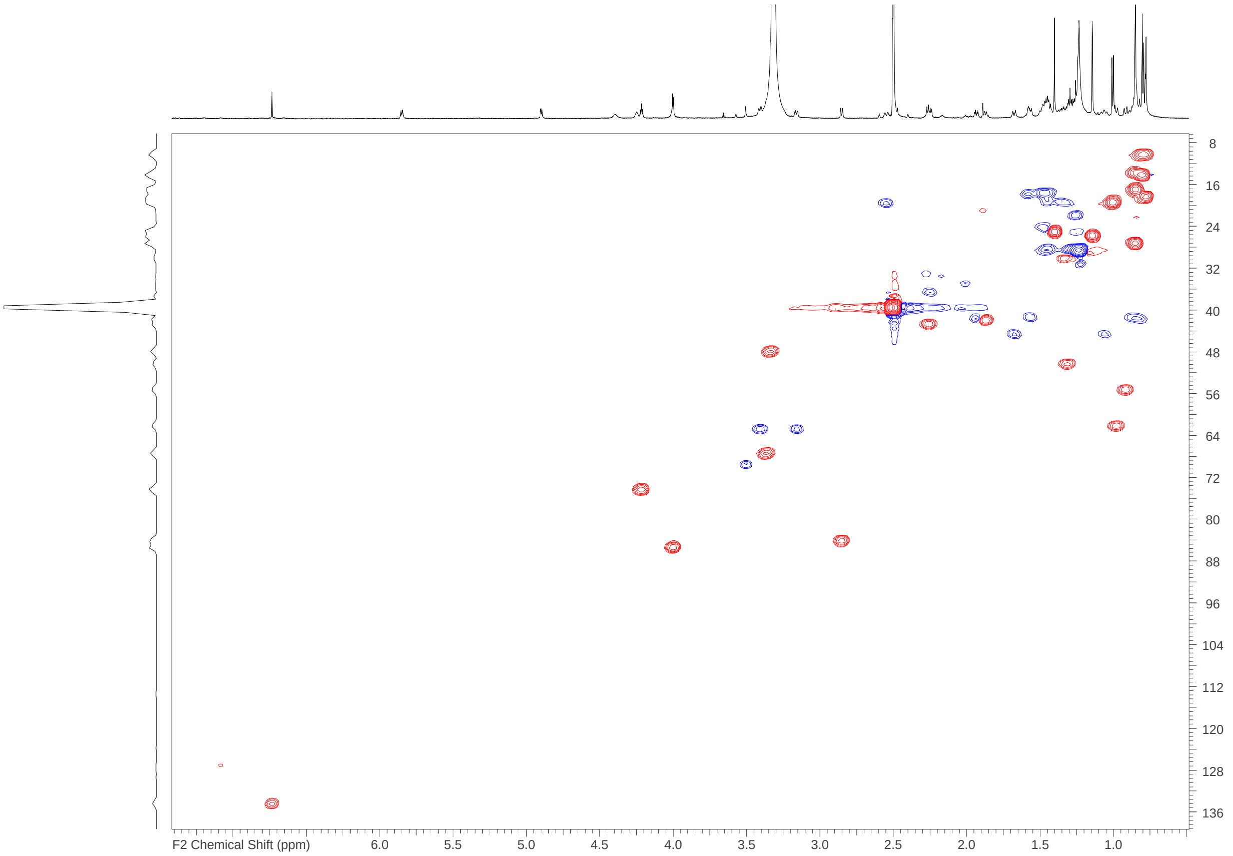

Figure S61. HSQC NMR spectrum of persicamidine A aglycon formed via Payne-type rearrangement in DMSO-d_6 (700 MHz)

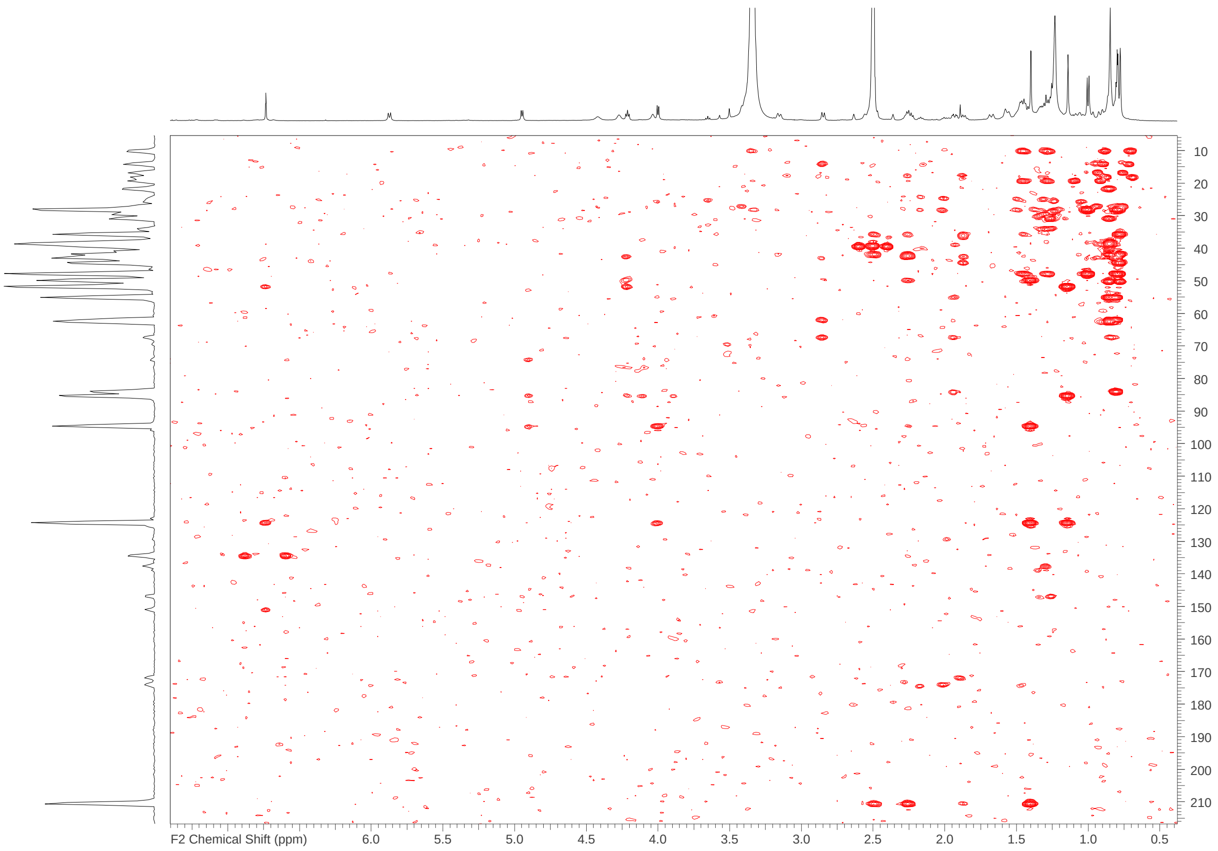

Figure S62. HMBC NMR spectrum of persicamidine A aglycon formed via Payne-type rearrangement in DMSO-d_6 (700 MHz)

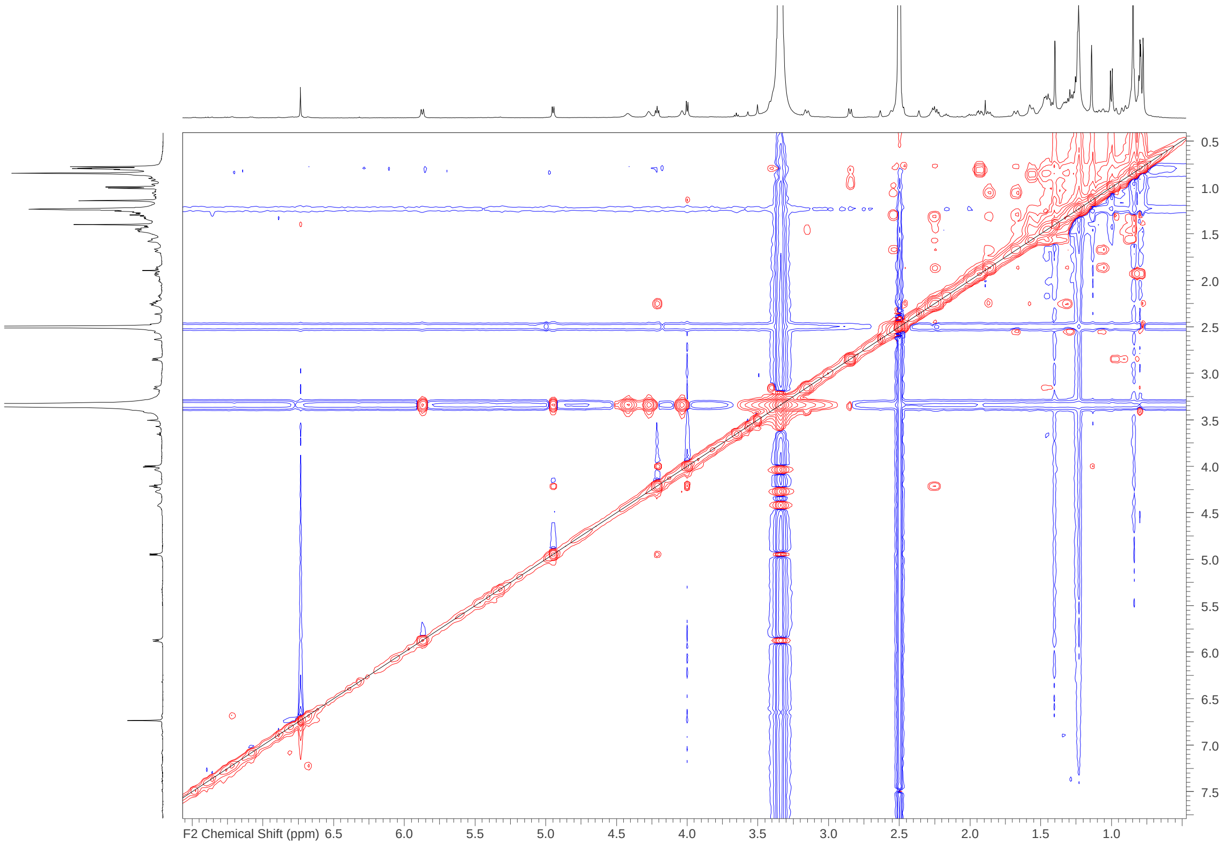

Figure S63. NOESY NMR spectrum of persicamidine A aglycon formed via Payne-type rearrangement in DMSO-d_6 (500 MHz)

References

[1] N. Safaei, I. Nouioui, Y. Mast, N. Zaburannyi, M. Rohde, P. Schumann, R. Müller, J. Wink, *Int. J. Syst. Evol. Microbiol.* **2021**, *71*, 004625.

[2] K. Fujii, Y. Ikai, H. Oka, M. Suzuki, K. Harada, *Anal. Chem.* **1997**, *69*, 5146.

[3] T. R. Hoye, C. S. Jeffrey, F. Shao, *Nat. Protoc.* **2007**, *2*, 2451.

[4] V. B. Birman, S. J. Danishefsky, *J. Am. Chem. Soc.* **2002**, *124*, 2080.

[5] S. H. E. van den Worm, K. K. Eriksson, J. C. Zevenhoven, F. Weber, R. Züst, T. Kuri, R. Dijkman, G. Chang, S. G. Siddell, E. J. Snijder et al., *PloS one* **2012**, *7*, e32857.

[6] M. Widera, A. Wilhelm, T. Toptan, J. M. Raffel, E. Kowarz, F. Roesmann, F. Grözinger, A. L. Siemund, V. Luciano, M. Külp et al., *Front. Microbiol.* **2021**, *12*, 701198.

[7] X. Xie, A. Muruato, K. G. Lokugamage, K. Narayanan, X. Zhang, J. Zou, J. Liu, C. Schindewolf, N. E. Bopp, P. V. Aguilar et al., *Cell Host Microbe* **2020**, *27*, 841.e3.

Author Contributions

LK: data acquisition, data analysis, investigation, writing of original draft – all lead contribution
EO: compound isolation, data acquisition, data analysis, validation, writing of original draft – all equal contribution
AK: data acquisition, data analysis, writing of original draft – equal contribution
NS: strain isolation, compound isolation – lead contribution
SHK: data analysis biological activity, validation, project administration, writing of original draft – lead contribution: project administration, rest: minor contribution
APG: data acquisition biological activity, data analysis, validation, writing of original draft – equal contribution, writing: minor contribution
SH: data acquisition biological activity, data analysis, validation – all equal contribution
UR: data acquisition biological activity, data analysis, validation, writing of original draft – equal contribution, writing: minor contribution
LCS: data acquisition biological activity, data analysis, validation – equal contribution
CF: project planning, data analysis – equal contribution
JW: project planning, strain isolation– all lead contribution
TP: project planning biological activity, data analysis– all lead contribution
RM: project planning, funding acquisition, project administration, validation – all lead contribution
